# Decoding Double Layer Dynamics for ${\mathrm{CO}}_{2}$ Electroreduction over Cu

**DOI:** 10.1002/anie.202423177

**Published:** 2025-06-13

**Authors:** Daniel Sinausia, Noam Zisser, Thierry Kilian Slot, David Eisenberg, Florian Meirer, Charlotte Vogt

**Affiliations:** ^1^ Schulich Faculty of Chemistry, Resnick Sustainability Center for Catalysis, and Grand Technion Energy Program Technion ‐ Israel Institute of Technology Haifa 3200002 Israel; ^2^ Inorganic Chemistry and Catalysis Group Institute for Sustainable and Circular Chemistry and Debye Institute for Nanomaterials Science Utrecht University Universiteitsweg 99 Utrecht 3584 CG The Netherlands

**Keywords:** Carbon dioxide electroreduction, ${\mathrm{CO}}_{2}\mathrm{RR}$, Electric double layer, Water structure reorganization, Dynamic response spectroscopy, DRS

## Abstract

Understanding the nature and role of the electric double layer (EDL) at electrocatalytic interfaces and its dynamic evolution, is critical to optimizing electrochemical processes such as the carbon dioxide reduction reaction (${\mathrm{CO}}_{2}\mathrm{RR}$). Despite its postulated significant influence on ${\mathrm{CO}}_{2}\mathrm{RR}$ activity, direct spectroscopic evidence of the complex interplay between EDL structure and reaction kinetics has remained elusive. Here, we introduce Dynamic Response Spectroscopy (DRS), a novel approach that isolates spectroscopic signatures of key physicochemical features of the EDL, including the compact (interfacial) layer and the diffuse double layer based on their time‐variance profiles. By analyzing multi‐dimensional time‐variance within a matrix of time‐resolved infrared spectral data recorded during sequential potential steps, we reveal that EDL equilibration is not continuous but involves discrete restructuring events. We provide spectroscopic evidence that these sudden EDL reorganizations correlate with the rapid adsorption and conversion of ${\mathrm{CO}}_{2}$ to CO. Furthermore, we show that saturation of aqueous NaHCO_3_ electrolytes with ${\mathrm{CO}}_{2}$, as opposed to Ar, induces more frequent and pronounced water reorientation in the diffuse double layer, characterized by less ice‐like ordering and increased randomness. These findings provide novel insights into the dynamic nature of the EDL and its synergistic role in electrocatalysis, establishing a paradigm to better understand, and thus optimize, electrochemical systems.

## Graphical abstract

Different regions of the electrode/electrolyte interface, such as the interfacial layer and diffuse double layer, are spectroscopically discerned by analyzing multi‐dimensional time‐variance within a matrix of time‐resolved infrared spectra. In this way, discrete events relating to reorientation of water molecules in the double layer are elucidated, which correlate with the rapid adsorption and conversion of ${\mathrm{CO}}_{2}$ to CO.

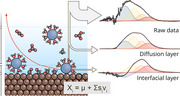

## Introduction

**Figure 1:**
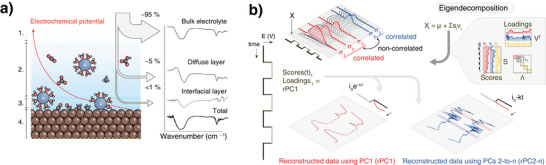
Schematic representation of the mixture analysis problem and our approach. a) The potential profile in studying the electrified solid‐liquid interface, along with representative FTIR spectra and their corresponding weight contributions at: 1. bulk electrolyte, 2. diffusion layer, 3. interfacial layer, 4. catalyst. b) Overview of the application of the data analysis approach. Through eigendecomposition of the covariance matrix of the (mean‐centered) dataset, the (linear) time variance response to an applied perturbation can be used to discriminate and spectroscopically resolve the different time‐dependent processes. This allows reconstruction of the data by multiplying the scaled loadings with the time‐dependent scores matrix (Scores(t)), using only selected principal components.

Electrochemical interconversion processes will likely play an increasingly important role in a more sustainable society.^[^ ^1^, ^2^ ^]^ An example is the electrocatalytic carbon dioxide reduction reaction (${\mathrm{CO}}_{2}\mathrm{RR}$). A key feature of electrified solid–liquid interfaces such as those at which ${\mathrm{CO}}_{2}\mathrm{RR}$ occurs is the electric double layer (EDL), which drives all relevant adsorption and conversion processes.^[^ ^3^, ^4^, ^5^ ^]^ The classical Gouy–Chapman‐Stern model describes the existence of two primary regions in the EDL: the Stern, or compact layer, closest to the electrode surface, where ions are specifically adsorbed; and the Gouy–Chapman, or diffuse double layer, where particle densities become more distributed farther into the electrolyte (Figure 1a).^[^ ^6^, ^7^, ^8^ ^]^ The microstructure of the EDL, according to this theory, arises from the thermodynamic equilibration of electrostatic properties of the electrode surface, and of the electrolyte, such as ion concentration and size.^[^ ^4^, ^5^, ^9^ ^]^ However, the former is in constant flux during catalytic turnover,^[^ ^10^, ^11^, ^12^ ^]^ and it was shown in recent accounts that different metastable states can be stabilized for extended periods in a single system.^[^ ^13^ ^]^ These two recent examples underscore the persistent enigma surrounding the EDL, despite its pivotal role in electrochemical applications. Remarkably, no existing model or theory can reliably predict the capacitance or microstructure of the EDL, even in the case of relatively simple single crystal facet systems.^[^ ^14^ ^]^ Accurately predicting the influence of the EDL on electrocatalytic activity, in turn, is thus even farther from our current capabilities, particularly for the complex systems more pertinent to real‐world applications, which often involve features such as porosity, high ionic strength electrolytes, and surface polycrystallinity. Despite these challenges, numerous studies have empirically linked ${\mathrm{CO}}_{2}\mathrm{RR}$ activity and selectivity to the properties of the EDL. For instance, EDL‐induced local electric fields have been proposed to facilitate ${\mathrm{CO}}_{2}$ adsorption, a crucial reaction step hindered by the inherently low solubility of ${\mathrm{CO}}_{2}$ in aqueous electrolytes.^[^ ^15^ ^]^ Hou et al. recently discussed that cations with weakly bound hydration shells, such as Cs^+^, form more compact and rigid double layers compared to those with strongly bound hydration shells, like Li^+^.^[^ ^13^ ^]^ This increased rigidity adversely affected the CO reduction reaction, a pivotal step in ${\mathrm{CO}}_{2}\mathrm{RR}$, by limiting the mobility of reactants and ions. Additionally, a combination of ab initio molecular dynamics (AIMD) simulations and Raman spectroscopy was used to demonstrate that varying Na^+^ concentrations can rearrange water structures, thereby enhancing interfacial electron transfer.^[^ ^16^ ^]^ Moreover, thinner and denser EDLs have been shown to impede proton transport to the cathode, suppressing the competing hydrogen evolution reaction (HER) and improving ${\mathrm{CO}}_{2}\mathrm{RR}$ selectivity.^[^ ^17^ ^]^ These findings highlight the critical role of the EDL in influencing reaction activity and underscore the need for a deeper understanding of its behavior. This knowledge, coupled with strategies to improve ${\mathrm{CO}}_{2}$ availability at the interface, such as GDEs^[^ ^18^ ^]^ or direct carbonate reduction,^[^ ^19^, ^20^ ^]^ is essential for optimizing ${\mathrm{CO}}_{2}\mathrm{RR}$ selectivity and efficiency.

There are a few methods available with which features of the EDL can be studied or distinguished. An example is Electrochemical Impedance Spectroscopy (EIS) where time‐dependent properties of a system can be distinguished from varied frequency impedance measurements via transfer function models. This is possible because different parts of the EDL have, or may be discussed as separate because of, distinct capacitance and resistance properties. For example, the compact or interfacial layer, with its high capacitance due to the small distance between charges and low resistance near the electrode, can be distinguishedby its fast, linear response to an applied electrical stimulus, governed by a low RC time constant.^[^ ^21^ ^]^ In contrast, the diffuse double layer and concentration gradients towards the bulk, the latter of which is governed by Nernstian diffusion and is not part of the EDL, typically display slower responses of exponential and 1/$\sqrt{t}$ nature, respectively.^[^ ^22^, ^23^ ^]^ However, techniques such as EIS and related methods leveraging frequency‐time domain conversion^[^ ^24^ ^]^ inherently depend on assumptions of predictable, continuous, and reversible system responses. As a result, they fail to capture nonlinear dynamic phenomena that may deviate from these assumptions, such as abrupt or exceedingly slow phase or morphological transformations during activity, rapid potential redistribution, or ion and product redistribution following product release.^[^ ^10^, ^25^ ^]^ Moreover, these methods lack the capability to provide chemical speciation, or direct insights into the reaction intermediates or the microstructure of the EDL.^[^ ^26^ ^]^ Techniques that have the capability to overcome these limitations, such as vibrational spectroscopy, face their own challenges, including noisy and convoluted signals that are often obscured by contributions from the bulk electrolyte, as illustrated in Figure 1a.

In this work, we introduce Dynamic Response Spectroscopy (DRS), a novel approach that overcomes the continuity or reversibility constraint of methods like EIS, but also allows us to speciate (i.e. obtain chemical information on the key physicochemical features of the EDL) via vibrational spectroscopy, including the compact and diffuse double layer. Simply put, the method explicitly avoids imposing the assumption of Linear Time Invariance (LTI) on collected spectral data and is aimed at separating the parts of spectra that have a shared, specific time‐response to an applied potential pulse. Then, using the time‐response profiles–be they linear, exponential, or more complex–it allows us to allocate the spectral contribution into to differently time‐variant fractions. We focus on disentangling EDL contributions in ${\mathrm{CO}}_{2}\mathrm{RR}$ over Cu as it is the only monometallic catalyst producing C_2+_ products with appreciable Faradaic efficiency,^[^ ^27^, ^28^, ^29^, ^30^ ^]^ and study the reaction by applying sequential potential steps while capturing attenuated total reflectance infrared absorption (ATR‐SEIRA) spectra. By combining the potential step experimental approach with eigendecomposition to obtain time‐patterns and spectral components, we avoid assumptions of LTI and excessive time‐averaging, as schematically illustrated in Figure 1b. Through matrix reconstruction by grouping of the obtained time‐profiles we thus obtain the spectra associated with the distinct constituents of the EDL. We validate DRS through complementary methods such as confocal Raman micro‐spectroscopy and Differential Electrochemical Mass Spectrometry (DEMS), alongside comprehensive numerical simulations. We apply DRS across a range of conditions, including varying electrolyte concentrations (0.002 to 1 M), solvent ($H_{2}O$ and D_2_O), potential windows (−0.05 to −1.1 V_RHE_), and both ${\mathrm{CO}}_{2}$‐ and Ar‐saturated electrolytes, providing insight into the dynamic nature and role of the EDL during ${\mathrm{CO}}_{2}\mathrm{RR}$ over copper. Our results reveal the formation of rigid, surface‐adjacent structures within the EDL, with increasing significance at higher electrolyte concentrations. We also observe abrupt changes in the EDL structure, indicative of non‐continuous double layer restructuring which correlate strongly with ${\mathrm{CO}}_{2}\mathrm{RR}$ performance.

## Results and Discussion

### Speciation of the Double Layer

As mentioned, characterizing ${\mathrm{CO}}_{2}\mathrm{RR}$ in aqueous media with vibrational spectroscopy is challenging due to the low solubility of ${\mathrm{CO}}_{2}$ in water (approximately 0.05 M)^[^ ^31^, ^32^, ^33^ ^]^ and the strong infrared absorption by water, which lowers signal‐to‐noise ratios and complicates the detection of reaction intermediates.^[^ ^34^, ^35^, ^36^, ^37^ ^]^ This often necessitates the use of costly isotopically labeled reagents^[^ ^38^, ^39^, ^40^ ^]^ to identify relevant infrared signals. Complex nonlinear processes like Nernstian diffusion and multilayer capacitance, and their impact on reaction kinetics, further complicate spectroscopic studies. Thus, studying them requires innovative photon‐based approaches able to disentangle convoluted system events. To address these challenges, we propose to identify different double layer components (specifically, separating interfacial processes from the diffuse double layer) by speciating them spectrally using matrix decomposition. We employ ATR‐SEIRAS, where an evanescent wave penetrates a few micrometers into the interface, deep enough to extend beyond the EDL, and capture the concentration gradient region.^[^ ^41^, ^42^ ^]^ Sequential Cottrell experiments were then performed,^[^ ^4^, ^43^, ^44^ ^]^ where the electrode is held at an inert potential before switching to a reactive potential, while collecting time‐resolved spectroscopic data. Unless stated otherwise, the experimental results shown in this paper correspond to the alternation of applied potential pulses at −0.4 and −0.8 V_RHE_, held for 100 s at each potential during five cycles while recording Fourier Transform Infrared (FTIR) spectra. We fabricated microgrooved Si internal reflection elements (IREs) based on literature designs^[^ ^42^, ^45^ ^]^ (Figure S2), coated with a 30 nm polycrystalline copper layer deposited via e‐beam evaporation. Four‐point measurements were performed on different samples, yielding resistivities of 9.33 · 10^−8^ ± 0.01· 10^−8^ Ωm, thus confirming the high conductivity of the films. A home‐built, airtight spectro‐electrochemical cell (Figure S4a) was filled with a 0.2 M $\mathrm{NaH}{\mathrm{CO}}_{3}$ solution, pre‐cleaned to remove metal impurities,^[^ ^46^, ^47^ ^]^ and bubbled with ${\mathrm{CO}}_{2}$ or Ar. Detailed experimental procedures are available in Section S1 of the Supporting Information.

The spectra for both the ${\mathrm{CO}}_{2}$ and Ar‐saturated electrolytes were dominated by water absorption bands^[^ ^48^, ^49^, ^50^ ^]^ (Figure S17) when both $H_{2}O$, and D_2_O were used. A faint peak at 2075 cm^−1^, attributed to linearly adsorbed CO from ${\mathrm{CO}}_{2}\mathrm{RR}$,^[^ ^35^, ^37^, ^51^, ^52^, ^53^ ^]^ was barely discernible. To identify and group spectral features with distinct time‐variant behavior into principal components (PCs), we applied eigendecomposition to the covariance matrix of our data, as is standard in principal component analysis (PCA), proper orthogonal decomposition (POD), and singular value decomposition (SVD) (see Equations S6 and S7). Figure 1b provides a schematic overview of this process, while Section S2.3 of the Supporting Information offers a detailed analysis, including a comprehensive discussion of why we selected this method over other decomposition techniques, supported by numerical simulations.

**Figure 2:**
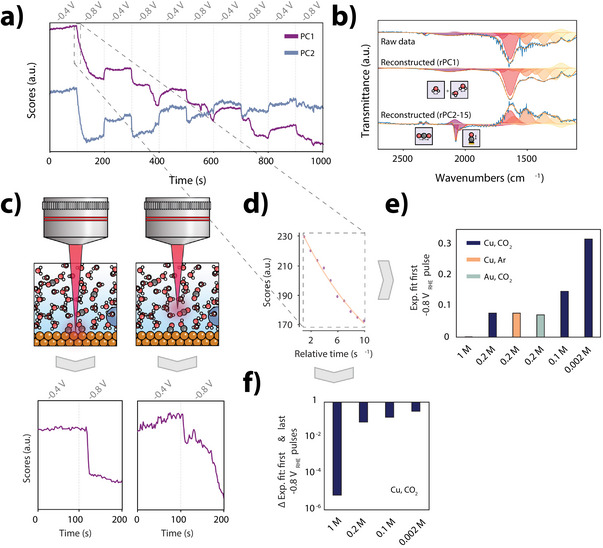
a) Time evolution of PC1 and PC2 scores from eigendecomposition of ATR‐SEIRAS spectra during pulses at −0.4 and −0.8 V_RHE_ using ${\mathrm{CO}}_{2}$‐saturated $\mathrm{NaH}{\mathrm{CO}}_{3}$ 0.2 M. b) FTIR spectra (2500–1100 cm^−1^) from the raw dataset (top), and reconstructions using PC1 (middle) and PCs 2‐15 (bottom). c) Raman micro‐spectroscopy illustration with laser focused on the electrode surface (left) or focused 11.7 μm above it (right), showing below, the score evolution for changes in the ν(OH) region switching from −0.4 to −0.8 V_RHE_. d) Example fit of scores, as a diffusion coefficient proxy. e) Exponential factor for various conditions: 1, 0.2, 0.1, and 0.002 M in ${\mathrm{CO}}_{2}$, and 0.2 M in Ar on Cu, and on Au in  ${\mathrm{CO}}_{2}$. f) Change in fit exponent value from first to last −0.8 V_RHE_ pulse (at 100 vs. 900 s), indicating capacity discharge.

By examining these PCs, we can further separate the captured spectral elements based on 1) their individual time‐dependent profiles, and 2) the specific spectral features associated with such time‐variance. The former is captured in the “scores” matrix, while the spectral features themselves are represented by the “loadings” matrix (see Section S2.3 of the Supporting Information for further information). The scores of the PCs (Figures 2a and S19–S21) revealed distinct groups of time‐resolved behaviors for all conditions applied (different concentrations of electrolyte, different potential windows, using Au instead of Cu, vide infra). That is, PC1 always exhibited a continued exponential response following the applied pulses at −0.8 V_RHE_, while PCs 2 to 15 showed a more linear, step‐wise pattern, or a square wave response that resembled the original potential modulation (Figure S19). The distinct time responses are the first identifiable distinction between the interfacial and diffuse double layers – linear and exponential, respectively – which allow us to recognize them under these conditions as separate entities, at least from a dynamical perspective. By combining the scores and eigenspectra of PC1, as outlined in Equation S8, we can reconstruct spectra that are grouped based on their time‐variant nature (Figure 2b). These reconstructed spectra referred to as rPC1 (reconstructed data using PC1), show significantly reduced noise compared to the raw data, and are largely dominated by water signals. The exponential time‐variance of rPC1 could suggest that these spectra correspond to the diffusion layer. In contrast, spectra reconstructed from the more linear responses seen in PCs 2 to 15 (rPC2‐15) reveal adsorbed species associated with ${\mathrm{CO}}_{2}\mathrm{RR}$, such as distinct peaks at 2078 cm^−1^ and 1541 cm^−1^, corresponding to *CO (where * denotes adsorbed) and *HCOO, respectively.^[^ ^51^, ^52^, ^53^, ^54^, ^55^, ^56^ ^]^ While theoretically the individual (ultra)fast contributions encountered in this group of PCs (rPC2‐15) could be further speciated by increasing the time‐resolution of the experimental set‐up, we opt here to group them together, as the employed time resolution of 1.1 s does not allow to distinguish their individual dynamics. Furthermore, the main focus of this work is the dynamic interplay within the EDL, between the diffuse double layer and interfacial layer, rather than within the latter. Interfacial processes are typically observed on very short timescales immediately after a potential switch–see around 200 s in Figure 2a, for example. However, slower processes can also occur at the interface. These can arise from feedback loops that alter local pH or reactant concentrations,^[^ ^57^ ^]^ leading to a more gradual temporal evolution especially during system equilibration. For instance, after the initial rapid response to the first potential switch at 100 s, a slower change is observed over the following few seconds. Tables S1– S3 summarize the observed IR peaks and their assignments. The linear nature of the time‐response of this group of features, combined with the nature of the identified species, suggest contributions from the species closest to and on the electrode surface; the interfacial layer. This is visualized in the time‐series evolution of the spectra after reconstruction (Figure S31). Gaussian curve fitting was employed to extract additional spectral insights, with peak centers selected based on their presence in the eigenspectra (Figure S22 and summarized in Table S2). Further details on the fitting process, along with the complete fits, are provided in Section S1.1.7. This approach not only allowed us to speciate different time‐variant behavior, but also to accurately capture subtle features in the spectra that would otherwise be difficult to resolve and assign with confidence, as most clearly seen when comparing *CO in the raw data to its signal in rPC2‐15 (Figure 2b).

To further validate the time‐resolved speciation of different electrochemical layers, we performed complementary Raman micro‐spectroscopy (Figure 2c), focusing the beam at different distances, i.e., at the surface of the electrode and slightly above it. The same treatment of the Raman data (similar to the ATR‐SEIRAS data discussed above) confirms that exponential decays are mainly found above the electrode, while notably sharper transitions resembling square waves or linear responses were observed mainly when focused onto the electrode. Raman spectra collected near the electrode also showed additional abrupt time‐variant features that were seemingly unrelated to the application of potential pulses (as also observed in the scores matrix for the ATR‐SEIRAS spectra in Figure 2a). These will be discussed extensively in the last section of this work.

Control experiments with varying electrolyte concentrations (0.002, 0.1, 0.2, and 1 M) and alternative metal coatings (Au instead of Cu) further corroborated the suitability of the approach to distinguish properties of the diffusion and interfacial layers. That is, despite the dimensionless nature of the scores, which complicates direct calculation of a diffusion coefficient D, fitting the initial 10 s post‐potential switch (Figure 2d) should be correlated with the diffusion coefficient if the time‐variant nature of the scores is in fact a real feature of the EDL. Figure 2e reveals that indeed, higher electrolyte concentrations decreased the exponential factor, signaling lower diffusion rates. Similar concentrations of electrolyte with varying –${\mathrm{CO}}_{2}$ saturation or metal electrode yield the same value, consistent with expectations from Stokes–Einstein relationships.^[^ ^58^ ^]^ Differences in this proxy for D between the first and last pulses highlighted the reversibility of charge‐discharge behavior, which is influenced by electrolyte concentration (Figure 2f).^[^ ^17^, ^59^, ^60^, ^61^ ^]^ Here, lower concentrations resulted in higher differences between the first and last pulses, indicating reduced ion availability and less efficient cycling at lower concentrations, as would be expected.

These control experiments confirm that, despite making no direct correlation with the resulting current and without imposing any a priori assumptions about the system's response, we can recover century‐old^[^ ^43^, ^62^ ^]^ time‐variance trends observed in potential‐step experiments, allowing us to effectively separate – and now speciate – distinct contributions of double layer features. Leveraging this separation, we will now further corroborate the method, while gaining more detailed insights into the spectroscopic data. By distinguishing between linear, exponential, and more complex groups of time‐variant trends, we can begin to unravel how these different fractions of the double layer and beyond contribute to system behavior. First, we explore the structure of water within the double layer, a critical aspect influencing ion transport and reaction efficiency. Next, we examine the role of reaction intermediates, shedding light on their formation and behavior within the layers. Finally, we analyze the dynamic interactions that govern the overall system performance. This stepwise approach allows us to build up a comprehensive understanding of how each component contributes and is interrelated with the overall electrochemical process.

### The Structure of Water in the Double Layer

**Figure 3:**
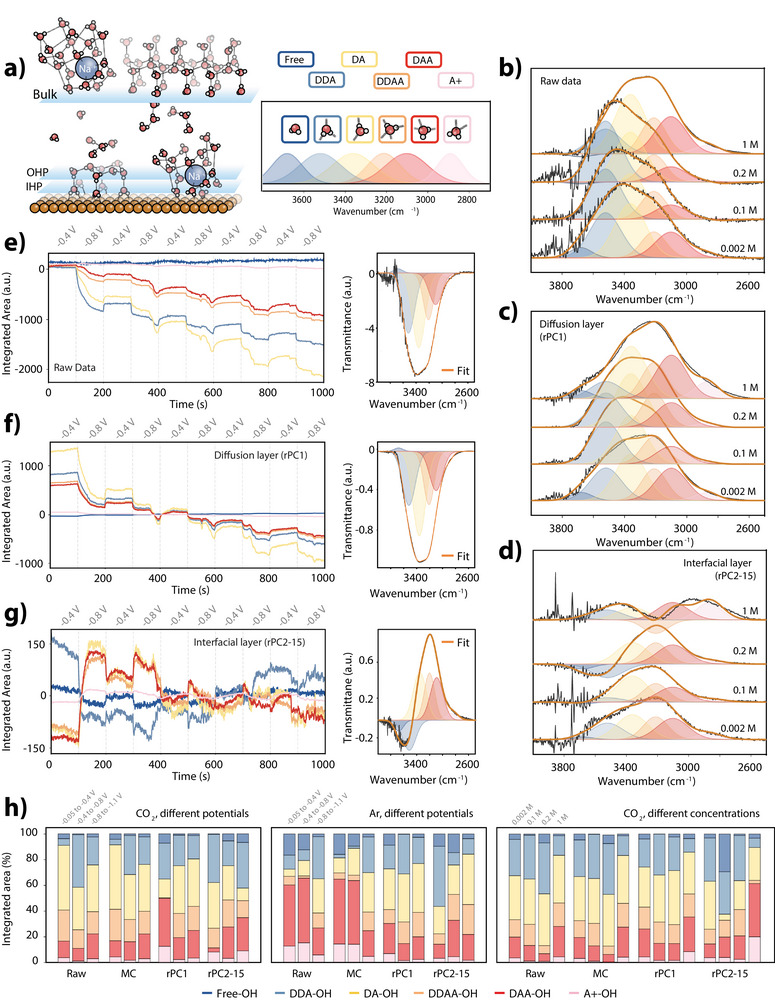
Overview of curve fitting in the water region of operando ATR‐SEIRAS. a) Illustration of water structures in the EDL and their corresponding FTIR peaks at 3680 (free‐ν(OH)), 3520 (DDA‐ν(OH)), 3360 (DA‐ν(OH)), 3210 (DDAA‐ν(OH)), 3100 (DAA‐ν(OH)), and 2870 (A^+^‐ν(OH)) cm^−1^. b)–d) Curve‐fitted FTIR spectra in the ν(OH) region for 1 M, 0.2 M, 0.1 M, and 0.002 M ${\mathrm{CO}}_{2}$‐saturated $\mathrm{NaH}{\mathrm{CO}}_{3}$ at −0.8 V_RHE_ for b) raw data, c) diffusion layer (rPC1), and d) interfacial layer (rPC2‐15). e‐ge Evolution of peak areas during alternating potentials (−0.4 to −0.8 V_RHE_) in ${\mathrm{CO}}_{2}$‐saturated $\mathrm{NaH}{\mathrm{CO}}_{3}$ 0.2 M from e) the raw data, f) diffusion layer (PC1), and g) interfacial layer (PCs 2‐15). h) Normalized fraction of integrated areas for fitted peaks during potential alternations in $\mathrm{NaH}{\mathrm{CO}}_{3}$ 0.2 M saturated with ${\mathrm{CO}}_{2}$ (left) and Ar (center), and in ${\mathrm{CO}}_{2}$‐saturated solutions of different concentrations (right); 0.002, 0.1, 0.2, and 1 M.

The broad ν(OH) band around 3400 cm^−1^ is believed to consist of several distinct hydrogen‐bonding configurations existing in a dynamic, fluctuating network. These configurations differ in the number of hydrogen bonds donated or accepted and in their symmetry. Although we note that assigning precise vibrational modes to each hydrogen‐bonding motif oversimplifies the true complexity of the water structure, experimental, and theoretical investigations consistently reveal two trends: in bulk liquid water, more symmetrical networks – typically characterized by DDAA (double–donor, double–acceptor) and DA (single–donor, single–acceptor) configurations – prevail. In contrast, disruptions of such bulk behavior, for example, in interfacial environments such as the EDL, promote asymmetrical arrangements (e.g., DAA and DDA) due to the influence of the interface or the presence of cations (Figure 3a).^[^ ^63^, ^64^, ^65^, ^66^, ^67^, ^68^, ^69^, ^70^, ^71^ ^]^ Although the schematic in Figure 3a illustrates these water configurations in an idealized manner, all such motifs are distributed throughout the electrochemical cell, albeit in differing proportions between regions, such as the bulk electrolyte versus the EDL. The reorientation of water molecules upon electrification has been well‐documented with vibrational spectroscopy. For example, studies by Osawa and colleagues have shown that water reorients at metal interfaces and that the resulting structures are disrupted upon cation approach.^[^ ^72^, ^73^, ^74^, ^75^ ^]^ This dynamic behavior of water at the interface – along with the impact of cation hydration – modulates both ion transport and the stabilization of reaction intermediates, and is thus of great interest to further examine.

In our eigenspectra (see Figure S22), distinct peaks and shoulders within the νOH region support the existence of several predominant hydrogen‐bond motifs. In accordance with literature, we assign the shoulder at approximately 3520 cm^−1^ to DDA (double–donor, single–acceptor) species, the peak near 3360 cm^−1^ to DA species, the peak around 3210 cm^−1^ to DDAA species, and the peak at about 3100 cm^−1^ to DAA (single–donor, double–acceptor) species. Additional shoulders observed at 3680 cm^−1^ and 2870 cm^−1^ are attributed, respectively, to OH groups with a weaker hydrogen‐bonding environment (often described as free OH, e.g., hydroxyl groups) and to poly‐acceptor (A^+^–ν(OH)) species.^[^ ^69^, ^70^, ^71^ ^]^

To explore the evolution of these water species during the ${\mathrm{CO}}_{2}\mathrm{RR}$, Figure 3b shows ATR‐SEIRA spectra of ν(OH) across different electrolyte concentrations (1, 0.2, 0.1, and 0.002 M) at −0.8 V_RHE_. While DDAA‐ν(OH) and DA‐ν(OH) should dominate liquid (deionized) water,^[^ ^69^, ^76^, ^77^, ^78^ ^]^ we see the predominant feature at 3450 cm^−1^ for 0.2, 0.1, and 0.002 M, indicating the prevalence of more asymmetrically bound, DDA‐ν(OH). This shift to higher wavenumbers suggests alkali cation coordination^[^ ^71^ ^]^ and/or more tightly packed water at the interface, with DDA‐ν(OH) intensity increasing with salt concentration, as expected. At 1 M, a significant redshift to 3200 cm^−1^ is observed, which can be indicative of the formation of larger water clusters stabilized by the higher sodium ion concentration.^[^ ^79^ ^]^ This redshift is particularly pronounced in the diffusion layer (Figure 3c), where the DAA‐ν(OH) peak emerges more sharply, while DDAA‐ν(OH) diminishes. At higher concentrations, the broadening of the ν(OH) band indicates an increase in asymmetrical species and free OH and A^+^‐ν(OH) species, likely associated with Na($H_{2}O$)_X_ clusters.^[^ ^71^ ^]^ In the interfacial layer (Figure 3d), the ν(OH) band consistently shifts towards lower wavenumbers cm^−1^ across all concentrations, suggesting more tightly packed water, for example, by alkali cation accumulation near the electrode surface during cathodization. As concentration of the electrolyte increases to 1 M, the band splits into two, resembling the behavior of confined water molecules described by Shin et al.,^[^ ^80^ ^]^ indicating structural confinement and the formation of structured, densely packed, or ice‐like water near the electrode surface. This was further supported by Dodin and colleagues,^[^ ^59^ ^]^ who noted the absence of the peak at 3200 cm^−1^ during sodium carbonate ion complex formation near the electrode surface.

The dynamic evolution of the water species under the applied electrochemical conditions is summarized in Figure 3e, which shows the time evolution of the areas of Gaussian fits to the raw dataset using 0.2M $\mathrm{NaH}{\mathrm{CO}}_{3}$. We note that the data is represented in transmittance, thus more negative signals correspond to increased concentrations. The initial pulse at −0.4 V_RHE_ shows minimal change, while a subsequent pulse at −0.8 V_RHE_ results in exponential increases in all water species, particularly in more strongly hydrogen‐bonded structures (DDA‐ν(OH), DA‐ν(OH), DDAA‐ν(OH), DAA‐ν(OH)) compared to hydroxyl/free and A^+^‐ν(OH) species. This suggests that the local electric field drives a rearrangement of structures in the EDL forming more ordered or closely packed structures. Upon returning to −0.4 V_RHE_, these changes partially reverse. The time‐resolved trends of the mean centered data in Figure S32 align with expectations that bulk water primarily consists of DDAA‐ν(OH) and DA‐ν(OH),^[^ ^69^, ^76^, ^77^ ^]^ with barely any response to the applied potential pulses. Nevertheless, these trends were captured (and removed following the mean‐centering pre‐processing step prior to eigendecomposition) due to the high penetration depth of the evanescent wave (μm), in comparison to the interfacial and diffusion layers, which are confined to Å and nm scale, respectively.^[^ ^3^, ^17^, ^81^ ^]^ The time‐resolved intensity plots of the areas of the Gaussian fits for the diffusion layer (Figure 3f) show exponential trends similar to the raw data, with the highest variations recorded for DDA‐ν(OH) and DA‐ν(OH), followed by DDAA‐ν(OH) and DAA‐ν(OH). Strange discontinuities can be seen in the time‐resolved data, which will be discussed in detail in the section “Double Layer Restructuring” The features associated with water species within the linear time response group (rPC2‐15, Figure 3g) should include distinct features of specifically oriented water molecules, for example, in the hydration shell of cations. This is reflected in the slightly lower fit quality of this group of spectra, yet an increase in free‐ν(OH) and A^+^‐ν(OH) is again clearly observed. These trends align with previous DFT calculations,^[^ ^71^ ^]^ where vibrational signals below 3000 cm^−1^ emerge upon alkali salt coordination.

Finally, Figure 3h summarizes the normalized fractions of water from the Gaussian areas, reflecting the variability of fitted peaks at −0.8 V_RHE_ from their original (lower potential) state. The mean‐center contribution (bulk electrolyte) shows the least deviation consistently. The effect of ${\mathrm{CO}}_{2}$ on water structure can be noted by comparing Ar‐ and CO_2_‐saturated solutions in 0.2 M NaHCO_3_. Comparing the left (CO_2_) and middle (Ar) panels, the ν(OH) band is redshifted in Ar‐saturated experiments at lower overpotentials. However, at more reducing potentials (−1.1 V_RHE_) the structures in Ar‐ and CO_2_‐saturated experiments converge to a more similar distribution of water types. This shows that the presence of ${\mathrm{CO}}_{2}$ inhibits the close packing of water, which is prevalent in Ar‐saturated data at lower potentials. A reasonable explanation for this is size asymmetry of the carbonate‐Na complex, disrupting periodic packing of water. Recent studies suggest that this size difference can lead to local collapse of the double layer structure.^[^ ^59^, ^82^ ^]^ Additionally, as electrolyte concentration increases, the interfacial layer becomes progressively “packed” with more ice‐like and A^+^ species compared to the diffusion layer, particularly in the 1 M concentration regime. This increasing confinement suggests a growing dominance of tightly bound water molecules with rising concentration, contributing to distinct structural reorganization in the interfacial layer and enhancing the interfacial packing. Further supporting this, modulating the potential above and below the potential of zero free charge (pzfc) − specifically from −0.05 V to −0.4 V_RHE_ ‐− yields the highest relative variance in the fraction of confined water within the interfacial layer. This observation is elaborated in Section S4.3 of the Supporting Information, where we provide a detailed analysis of how the pzfc was determined and how its modulation impacts the interfacial water structure. The results reinforce the idea that potential modulation around the pzfc profoundly affects key interfacial water properties. Such transitions include hydrogen‐bonding networks and molecular orientation, which are critical for ion transport and electrochemical reactions,^[^ ^9^, ^72^, ^73^ ^]^ and show that the presence of dissolved ${\mathrm{CO}}_{2}$ increases the degree to which the inner most layer of water reorients, likely influenced to a certain degree by its adsorption.

### Reaction Intermediates

**Figure 4:**
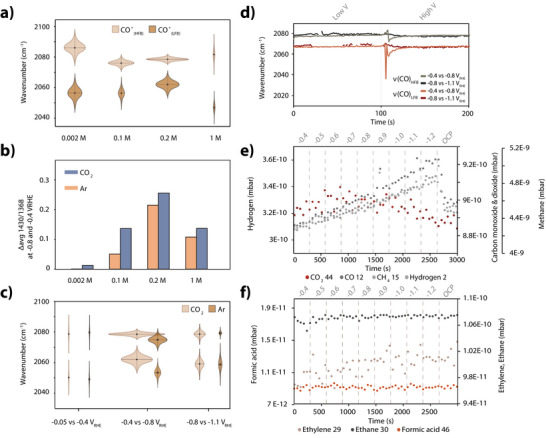
a) Gaussian peak center positions (centroid), standard deviations (height), and intensities (width) comparing the average positions of *CO_HFB_ and *CO_LFB_ during the course of the experiment in 0.002, 0.1, 0.2, and 1M $\mathrm{NaH}{\mathrm{CO}}_{3}$ while alternating the potentials between −0.4 and −0.8 V_RHE_. b) Local pH change due to −0.4 to −0.8 V_RHE_ pulses calculated by the average ratio of the areas of the Gaussian fits at 1430/1368 cm^−1^ at high and low potential in rPC2‐15. c) Gaussian peak center positions (centroid), standard deviations (height), and intensities (width) comparing the average positions of *CO_HFB_ and *CO_LFB_ during the course of the experiment in 0.2M $\mathrm{NaH}{\mathrm{CO}}_{3}$ while alternating the potentials between −0.05 and −0.4 V_RHE_, −0.4 and −0.8 V_RHE_, and −0.8 and −1.1 V_RHE_. d) Evolution of the Gaussian peak center positions for ν (CO)_HFB_ and ν(CO)_LFB_ during potential switches (−0.4 to −0.8 V_RHE_, −0.8 to −1.1 V_RHE_) in 0.2 M NaHCO_3_ bubbled with ${\mathrm{CO}}_{2}$. e, f) DEMS results during staircase electrolysis (−0.4 to −1.2 V_RHE_) for e) ${\mathrm{CO}}_{2}$, CO, ${\mathrm{CH}}_{4}$, $H_{2}$ (*m/z* 44, 12, 15, 2), correcting *m/z* 12 for ${\mathrm{CO}}_{2}$ interference, and f) ethylene, ethane, formic acid (*m/z* 29, 30, 46).

Now that we have examined the structural features of the water layers, it is of interest to discuss how these findings correlate with reaction intermediates and catalytic activity. Figure 4a presents the average positions (centroids) of the high frequency band (HFB) and low frequency band (LFB) *CO_HFB_ and *CO_LFB_ peaks, along with their standard deviations (violin height) and peak area intensities (violin width). Notably, the degree of water confinement in the interfacial layer appears to be positively correlated with the intensity of the *CO peak in the FTIR spectra – a known proxy for catalytic activity – up to a concentration of 0.2 M.^[^ ^17^ ^]^ Interestingly, at 1 M, the *CO peak is nearly absent, indicating a significant reduction in catalytic activity. This decrease in activity is attributed to EDL saturation, as evidenced by the reduced diffusion coefficients shown in Figure 2 and the decline in ${\mathrm{CO}}_{2}$_(aq)_ concentration presented in Figure S34.

This trend is further supported by the pH changes observed during active versus non‐active potentials. Figure 4b shows the difference between the carbonate (1430 cm^−1^) and bicarbonate (1368 cm^−1^) peak ratios at −0.4 V and −0.8 V_RHE_, across varying electrolyte concentrations.^[^ ^83^ ^]^ These pH differences provide indirect evidence of catalytic activity, as more significant pH changes are observed in ${\mathrm{CO}}_{2}$‐saturated solutions,^[^ ^84^ ^]^ where *CO formation is more prominent. The increase in local pH during ${\mathrm{CO}}_{2}\mathrm{RR}$ is attributed to the accumulation of hydroxide ions, which occurs as protons are consumed in the reaction process.^[^ ^85^ ^]^ In comparing rPC2‐15 across 0.002 M, 0.1 M, and 0.2 M electrolyte concentrations, we observe higher pH differences at higher electrolyte concentrations, emphasizing the role of cations as crucial components in the ${\mathrm{CO}}_{2}\mathrm{RR}$ mechanism.^[^ ^86^, ^87^, ^88^ ^]^ However, at 1 M, the pH differences are notably lower than at 0.2 M, suggesting perhaps that there is an optimum in the packing density of water molecules within the interfacial layer for activity. This is in line with recent literature showing excessive salt concentration can lead to local ${\mathrm{CO}}_{2}$ depletion, thus limiting reaction efficiency.^[^ ^17^ ^]^

Figure 4c summarizes the average *CO peak positions over the course of the experiments in both Ar‐ and ${\mathrm{CO}}_{2}$‐saturated electrolytes at varying potential windows (−0.05 vs. −0.4, −0.4 vs. −0.8, and −0.8 vs. −1.1 V_RHE_). Larger *CO peak shifts were observed between −0.4 and −0.8 V_RHE_, with minimal shifts between −0.05 and −0.4 V_RHE_. Shifts were higher in ${\mathrm{CO}}_{2}$‐saturated solutions compared to Ar as well, suggesting a direct correlation between ${\mathrm{CO}}_{2}$ concentration and the *CO peak center position, or Stark effect. This supports the hypothesis that increased ${\mathrm{CO}}_{2}$ concentrations enhance surface restructuring, leading to changes in catalyst morphology during reactions, as previously suggested.^[^ ^10^, ^11^ ^]^ Shifts in the peak center position of *CO_HFB_ and *CO_LFB_ (Figure 4d), from which Stark tuning rates can be calculated, are more intense during lower potential pulses. This may reflect saturation of the *CO dipole response at more negative potentials, reducing the system's responsiveness to further electric field modulation or to restructuring of the electrode surface.^[^ ^89^, ^90^ ^]^

**Figure 5:**
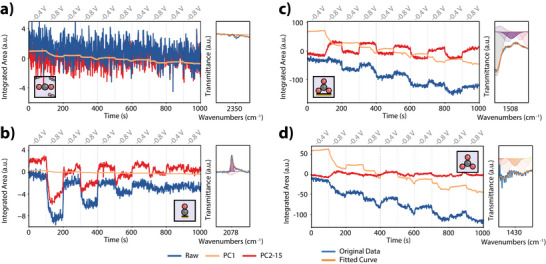
Integrated area over time of Gaussian peaks fitted in the raw data matrix during alternating pulses at −0.4 and −0.8 V_RHE_, and the reconstructed data matrices from PC1 and PCs 2–15, at a) 2350 cm^−1^ (${\mathrm{CO}}_{2}$(aq)), b) 2078 cm^−1^ (*CO), c) 1508 cm^−1^ (*CO_3_^2‐^), and d) 1430 cm^−1^ (CO_3_^2‐^).

Before going into more detail with respect to mechanistic observations of our aqueous‐phase ${\mathrm{CO}}_{2}\mathrm{RR}$ experiments, we note that they are likely to be − to a certain extent – system‐specific, as previous studies have shown that experimental parameters can strongly influence the reaction mechanism due to factors like ${\mathrm{CO}}_{2}$ mass transfer limitations which are difficult to avoid,^[^ ^91^ ^]^ or altered selectivity with varying pulse lengths.^[^ ^92^, ^93^ ^]^ To better understand ${\mathrm{CO}}_{2}\mathrm{RR}$ activity in our system, DEMS was performed using a custom‐built cell (schematic in S1.1.5) during stepped‐potential electrolysis from −0.4 to −1.2 V_RHE_, with 300 s intervals to allow sufficient product formation for detection. The results (Figure 4e,f) show increasing production of CO, ${\mathrm{CH}}_{4}$, and $H_{2}$ as the potential becomes more negative, along with a gradual increase in $C_{2}H_{4}$. To further examine the time‐variant behavior of key reaction intermediates, Gaussians at 2350 cm^−1^ (${\mathrm{CO}}_{2}$(aq)), 2078 cm^−1^ (*CO), 1508 cm^−1^ (*CO_3_^2−^), and 1430 cm^−1^ (CO_3_^2‐^) were fitted to modulated potential experiments at 0.2 M in ${\mathrm{CO}}_{2}$‐saturated electrolyte (Figure 5). Despite noise in the raw data, rPC1 analysis (Figure 5a) reveals a clear trend for ${\mathrm{CO}}_{2}$(aq) concentrations increasing in the diffuse double layer at −0.8 V_RHE_. This trend was absent in Ar‐saturated control experiments (Figure S33), suggesting that ${\mathrm{CO}}_{2}$ availability is limited by relatively slow equilibration with carbonate species.^[^ ^38^, ^52^, ^94^ ^]^ Interestingly, only noise can be observed in the time‐profile for rPC2‐15, which is reasonable if rapid (i.e., faster than our 1.1 s time resolution) ${\mathrm{CO}}_{2}$ conversion occurs upon reaching the interfacial layer, consistent with the low energy barriers reported for ${\mathrm{CO}}_{2}$ adsorption and subsequent conversion to *COOH and *CO.^[^ ^15^, ^95^, ^96^ ^]^ The rapid, linear response of *CO in rPC2‐15 to potential modulation (Figure 5b) underscores this rapid conversion, with decreasing concentrations at −0.4 V_RHE_ due to the weaker interfacial electric field as the potential approaches the pzfc.^[^ ^13^ ^]^ Carbonate species exhibited distinct behaviors: vibrations at 1508 and 1430 cm^−1^ (Figure 5c,d) correspond to free and adsorbed forms, respectively. Adsorbed carbonate, showing more distinct responses to the potential in rPC2‐15, competes with ${\mathrm{CO}}_{2}$ for active sites but is not readily converted to CO.^[^ ^97^ ^]^ This is in line with the stability of the observed response to potential throughout the experiment, in contrast to *CO intensity which decreased in later pulses, possibly due to the local depletion of ${\mathrm{CO}}_{2}$(aq). It is also possible that surface poisoning occurred by the accumulation of cationic species,^[^ ^98^ ^]^ which may lead to occlusion of the copper electrode by crystallization of accumulated, desolvated electrolyte species (see also Figure S45).^[^ ^99^, ^100^ ^]^

Additionally, some time‐variant profiles show abrupt time events, which were previously fleetingly noted, and which deserve additional discussion as covered in the following section.

### Double Layer Restructuring

**Figure 6:**
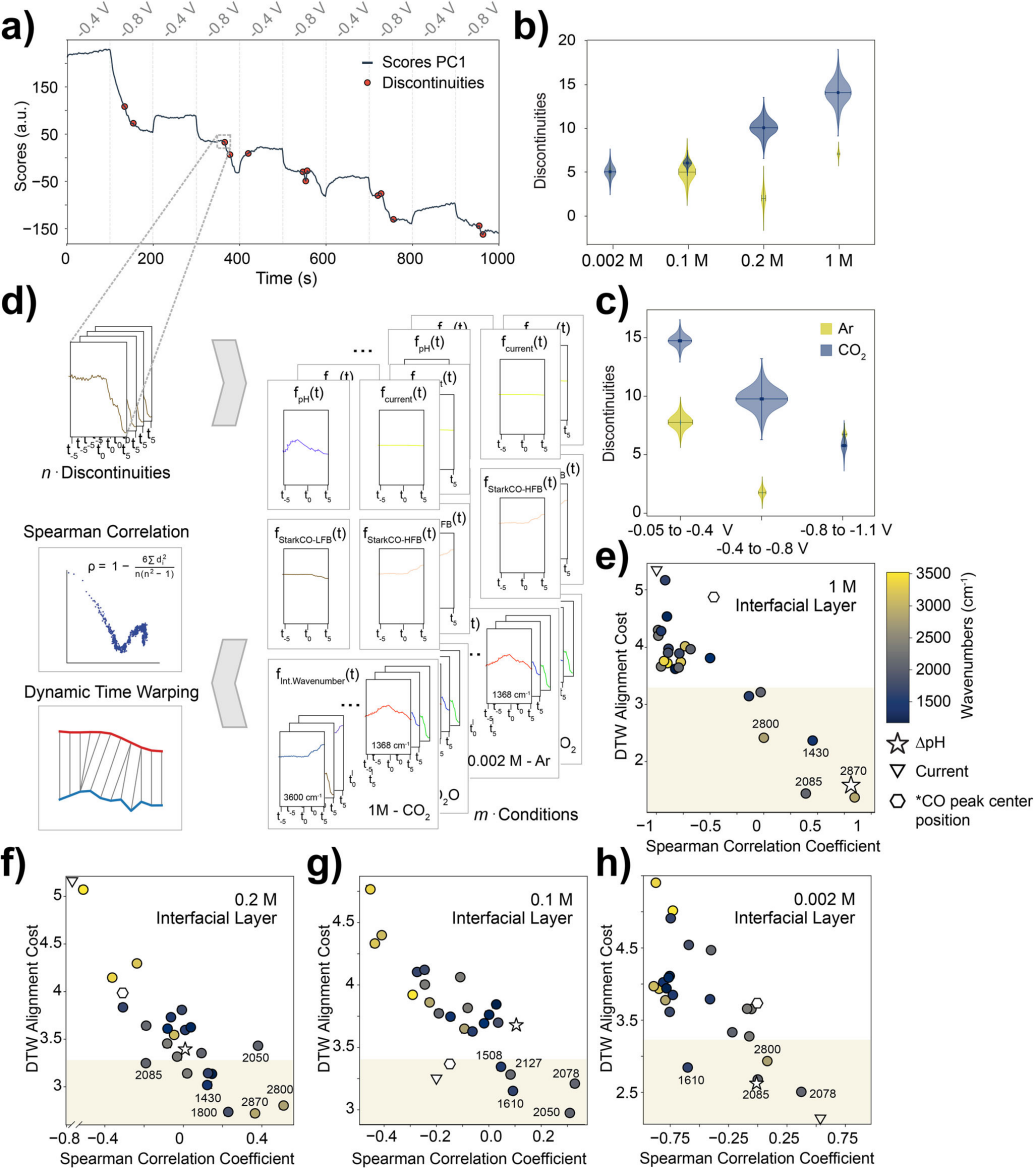
a) Detected discontinuities in PC1 scores over time from a standard experiment with a ${\mathrm{CO}}_{2}$‐saturated 0.2 M $\mathrm{NaH}{\mathrm{CO}}_{3}$ solution. b) Number of discontinuities (centroid) for ${\mathrm{CO}}_{2}$‐saturated $\mathrm{NaH}{\mathrm{CO}}_{3}$ solutions at 0.002, 0.1, 0.2, and 1 M under alternating electrochemical pulses at −0.4 and −0.8 V_RHE_. The height of the flared data points represents the average discontinuity magnitude, with widths indicating standard deviation. c) Number of discontinuities (centroid) for a 0.2 M $\mathrm{NaH}{\mathrm{CO}}_{3}$ solution under alternating potential windows: −0.05 to −0.4, −0.4 to −0.8, and −0.8 to −1.1 V_RHE_. d) Statistical analysis: for each discontinuity at t = 0, data points from t ±5.5 s were extracted for PC1, current, pH, CO peak center, and ATR‐SEIRAS peak areas, followed by Spearman correlation and DTW alignment cost analysis. e)–h) Correlation between DTW costs and Spearman coefficients for ${\mathrm{CO}}_{2}$‐saturated $\mathrm{NaH}{\mathrm{CO}}_{3}$ at 1 M (e), 0.2 M (f), 0.1 M (g), and 0.002 M (h) under alternating pulses at −0.4 and −0.8 V_RHE_. Circles represent correlations with ATR‐SEIRAS peak areas (color gradient), with stars, hexagons, and triangles denoting pH, CO peak center position, and overall measured current, respectively.

An intriguing finding of this study is the presence of abrupt events that are seemingly not directly correlated with the timing of potential pulses. These non‐continuous behaviors are highlighted for one experiment in Figure 6a, but were observed, to greater or lesser extents, throughout all experiments. To understand the significance of these discontinuities and any trends that may exist, we performed a statistical analysis of all time‐variant modulated potential experiments. Specifically, troughs and peaks exceeding three times the threshold signal variation were identified using self‐written Python code (see Section S1.1.7 for details). To enhance clarity and assist readers in navigating the various data analysis steps, a detailed flowchart was included in the Supporting Information (Figure S8). Figure 6b summarizes the results, showing the quantity of observed discontinuities (centroids) for each experimental condition. The height and width of the violin plots represent the average magnitude of change and standard deviation, respectively. Notably, the number of discontinuities and their magnitude increased with higher electrolyte concentrations. Further comparison between ${\mathrm{CO}}_{2}$‐ and Ar‐bubbled electrolytes (Figure 6c) reveals that ${\mathrm{CO}}_{2}$‐saturation results in both more frequent and more intense discontinuities. Hou et al.^[^ ^13^ ^]^ came to a conclusion along a similar rationale, demonstrating that presence of CO upon the formation of the EDL leads to a weaker, less rigid EDL structure. Interestingly, the number of discontinuities anti‐correlates with more negative potentials, reaching a maximum intensity at a specific potential. This suggests that, as the applied potential becomes more negative, the system stabilizes, leading to fewer disruptions. We may postulate that the number of metastable EDL structures that are near‐equivalent in energy is much higher at lower potentials. We also note that in future work we will further explore these trends, including the strong correlations we find between cation size and the number, and intensity of and potential at which these discontinuities occur most intensely.

Several explanations for the observed abrupt behavior can be argued. One possibility is restructuring of the Cu electrode during reaction, as noted in previous studies,^[^ ^10^, ^11^ ^]^ or bubble formation from a gaseous product. However, a more compelling explanation might involve transitions between metastable states of the EDL. This latter explanation fits better than the former two considering the fact that the discontinuities are mainly observed in PC1, which reflects changes in the diffuse double layer. Bubble formation, furthermore, would likely produce distinct spectral features − such as rotational vibrations^[^ ^94^ ^]^ −‐ which are not observed in the eigenspectra, making this scenario unlikely. Reorganization of the double layer due to the buildup of a critical electric field or potential could be driven by the accumulation of complexated cations, shown to modify local water structure.^[^ ^3^, ^101^, ^102^ ^]^ Specifically, size‐asymmetry of sodium ions (${\mathrm{Na}}^{+}$) and bicarbonate ions was recently shown to lead to local water restructuring.^[^ ^59^ ^]^ This interpretation is also in line with the observation that ${\mathrm{CO}}_{2}$‐saturated solutions exhibit more frequent and intense discontinuities, and with the observation that the structure of water is more optimally packed or ice‐like in Ar‐saturated solutions (Figure 3h).

This restructuring hypothesis also aligns well with the Raman micro‐spectroscopy results shown in Figure 2c, where, following similar approaches as in the literature,^[^ ^103^, ^104^ ^]^ a significant increase in the number of discontinuities is observed when focused slightly further into the electrolyte, rather than directly on the electrode surface. In these experiments, bubble formation and microscopically visible restructuring were also ruled out as the cause of the observed effects.

To further investigate the origins of these discontinuities, i.e., deviations from the description of the time‐decay by a monotonic function, a combined pattern recognition and correlation approach was employed, illustrated in Figure 6d. For each modulated potential experiment, which included over 1000 spectra, the discontinuities were identified across different experimental conditions (applied potential, electrolyte concentration, ${\mathrm{CO}}_{2}$‐ or Ar‐saturation, and in D_2_O or H_2_O). The dynamics of the ten data points (11 s) surrounding each discontinuity in the diffuse double layer were then analyzed using Dynamic Time Warping (DTW)^[^ ^105^ ^]^ and Spearman correlation^[^ ^106^ ^]^ in regards to the measured current and the information extracted from rPC2‐15. The former allows us to account for the degree to which two time profiles (discontinuity compared to the variable of interest) are similar in shape, while the latter yields insights into the directionality (positive or negative) and degree of correlation between the two described by a monotonic function. We examined variables such as the evolution of the integrated areas of the fitted Gaussians, the position of ν(CO)_HFB_ and ν(CO)_LFB_ (which depend on Cu site coordination,^[^ ^90^, ^107^, ^108^ ^]^) local pH, and current to probe activity changes and bubble formation. Figure 6e–h show the Spearman correlation versus DTW alignment cost for these variables across different electrolyte concentrations. Notably, the current shows better correlation with the dynamic behavior of the discontinuities at lower electrolyte concentrations (Figure 6h compared to g, f, and e), while higher concentrations exhibit more discontinuities. The *CO peak center (from which the Stark shift is calculated) also shows high alignment costs and low correlation overall, suggesting that atomic restructuring is not the primary cause of these discontinuities.

Interestingly, changes in the pH (Δ pH), represented by the ratio of carbonate to bicarbonate peaks, show relatively low DTW alignment costs (indicating a good fit) and high positive correlation with the detected discontinuities at 1 M concentration. Additionally, the 2800 cm^−1^ peak, attributed to ν(C‐H) vibrations of hydrocarbons like HCOO, consistently correlates reasonably well. The strongest correlations and lowest alignment costs, however, are systematically found for one key species: *CO at 2085 cm^−1^ (for higher concentrations, 1 M and 0.2 M) and 2078 cm^−1^ (for lower concentrations, 0.1 M and 0.002 M). This suggests that the observed discontinuities, related to water restructuring in the diffuse double layer, directly influence catalytic activity in the interfacial layer. The conversion of ${\mathrm{CO}}_{2}$ to *CO appears to be tightly linked to the proximity of cation species to the electrode and the restructuring of the diffuse double layer.

Further support for this comes from the observation that, with increasing concentration, confined water (2870 cm^−1^) shows the highest correlation, as do adsorbed (complexated) carbonates (1430 cm^−1^). This is notable in light of various theories proposed for the role of cations in the ${\mathrm{CO}}_{2}\mathrm{RR}$. These theories suggest that partially dehydrated ions may assist in the ${\mathrm{CO}}_{2}$ adsorption step through short‐range interactions,^[^ ^86^, ^109^, ^110^ ^]^ that ions may enhance the electric field to stabilize polar intermediates, thereby screening the electric field to suppress byproducts,^[^ ^111^, ^112^, ^113^ ^]^ or that solvated ions assist in buffering the local pH through the hydrolysis of their hydrated shell.^[^ ^87^, ^88^ ^]^ For ${\mathrm{CO}}_{2}\mathrm{RR}$ under neutral aqueous conditions, it seems that cations are consistently involved in ${\mathrm{CO}}_{2}$ adsorption and its conversion to *CO, while Δ pH shows inconsistent correlation (though more significant at higher concentrations). Desolvation of the ions and changes in the apparent electric field (as indicated by the Stark shift; a change in *CO peak center position) are also inconsistent, but more significant at higher concentrations. Thus, we conclude that the influence of cations under these conditions arises from enhanced ${\mathrm{CO}}_{2}$ adsorption through short‐range interactions, noting that this may be accompanied by other mechanisms depending on the cation concentration.

The observed changes in the diffuse double layer must reflect a collective macroscopic rearrangement rather than isolated local events, as our spectroscopic approach is sensitive to cumulative effects.^[^ ^114^, ^115^ ^]^ We postulate that these changes are directly connected to atomic‐level transitions, as suggested by their correlation with *CO. The parameters governing these (predominantly) non‐Faradaic, nonlinear dynamics will be the subject of follow‐up studies. In light of these observations, our most likely explanation for the empirically observed discontinuities is the restructuring of the double layer, which is closely correlated with the turnover of ${\mathrm{CO}}_{2}$. This appears to be a process occurring on the scale of tens of seconds ‐ a timescale that is typically overlooked, and a phenomenon that was likely obscured from detection for the trivial reason that many techniques that resolve double layer behavior assume continuity, or LTI.

## Conclusion

To summarize, in this work, we present an approach of relative conceptual simplicity to examine the nonlinear dynamic nature of the EDL in a well‐established model system: the ${\mathrm{CO}}_{2}\mathrm{RR}$ over Cu. Spectroscopic speciation of distinct features of the EDL is achieved by decomposing sets of two‐dimensional matrices constituted of time‐resolved spectra obtained during the application of a series of potential steps into their time‐variance components and corresponding spectral features. Applying this approach across various concentrations of NaHCO_3_ electrolyte and comparing Ar‐ and ${\mathrm{CO}}_{2}$‐saturated solutions, we find evidence that increased presence of ${\mathrm{CO}}_{2}$ in the solution leads to a less structured EDL. The weaker structural nature of this EDL is not only characterized by static or structural features, i.e., less close packing of the water molecules, but also dynamically, by a higher degree of more intense restructuring of the diffuse double layer. This self‐reorganization of the double layer is highly correlated with ${\mathrm{CO}}_{2}$ adsorption and turnover, which likely stems from the increased proximity of partially solvated ions.

To the best of our knowledge, this is the first report that directly observes double layer restructuring, which significantly affects ${\mathrm{CO}}_{2}\mathrm{RR}$ activity. The fact that noncontinuous processes occur during charging and discharging of the double layer, particularly on timescales that are typically overlooked in theoretical calculations (such as desolvation at ps and capacitance at μs), shines a new light on double layer dynamics. This finding, we believe, has broader implications for the fundamental understanding of double layer effects across electrochemistry, including applications in batteries.

## Conflict of Interests

The authors declare no conflict of interest.

## Supporting information

Supporting Information

## Data Availability

The raw spectroscopic data is available to the public in the following repository: https://zenodo.org/records/14959787.

## References

[1] Z. W. She , J. Kibsgaard , C. F. Dickens , I. Chorkendorff , J. K. Nørskov , T. F. Jaramillo , Science 2017, 355, eaad4998. 28082532 10.1126/science.aad4998

[2] C. Vogt , M. Monai , G. J. Kramer , B. M. Weckhuysen , Nat. Catal. 2019, 2, 188–197.

[3] S.‐J. Shin , D. H. Kim , G. Bae , S. Ringe , H. Choi , H.‐K. Lim , C. H. Choi , H. Kim , Nat. Commun. 2022, 13, 174. 35013347 10.1038/s41467-021-27909-x PMC8748683

[4] A. J. Bard , L. R. Faulkner , Electrochemical Methods: Fundamentals and Applications, Wiley, New York 2001.

[5] W. Schmickler , E. Santos , Interfacial Electrochemistry, Springer, Berlin 2010.

[6] O. Stern , Z. Elektrochem. und Ang. Phys. Chem. 1924, 30, 508–516.

[7] D. L. Chapman , Lond. Edinb. Dubl. Phil. Mag. 1913, 25, 475–481.

[8] M. Gouy , J. Phys. Theor. Appl. 1910, 9, 457–468.

[9] I. Ledezma‐Yanez , W. D. Z. Wallace , P. Sebastián‐Pascual , V. Climent , J. M. Feliu , M. T. M. Koper , Nat. Energy 2017, 2, 17031.

[10] R. Amirbeigiarab , J. Tian , A. Herzog , C. Qiu , A. Bergmann , B. Roldan Cuenya , O. M. Magnussen , Nat. Catal. 2023, 6, 837–846.

[11] P. Grosse , A. Yoon , C. Rettenmaier , A. Herzog , S. W. Chee , B. Roldan Cuenya , Nat. Commun. 2021, 12, 6736. 34795221 10.1038/s41467-021-26743-5 PMC8602378

[12] A. Yoon , J. Poon , P. Grosse , S. W. Chee , B. R. Cuenya , J. Mater. Chem. A 2022, 10, 14041–14050. 10.1039/d1ta11089f PMC9255670 35872703

[13] J. Hou , B. Xu , Q. Lu , Nat. Commun. 2024, 15, 1926. 38431637 10.1038/s41467-024-46318-4 PMC10908862

[14] K. Ojha , N. Arulmozhi , D. Aranzales , M. T. M. Koper , Angew. Chem. Int. Ed. 2020, 59, 711–715. 10.1002/anie.201911929 PMC6973170 31682314

[15] S. Ringe , C. G. Morales‐Guio , L. D. Chen , M. Fields , T. F. Jaramillo , C. Hahn , K. Chan , Nat. Commun. 2020, 11, 33. 31911585 10.1038/s41467-019-13777-z PMC6946669

[16] Y.‐H. Wang , S. Zheng , W.‐M. Yang , R.‐Y. Zhou , Q.‐F. He , P. Radjenovic , J.‐C. Dong , S. Li , J. Zheng , Z.‐L. Yang , G. Attard , F. Pan , Z.‐Q. Tian , J.‐F. Li , Nature 2021, 600, 81–85. 34853456 10.1038/s41586-021-04068-z

[17] M. H. Hicks , W. Nie , A. E. Boehme , H. A. Atwater , T. Agapie , J. C. Peters , J. Am. Chem. Soc. 2024, 146, 25282–25289. 39215715 10.1021/jacs.4c09512 PMC11403608

[18] E. W. Lees , B. A. W. Mowbray , F. G. L. Parlane , C. P. Berlinguette , Nat. Rev. Mater. 2021, 7, 55–64.

[19] Z. Zhang , E. W. Lees , S. Ren , B. A. W. Mowbray , A. Huang , C. P. Berlinguette , ACS Cent. Sci. 2022, 8, 749–755. 35756379 10.1021/acscentsci.2c00329 PMC9228564

[20] A. G. Fink , E. W. Lees , J. Gingras , E. Madore , S. Fradette , S. A. Jaffer , M. Goldman , D. J. Dvorak , C. P. Berlinguette , J. Inorg. Biochem. 2022, 231, 111782. 35349862 10.1016/j.jinorgbio.2022.111782

[21] C. Boone , V. Lochab , M. Fuest , S. Prakash , Solid‐State, Actuators, and Microsystems Workshop Technical Digest, Transducer Research Foundation, Hilton Head, South Carolina, USA 2016, pp. 198–201.

[22] B.‐Y. Wen , J.‐S. Lin , Y.‐J. Zhang , P. M. Radjenovic , X.‐G. Zhang , Z.‐Q. Tian , J.‐F. Li , J. Am. Chem. Soc. 2020, 142, 11698–11702. 32551614 10.1021/jacs.0c05162

[23] A. J. Bard , L. R. Faulkner , Electrochemical Methods: Fundamentals and Applications, (Eds.: D. Harris , E. Swain , E. Aiello ), 2nd ed., John Wiley & Sons, New York 2000, pp. 156–225.

[24] R. Z. Snitkoff‐Sol , A. M. Bond , L. Elbaz , ACS Catal. 2024, 14, 7576–7588.

[25] C. Vogt , F. Meirer , M. Monai , E. Groeneveld , D. Ferri , R. A. Van Santen , M. Nachtegaal , R. R. Unocic , A. I. Frenkel , B. M. Weckhuysen , Nat. Commun. 2021, 12, 7096. 34876582 10.1038/s41467-021-27474-3 PMC8651646

[26] V. Vivier , M. E. Orazem , Chem. Rev. 2022, 122, 11131–11168. 35687869 10.1021/acs.chemrev.1c00876

[27] S. Nitopi , E. Bertheussen , S. B. Scott , X. Liu , A. K. Engstfeld , S. Horch , B. Seger , I. E. Stephens , K. Chan , C. Hahn , J. K. Nørskov , T. F. Jaramillo , I. Chorkendorff , Chem. Rev. 2019, 119, 7610–7672. 31117420 10.1021/acs.chemrev.8b00705

[28] A. Bagger , W. Ju , A. S. Varela , P. Strasser , J. Rossmeisl , ChemPhysChem 2017, 18, 3266–3273. 28872756 10.1002/cphc.201700736

[29] Y. Hori , S. Suzuki , Bull. Chem. Soc. Japan. 1982, 55, 660–665.

[30] Y. Hori , J. Chem. Soc. 1988, 17–19.

[31] S. H. Yalkowsky , Y. He , Handbook of Aqueous Solubility Data, CRC Press, Boca Raton, Florida 2003.

[32] J. Li , Y. Kuang , Y. Meng , X. Tian , W.‐H. Hung , X. Zhang , A. Li , M. Xu , W. Zhou , C.‐S. Ku , C.‐Y. Chiang , G. Zhu , J. Guo , X. Sun , H. Dai , J. Am. Chem. Soc. 2020, 142, 7276–7282. 32250611 10.1021/jacs.0c00122

[33] H. Zhang , J. Gao , D. Raciti , A. S. Hall , Nat. Catal. 2023, 6, 807–817.

[34] J. Hsu , A. M. Eid , C. Randall , M. S. Houache , Y. Abu‐Lebdeh , H. A. Al‐Abadleh , Langmuir 2022, 38, 14789–14798. 36417502 10.1021/acs.langmuir.2c02445

[35] S. Zhu , T. Li , W. B. Cai , M. Shao , ACS Energy Lett. 2019, 4, 682–689.

[36] T. C. Chou , C. C. Chang , H. L. Yu , W. Y. Yu , C. L. Dong , J. J. Velasco‐Vélez , C. H. Chuang , L. C. Chen , J. F. Lee , J. M. Chen , H. L. Wu , J. Am. Chem. Soc. 2020, 142, 2857–2867. 31955572 10.1021/jacs.9b11126

[37] Y. Kim , S. Park , S.‐J. Shin , W. Choi , B. K. Min , H. Kim , W. Kim , Y. J. Hwang , Energy Environ. Sci. 2020, 13, 4301–4311.

[38] M. Dunwell , Q. Lu , J. M. Heyes , J. Rosen , J. G. Chen , Y. Yan , F. Jiao , B. Xu , J. Am. Chem. Soc. 2017, 139, 3774–3783. 28211683 10.1021/jacs.6b13287

[39] Y. Lum , J. W. Ager , Nat. Catal. 2019, 2, 86–93.

[40] R. E. Zeebe , Geochim. Cosmochim. Acta 2011, 75, 2483–2498.

[41] H. Schumacher , U. Künzelmann , K. Künzelmann , B. Vasilev , K.‐J. Eichhorn , J. W. Bartha , Appl. Spectrosc. 2010, 64, 1022–1027. 20828439 10.1366/000370210792434404

[42] T. A. Morhart , S. T. Read , G. Wells , M. Jacobs , S. M. Rosendahl , S. Achenbach , I. J. Burgess , Anal. Methods 2019, 11, 5776–5783.

[43] J. C. Myland , K. B. Oldham , Electrochem. Commun. 2004, 6, 344–350.

[44] M. A. Vorotyntsev , M. D. Levi , D. Aurbach , J. Electroanal. Chem. 2004, 572, 299–307.

[45] T. A. Morhart , B. Unni , M. J. Lardner , I. J. Burgess , Anal. Chem. 2017, 89, 11818–11824. 29019249 10.1021/acs.analchem.7b03509

[46] A. Wuttig , Y. Surendranath , ACS Catal. 2015, 5, 4479–4484.

[47] D. Gao , I. T. McCrum , S. Deo , Y.‐W. Choi , F. Scholten , W. Wan , J. G. Chen , M. J. Janik , B. Roldan Cuenya , ACS Catal. 2018, 8, 10012–10020.

[48] T. Seki , K.‐Y. Chiang , C.‐C. Yu , X. Yu , M. Okuno , J. Hunger , Y. Nagata , M. Bonn , J. Phys. Chem. Lett. 2020, 11, 8459–8469. 32931284 10.1021/acs.jpclett.0c01259 PMC7584361

[49] C.‐C. Yu , K.‐Y. Chiang , M. Okuno , T. Seki , T. Ohto , X. Yu , V. Korepanov , H.‐o. Hamaguchi , M. Bonn , J. Hunger , Y. Nagata , Nat. Commun. 2020, 11, 5977. 33239630 10.1038/s41467-020-19759-w PMC7688972

[50] F. N. Pansini , A. J. Varandas , Chem. Phys. Lett. 2022, 801, 139739.

[51] Y. Katayama , F. Nattino , L. Giordano , J. Hwang , R. R. Rao , O. Andreussi , N. Marzari , Y. Shao‐Horn , J. Phys. Chem. C 2019, 123, 5951–5963.

[52] S. Zhu , B. Jiang , W. B. Cai , M. Shao , J. Am. Chem. Soc. 2017, 139, 15664–15667. 29058890 10.1021/jacs.7b10462

[53] M. E. G. Winkler , R. H. Gonçalves , A. F. Rubira , ACS Omega 2022, 7, 45067–45076. 36530290 10.1021/acsomega.2c05486 PMC9753529

[54] C. Guo , Y. Guo , Y. Shi , X. Lan , Y. Wang , Y. Yu , B. Zhang , Angew. Chem. Int. Ed. 2022, 61, e202205909. 10.1002/anie.202205909 35638153

[55] H. An , J. De Ruiter , L. Wu , S. Yang , F. Meirer , W. Van Der Stam , B. M. Weckhuysen , JACS Au 2023, 3, 1890–1901. 37502158 10.1021/jacsau.3c00129 PMC10369669

[56] R. Ben David , A. R. Head , S. Lin , A. Ben Yaacov , M. A. Andres , B. Eren , Cell Rep. Phys. Sci. 2024, 101890.

[57] M. T. Koper , Advances in Chemical Physics, (Eds.: I. Prigogine , S. A. Rice ), Vol. 92, 1st ed., Wiley, New York 1996, pp. 161–298.

[58] J. T. Edward , J. Chem. Educ. 1970, 47, 261–270.

[59] A. Dodin , G.‐H. Deng , J. A. Rebstock , Q. Zhu , D. T. Limmer , L. R. Baker , Appl. Surf. Sci. 2024, 667, 160345.

[60] D. A. Welch , B. L. Mehdi , H. J. Hatchell , R. Faller , J. E. Evans , N. D. Browning , Adv. Struct. Chem. Imaging 2015, 1, 1.

[61] J.‐L. Fraikin , M. V. Requa , A. N. Cleland , Phys. Rev. Lett. 2009, 102, 156601. 19518661 10.1103/PhysRevLett.102.156601

[62] F. G. Cottrell , Z. Phys. Chem 1903, 42U, 385–431.

[63] B. Huang , R. R. Rao , S. You , K. Hpone Myint , Y. Song , Y. Wang , W. Ding , L. Giordano , Y. Zhang , T. Wang , S. Muy , Y. Katayama , J. C. Grossman , A. P. Willard , K. Xu , Y. Jiang , Y. Shao‐Horn , JACS Au 2021, 1, 1674–1687. 34723270 10.1021/jacsau.1c00281 PMC8549054

[64] K.‐i. Ataka , T. Yotsuyanagi , M. Osawa , J. Phys. Chem. 1996, 100, 10664–10672.

[65] N. L. Odendahl , P. L. Geissler , J. Am. Chem. Soc. 2022, 144, 11178–11188. 35696525 10.1021/jacs.2c01827

[66] Y.‐J. Zhang , Z.‐F. Su , J.‐F. Li , J. Lipkowski , J. Phys. Chem. C 2020, 124, 13240–13248.

[67] M. Osawa , M. Tsushima , H. Mogami , G. Samjeské , A. Yamakata , J. Phys. Chem. C 2008, 112, 4248–4256.

[68] F. Schulz , B. Hartke , Phys. Chem. Chem. Phys. 2003, 5, 5021–5030.

[69] Q. Sun , Vib. Spectrosc. 2009, 51, 213–217.

[70] Y. Liu , B. Zheng , T. Zhang , Y. Chen , J. Liu , Z. Wang , X. Gong , Electrochim. Acta 2022, 432, 141201.

[71] U. Buck , I. Dauster , B. Gao , Z.‐f. Liu , J. Phys. Chem. A 2007, 111, 12355–12362. 18001013 10.1021/jp075717o

[72] M. Osawa , K.‐i. Ataka , Surf. Sci. 1992, 262, L118–L122.

[73] M. Osawa , Bull. Chem. Soc. Jpn. 1997, 70, 2861–2880.

[74] A. Yamakata , M. Osawa , J. Phys. Chem. C 2008, 112, 11427–11432.

[75] A. Yamakata , M. Osawa , J. Phys. Chem. Lett. 2010, 1, 1487–1491.

[76] Q. Sun , Chem. Phys. Lett. 2013, 568–569, 90–94.

[77] Q. Sun , J. Chem. Phys. 2010, 132, 054507. 20136322 10.1063/1.3308496

[78] Q. Sun , Y. Guo , J. Mol. Liq. 2016, 213, 28–32.

[79] U. Buck , C. C. Pradzynski , T. Zeuch , J. M. Dieterich , B. Hartke , Phys. Chem. Chem. Phys. 2014, 16, 6859. 24603719 10.1039/c3cp55185g

[80] D. Shin , J. Hwang , W. Jhe , Nat. Commun. 2019, 10, 286. 30655538 10.1038/s41467-019-08292-0 PMC6336866

[81] D. Bohra , J. H. Chaudhry , T. Burdyny , E. A. Pidko , W. A. Smith , Energy Environ. Sci. 2019, 12, 3380–3389.

[82] J. Xu , J. Tang , Y. Shi , J. Xie , F. Lei , L. Fan , L. Zhang , Appl. Surf. Sci. 2024, 670, 160551.

[83] M. Dunwell , X. Yang , B. P. Setzler , J. Anibal , Y. Yan , B. Xu , ACS Catal. 2018, 8, 3999–4008.

[84] N. Karo , G. Itov , O. Mayraz , C. Vogt , Chem. Eng. J. 2024, 500, 156380.

[85] F. Zhang , A. C. Co , Angew. Chem. Int. Ed. 2020, 59, 1674–1681. 10.1002/anie.201912637 31721382

[86] M. C. Monteiro , F. Dattila , B. Hagedoorn , R. García‐Muelas , N. López , M. T. Koper , Nat. Catal. 2021, 4, 654–662.

[87] M. R. Singh , Y. Kwon , Y. Lum , J. W. Ager , A. T. Bell , J. Am. Chem. Soc. 2016, 138, 13006–13012. 27626299 10.1021/jacs.6b07612

[88] O. Ayemoba , A. Cuesta , ACS Appl. Mater. Interfaces 2017, 9, 27377–27382. 28796478 10.1021/acsami.7b07351

[89] X. Chang , H. Xiong , Y. Xu , Y. Zhao , Q. Lu , B. Xu , Catal. Sci. Technol. 2021, 11, 6825–6831.

[90] C. Zhan , F. Dattila , C. Rettenmaier , A. Herzog , M. Herran , T. Wagner , F. Scholten , A. Bergmann , N. López , B. Roldan Cuenya , Nat. Energy 2024, 9, 1485–1496. 39713047 10.1038/s41560-024-01633-4 PMC11659170

[91] E. L. Clark , J. Resasco , A. Landers , J. Lin , L.‐T. Chung , A. Walton , C. Hahn , T. F. Jaramillo , A. T. Bell , ACS Catal. 2018, 8, 6560–6570.

[92] J. Timoshenko , A. Bergmann , C. Rettenmaier , A. Herzog , R. M. Arán‐Ais , H. S. Jeon , F. T. Haase , U. Hejral , P. Grosse , S. Kühl , E. M. Davis , J. Tian , O. Magnussen , B. Roldan Cuenya , Nat. Catal. 2022, 5, 259–267.

[93] C. Kim , L.‐C. Weng , A. T. Bell , ACS Catal. 2020, 10, 12403–12413.

[94] J. Li , J. Guo , H. Dai , Sci. Adv. 2022, 8, 1–12. 10.1126/sciadv.abo0399 PMC9106293 35559679

[95] T. Sheng , S.‐G. Sun , Chem. Commun. 2017, 53, 2594–2597. 10.1039/c6cc08583k 28191556

[96] S.‐T. Gao , S.‐Q. Xiang , J.‐L. Shi , W. Zhang , L.‐B. Zhao , Phys. Chem. Chem. Phys. 2020, 22, 9607–9615. 32323668 10.1039/c9cp06824d

[97] W. Deng , T. Yuan , S. Chen , H. Li , C. Hu , H. Dong , B. Wu , T. Wang , J. Li , G. A. Ozin , J. Gong , Fundamental Res. 2021, 1, 432–438.

[98] N. T. Nesbitt , W. A. Smith , J. Phys. Chem. C 2021, 125, 13085–13095.

[99] S. Jiang , L. D'Amario , H. Dau , ChemSusChem 2022, 15, 202102506. 10.1002/cssc.202102506 PMC9314821 35289108

[100] H. Ma , E. Ibáñez‐Alé , R. Ganganahalli , J. Pérez‐Ramírez , N. López , B. S. Yeo , J. Am. Chem. Soc. 2023, 145, jacs.3c08079. 10.1021/jacs.3c08079 PMC10655187 37924283

[101] N. García Rey , D. D. Dlott , Phys. Chem. Chem. Phys. 2017, 19, 10491–10501. 28383582 10.1039/c7cp00118e

[102] N. García Rey , D. D. Dlott , J. Phys. Chem. C 2015, 119, 20892–20899.

[103] J. Xu , M. Koh , S. D. Minteer , C. Korzeniewski , ACS Meas. Sci. Au 2023, 3, 127–133. 37090254 10.1021/acsmeasuresciau.2c00064 PMC10120033

[104] C. Korzeniewski , E. M. Peterson , J. P. Kitt , S. D. Minteer , J. M. Harris , J. Electroanal. Chem. 2021, 896, 115207.

[105] J. Zhao , L. Itti , Pattern Recognit. 2018, 74, 171–184.

[106] C. Croux , C. Dehon , Stat. Methods Appl. 2010, 19, 497–515.

[107] C. M. Gunathunge , X. Li , J. Li , R. P. Hicks , V. J. Ovalle , M. M. Waegele , J. Phys. Chem. C 2017, 121, 12337–12344.

[108] P. Eiollins , K. J. Davies , J. Pritchard , Surf. Sci. 1984, 138, 75–83.

[109] S.‐J. Shin , H. Choi , S. Ringe , D. H. Won , H.‐S. Oh , D. H. Kim , T. Lee , D.‐H. Nam , H. Kim , C. H. Choi , Nat. Commun. 2022, 13, 5482. 36123326 10.1038/s41467-022-33199-8 PMC9485141

[110] M. C. O. Monteiro , F. Dattila , N. López , M. T. M. Koper , J. Am. Chem. Soc. 2022, 144, 1589–1602. 34962791 10.1021/jacs.1c10171 PMC8815072

[111] J. Gu , S. Liu , W. Ni , W. Ren , S. Haussener , X. Hu , Nat. Catal. 2022, 5, 268–276.

[112] W. Ren , A. Xu , K. Chan , X. Hu , Angew. Chem. Int. Ed. 2022, 61, e202214173. 10.1002/anie.202214173 36239987

[113] H.‐G. Qin , F.‐Z. Li , Y.‐F. Du , L.‐F. Yang , H. Wang , Y.‐Y. Bai , M. Lin , J. Gu , ACS Catal. 2023, 13, 916–926.

[114] N. Wolff , N. Harting , F. Röder , M. Heinrich , U. Krewer , Eur. Phys. J. Spec. Top. 2019, 227, 2617–2640.

[115] K. Krischer , Advances in Electrochemical Science and Engineering, (Eds.: R. C. Alkire , D. M. Kolb ), Vol. 8, Wiley‐VCH Verlag GmbH & Co. KGaA, Weinheim, FRG 2002, pp. 89–208.

