# Supplementary material for: Decoding Double Layer Dynamics for CO2 Electroreduction over Cu
Source: Angew Chem Int Ed Engl. 2025 Jun 13;64(26):e202423177. doi: 10.1002/anie.202423177 (PMC12184304; doi:10.1002/anie.202423177)
Supplement: Supplementary file 1 — Supporting Information [file ANIE-64-e202423177-s001.pdf]

Supplementary Information to: Decoding Double Layer Dynamics for CO_2 Electroreduction over Cu

Contents

S1 Experimental Methodology 1
S1.1 Experimental Details . . . 1
S1.1.1 Chemicals . . . 1
S1.1.2 Internal Reflective Element Fabrication . . . 2
S1.1.3 Electrode Preparation and Characterization . . . 4
S1.1.4 Operando Electrochemical ATR-SEIRAS . . . 5
S1.1.5 DEMS . . . 7
S1.1.6 Operando Electrochemical Raman micro-spectroscoscopy . . . 8
S1.1.7 Data Analysis . . . 9
S1.2 Operando electrochemical ATR-SEIRAS . . . 10

S2 Additional Discussion on Frequency Analysis and Dimensionality Reduction 12
S2.1 Phase Sensitive Detection . . . 13
S2.2 MCR-ALS . . . 14
S2.3 Matrix Factorization . . . 15

S3 Peak Assignments 21

S4 Supplemental Results 26
S4.1 Operando Electrochemical ATR-SEIRAS . . . 26
S4.1.1 Copper Catalyst . . . 26
S4.1.2 Isotopically Labelled Experiments, Copper Catalyst . . . 43
S4.1.3 Gold Catalyst . . . 46
S4.2 Electrode Characterization . . . 50
S4.3 Determination potential of zero free charge . . . 51

S1 Experimental Methodology

S1.1 Experimental Details

S1.1.1 Chemicals

For the fabrication of internal reflective elements (IREs), the following chemicals were used: H_2SO_4 (98%, J.T.Baker), H_2O_2 (30%, J.T.Baker), HF dip 50:1 solution (J.T.Baker), NH_4OH (29%, J.T.Baker), H_2O_2 (30%, J.T.Baker), AZ®1518 polymeric positive-photoresist (MicroChemicals), tetramethylammonium hydroxide (25%, J.T.Baker), N-methyl-2-pyrrolidinone (J.T.Baker), DMSO-based photoresist remover (TechniStrip® MLO-07,

Technic), and KOH (Bio-Lab). For the preparation and execution of the experiments, the following chemicals were employed: HCl (35.0-37.0%, Daejung), HNO_3 (70%, Bio-Lab), $NaHCO_3$ (Bio-Lab), deuterated water (Aldrich), Chelex® 100 chelatin resin (100-200 mesh, sodium form, BioRad).

S1.1.2 Internal Reflective Element Fabrication

Si IREs were prepared via a photolithographic procedure inside a clean room facility following standard protocols[1,2]. To do so, 4 inches Si(110) wafers (Nova Electronic Materials) were firstly cleaned by immersion in a piranha solution prepared with H_2SO_4 and H_2O_2 with 2:1 volume ratio at 120 °C for 10 min, followed by a bath in a HF dip 50:1 solution, at 20 °C for 10 s. Next, the wafers were immersed in a solution formed with NH_4OH, H_2O_2 and DI water with 1:1:4 aspect ratio at 75 °C, and introduced in an horizontal diffusion furnace (Bruce Technologies Inc, BTI), at 1100 °C with an O_2 fflow rate of 6 slm for 4 h. For the next step, a protective 1100 ± 50 Å layer of silicon nitride (Si_3N_4), as determined through reflectometry (Nanospec 2100), was applied through low-pressure chemical vapor deposition (LPCVD), using a BTI furnace at 780 °C and 280 mT for 30 min with a flow of NH_3 (150 sccm) and SiH_2Cl_2 (50 sccm). Next, a layer of hexadimethylsilazane (Transene) was applied at 110 °C using a Delta 6 VP, followed by the application of an AZ®1518 polymeric positive-photoresist layer. The photoresist was spun at 5000 rpm during 60 s on a Delta 80 RC spin coater, baked at 110 °C for 90 s, irradiated with UV light through an appropriate quartz mask for 4 s on a Karl Suss MA6 mask aligner, and post-baked at 90 °C for 2 min. The sample was then dipped in a tetramethylammonium hydroxide solution, diluted down from the 25% original concentration using DI water at a 1:10 ratio, and further washed with flowing DI water for 1 min before reactive-ion (RIE) etching treatment on a Plasma-Therm 790 MF. Further on, the wafer was washed in a solution of N-methyl-2-pyrrolidinone at 75 °C for 20 min, bathed in MLO-07 photoresist remover at 75 °C for 5 min, and washed with flowing DI water for 1 min, The sample was then dipped in a piranha solution (H_2SO_4:H_2O_2, 2:1) at 120 °C for 10 min , washed with flowing DI water for 1 min, followed by a subsequent bath in a NH_4OH (30%), H_2O_2 (30%) and water solution (1:1:4 ratio) at 75 °C for 10 min. Finally, the wafer was dipped in HF 50:1 solution for 10 s, washed with DI water and dipped in a bath of aqueous KOH (33%) at 75 °C for 1 h 40 min. Photographs of the process can be found in Figure S1.

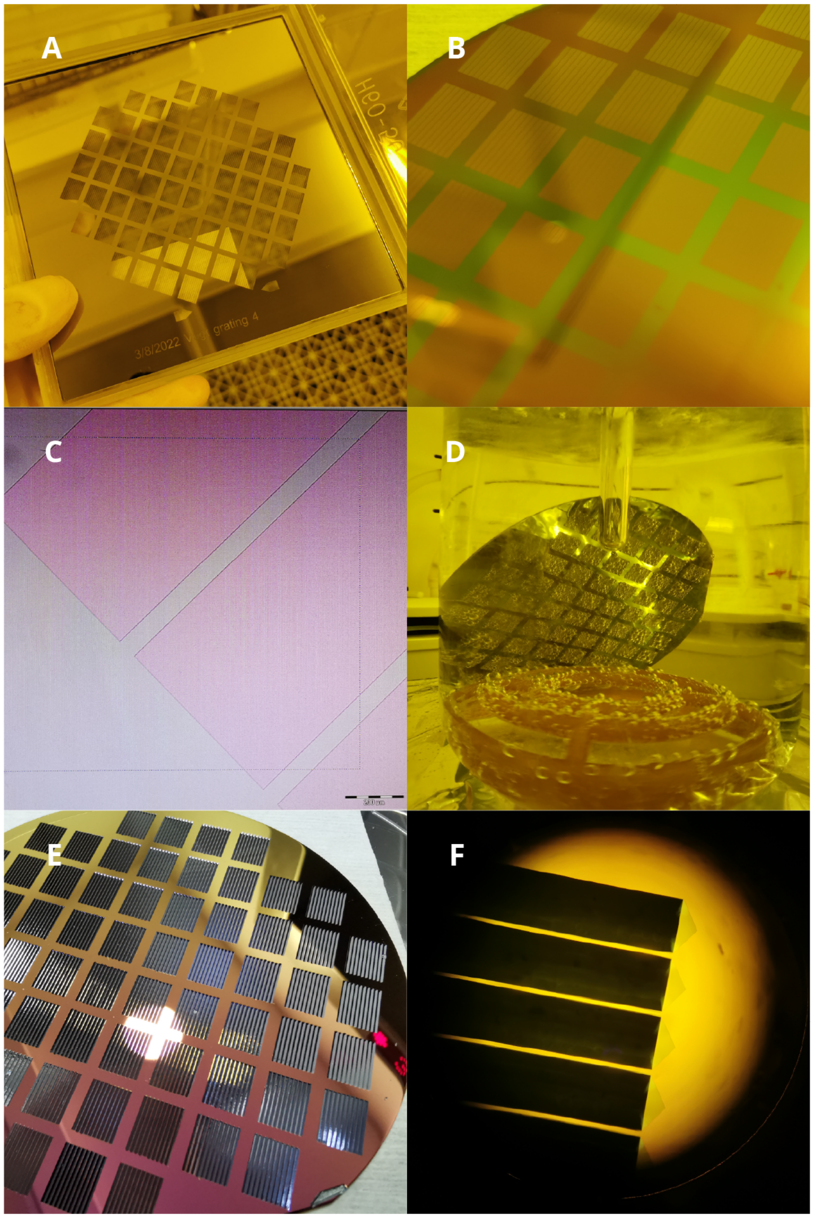

Figure S1: A) Quartz mask employed for the photolithographic process. B) Si wafer covered by photoresist after the irradiation of UV light, and posterior removal of the solubilized photoresist. The marks observed, aligned with the pattern of the quartz mask, show the inner layer of Si_3N_4 after its exposure to open air. C) Optical magnification of the etched patterns after removal of the Si_3N_4 layer *via* RIE removal. D) Si wafer immersed in the KOH 33% solution at 75 °C during the formation of the microgrooves. E) Si wafer after the formation of microgrooves in the KOH bath. F) Optical magnification of the formed microgrooves. The triangular contours towards the tips of the grooves correspond to the etch of Si under the remaining Si_3N_4 layer due to the anisotropy of the etching process.

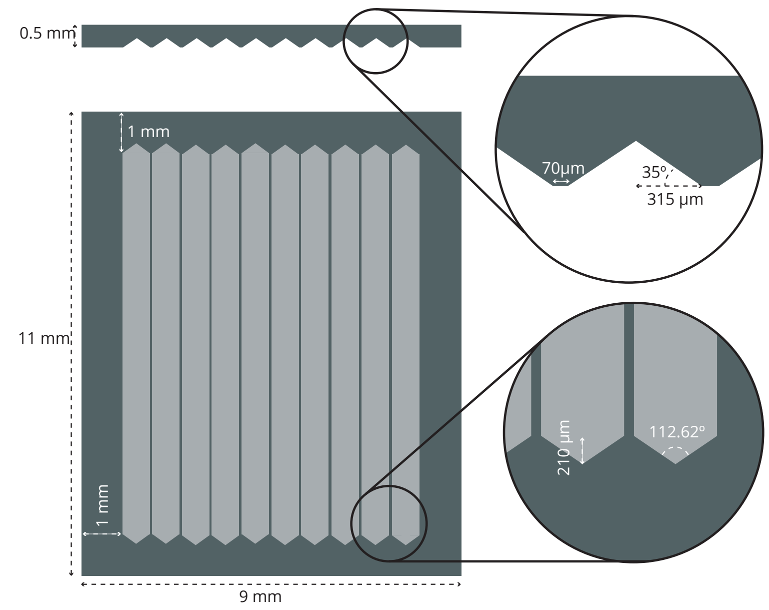

Figure S2: Dimensions of the IREs created for the performance of spectro-electrochemical experiments.

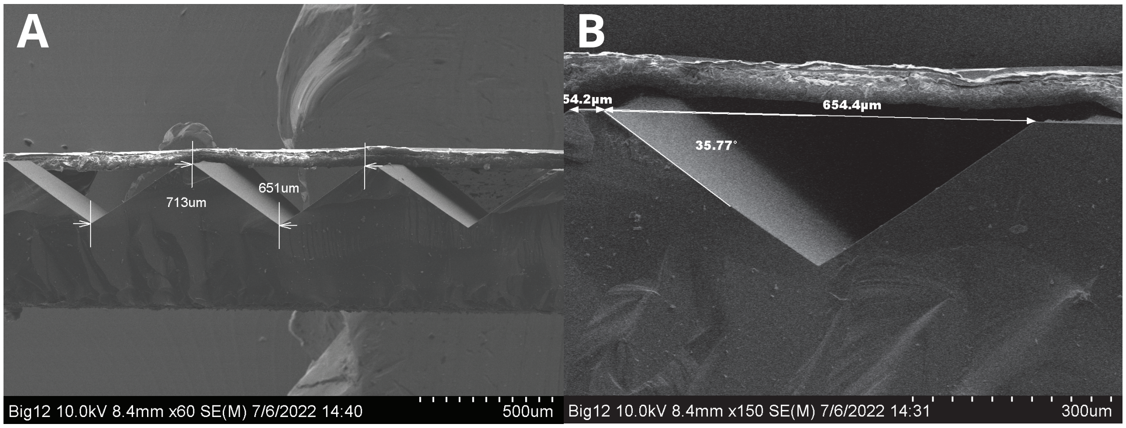

Figure S3: SEM cross-section images of the in-house prepared IREs.

S1.1.3 Electrode Preparation and Characterization

In a typical experiment, Si IREs were treated using aqua regia, prepared by mixing 3 parts of HCl and one part of HNO_3 for 30 min. The flat area of the IREs was then polished on a LaboPol-20 polishing machine using MC-Dac and MD-Nap (Struers) polishing cloths, and diamond slurries of varying diamond diameter size, namely, 6, 3, 1 and 0.25 µm (DiaPro, Struers). Polycrystalline Cu nanolayers (30 nm thick) were physically deposited onto the IREs using an Airco Temescal BJD 1800-Met e-beam evaporator. The surface morphology of the Cu layers was analyzed with atomic force microscopy using an Asylum Research Cypher ES microscope (Oxford Instruments) in air tapping mode, with the use of gold-coated AFM probes (240 AC-NG, Nano Instruments), and blueDrive phototermal excitation (Oxford Instruments, Asylum Research). Images were acquired using AR software (Asylum Research) and further edited through Gwyddion. A Zeiss Ultra Plus high-resolution scanning electron microscope HRSEM (Carl Zeiss) equipped with a Schottky field-emission gun was employed

as well to perform HR-SEM. For these purposes, samples were imaged at working distance of 4-5 mm, an acceleration voltage of 1-2 kV and beam current about 270 pA, using Everhart Thornley ("SE2") secondary electron and pole-piece High-Definition Annular Backscatter (HDAsB) electron detectors, for morphology and atomic mass contrast information respectively. Images were acquired using Smart SEM software (Carl Zeiss). Energy dispersive X-ray spectroscopy (EDS) was used for a direct elemental analysis of the specimens (Bruker, XFlash) inside HR-SEM. For EDS, accelerating voltage of 10 kV and beam current of about 1 nA to perform the were employed on the specimens. Elemental spectra of specific areas of interest and fast intensity maps were acquired with Quantax dedicated software (Bruker, XFlash).

S1.1.4 Operando Electrochemical ATR-SEIRAS

Electrochemical measurements were performed using a polished graphite rod counter electrode (CE) and an Ag/AgCl, KCl saturated (RE-1CP, ALS Japan), reference electrode (RE). The deposited polycrystalline Cu nanolayer acted as the working electrode (WE). The distance between the electrodes was 1 cm^2, approximately. A home-built and designed airtight one-compartment Teflon-and-PEEK electrochemical cell was employed, depicted in Figures S4A and S5. A Palmsens4 potentiostat was used to control the electrical potential. During the potential-pulse experiments, the potential was modulated from -0.4 V_{RHE} to -0.8 V_{RHE} every 100 s, alternating it for five cycles. Cyclic voltammetry experiments were performed sweeping the potential between 0.3 (starting point) and -0.4 V_{RHE} at a scan rate of 0.01 V/s. 0.2 M solutions of $NaHCO_3$ electrolyte, pH = 7 $\pm$ 0.3, were prepared using either ultra pure water (18.2 MΩ at 25°C) or deuterated water. The electrolyte was pre-treated with solid-supported iminodiacetate resin (Chelex) to remove impurities. The solution was then bubbled during 45 min prior to the experiment, and continuously during the with gaseous CO_2 or Ar at a flow rate of 15 mL/min. The potentials in this work are referenced to the reversible hydrogen electrode (RHE), by using the equation: $E_{RHE} = E_{Ag/AgCl} + 0.197 + 0.059pH$.

For the performance of FTIR measurements, the electrochemical cell was placed on a VeeMAX III (Pike Technologies) accessory (Figure S5), and set to an angle of incidence of 35°.

A Nicolet iS50 FTIR spectrometer was employed with a liquid N_2-cooled MCT-A detector. Spectra were recorded at a time interval of 1.10 s and with a resolution of 4 cm^{-1} in the range of 650 to 4000 cm^{-1} at an optical velocity of 2.53 s.

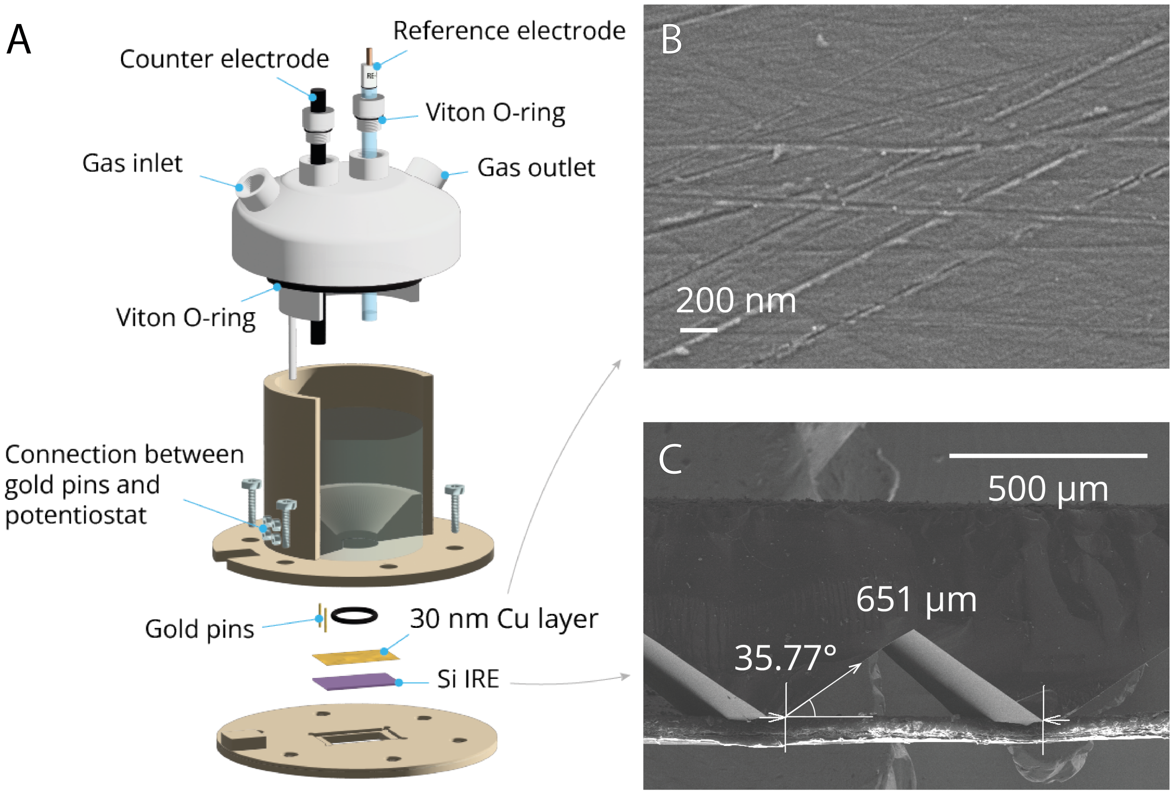

Figure S4: A) Schematic representation of the gas-tight 3-electrode cell, making use of a 30 nm polycrystalline Cu layer deposited onto the internal reflective element (IRE), of which B) shows a high resolution SEM (top view) image, which functions as the working electrode. C) high resolution SEM (side view) image of the IRE, after its fabrication via photolithography.

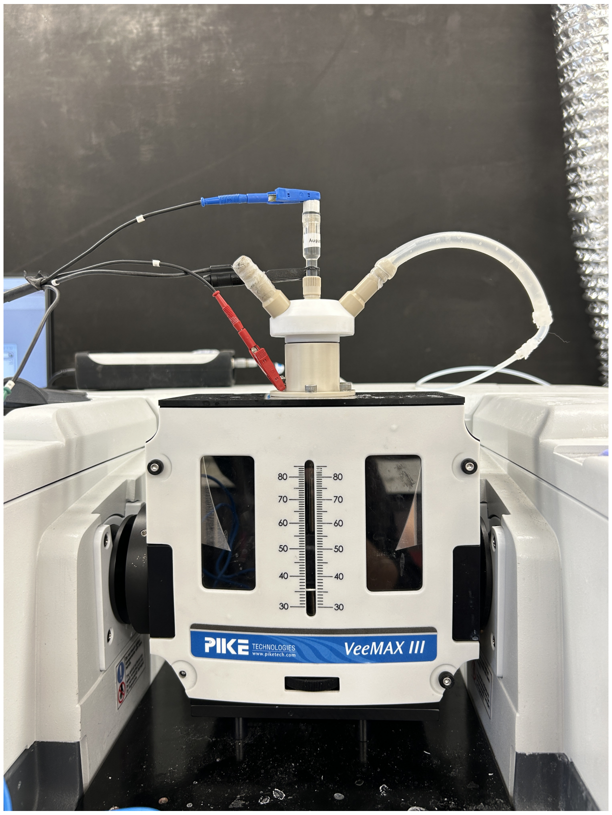

Figure S5: Photograph of the airtight electrochemical cell employed during the *in situ* electrochemical ATR experiments, mounted to the VeeMAX III accessory.

S1.1.5 DEMS

Similar to the electrochemical ATR-SEIRAS experiments, electrochemical measurements were performed using a graphite rod counter electrode and an Ag/AgCl, KCl saturated, reference electrode (RE-1CP, ALS Japan). A commercial polycrystalline Cu electrode (002017, ALS Japan) was employed as working electrode. For the DEMS measurements, a Hiden HPR-40 DEMS was used, equipped with a DEMS probe (with a microporous PTFE membrane) to sample evolved gasses. The dwell and settle time were 7 ms for every species and gas saturation of the electrolyte and chamber were performed equally as explained above. Potential pulses were applied ranging from -0.4 to -1.2 V_{RHE} in a staircase method by decreasing the potential 0.1 V every 300 s.

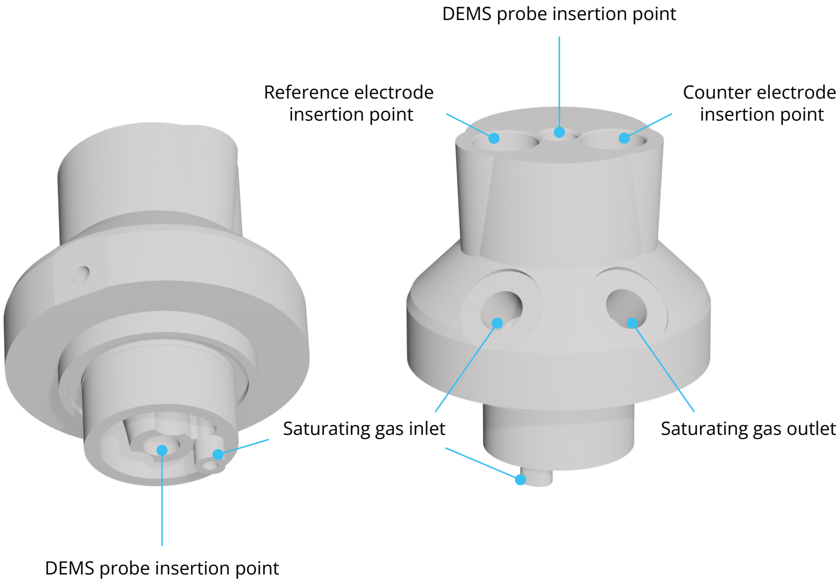

Figure S6: Head of the electrochemical cell employed for the DEMS measurements.

S1.1.6 Operando Electrochemical Raman micro-spectroscoscopy

Raman micro-spectroscopopy experiments were performed following the protocol described in section S1.1.4, with the distinction of the use of a 30 nm-coated glassy carbon as working electrode, due to instrumental limitations. To obtain the spectra, an alpha300 Raman microscope (WITec) was employed using a 633 nm laser source and a power output of 2 mW, with an accumulation time of 2 s. A home-built spectro-electrochemical Raman cell was designed for these experiments, as shown in Figure S7.

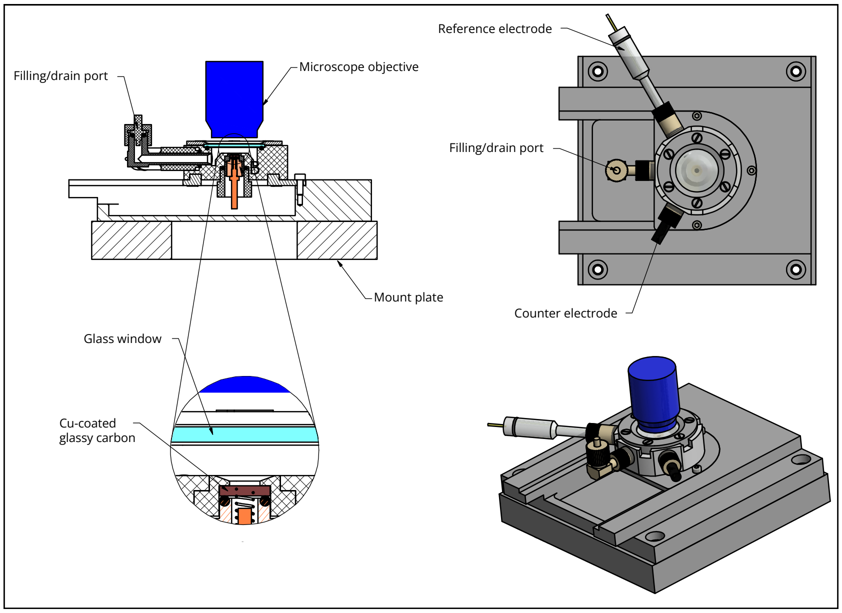

Figure S7: Design of the home-built spectro-electrochemical cell employed during the experiments.

S1.1.7 Data Analysis

Figure S8 presents the overall data analysis procedure in the form of a flowchart. All data analysis was performed in Python and Matlab using self-written code. The covariance matrix was calculated, and further eigendecomposition was carried out using the NumPy package, specifically the `numpy.cov` and `numpy.linalg.eigh` functions. The positions and number of Gaussian curves to fit were selected and fixed based on the peak positions observed in the eigenspectra. When necessary, background correction was performed by interpolating a straight line intersecting the spectra at two variable points and subtracting it from the data. Gaussians for the fits throughout this work were kept at the same mean position for all datasets and not allowed to vary in position (except when discussing the Stark shift). The overall fits yield average R^2 values of 0.991, 0.999, and 0.965 for the raw data, rPC1, and rPC2-15, respectively.

Optimization of the amplitudes of the Gaussian fits, along with the computation of their areas (following background subtraction), was performed using the SciPy package, specifically with the `scipy.optimize.curve_fit` and `scipy.integrate.trapz` functions, respectively. The number and position of the peaks to fit was selected based on the peaks found in the eigenspectra after eigendecomposition of the data (see Section S2.3). For determining Stark tuning rates, the spectral range of the time-series FTIR spectra was reduced to 2200-1900 cm^{-1}. Peaks at 2078 and 2050 cm^{-1} were fitted with Gaussian curves, allowing their mean positions to vary without boundary constraints while recording their shifts along the time axis. Pearson coefficients were computed using the `scipy.stats.pearsonr` function, from which coefficients of determination (R^2) were derived.

For the detection of discontinuities, the scores relative to PC1 derived from the eigendecomposition were smoothed using a Savitzky-Golay filter with an optimized window length and polynomial order to reduce noise while preserving important features (with an average of 10 for the window length, and 2 for the polyorder). Peaks and troughs were detected using the `scipy.signal.find_peaks` function based on prominence criteria, using variable thresholds (with a median value of 5). Additionally, custom discontinuities were identified by comparing the change in signal before and after each datapoint, with significant discontinuities defined as those where the change after a point was at least three times greater than the previous one. Discontinuities with magnitudes below a predefined threshold were filtered out, and those within 15 datapoints (16.5 s) of applied potential changes were excluded to eliminate sharp changes related to the potential switches. Magnitudes of the discontinuity were defined as the absolute difference between the value of the signal at the time of the discontinuity and its neighbouring datapoints.

Following the detection of discontinuities, their surrounding 10 datapoints (11.1 s) were extracted for their correlation with the corresponding datapoints at the same time windows of 1) the integrated areas of the time-series FTIR spectra, calculated as described above, 2) the current measured in the potentiostat, 3) the ratio of the integrated areas relative to the 1430 and 1368 cm^{-1} peaks, and 4) the peak shifts recorded during the Stark tuning rate determination. Subsequently, Spearman correlation coefficients between these four sets of data and the extracted points of the discontinuities were determined using the `scipy.stats.spearmanr` function.

In a similar way, these datapoints were employed to calculate the cost of alignment via Dynamic Time Warping (DTW) using using an algorithm developed by Kamper, based on his code available in GitHub.[3] First, a cost matrix was constructed by calculating the euclidean distance between each datapoint between the two time series to correlate. Using dynamic programming, the optimal alignment path through this cost matrix was determined through the following steps:

1. **Initialization**: The first step was to initialize the cost matrix. The cumulative cost at the starting point $(0, 0)$ was set to the distance between the first points of the two series. The remaining cells in the first row and the first column were initialized to the cumulative cost of aligning points from only one series with the start of the other. This ensures that the alignment begins at the first points of both time series.

2. **Recurrence Relation**: For each subsequent cell (i, j), the cumulative cost was computed by adding the distance at (i, j) to the minimum cumulative cost of its neighboring cells—specifically:

   - The diagonal neighbor $(i - 1, j - 1)$, which represents a match between x_i and y_j,
   - The left neighbor $(i, j - 1)$, representing an insertion in the second time series, and
   - The top neighbor $(i - 1, j)$, representing a deletion in the first time series.

The cumulative cost at each cell (i, j) is given by:

$$\text{cost}(i, j) = \text{distance}(i, j) + \min\left(\text{cost}(i-1, j-1), \text{cost}(i, j-1), \text{cost}(i-1, j)\right)$$

This ensures that the total alignment cost at any point considers the best possible alignment up to that point.

3. **Backtracking**: Once the cost matrix was fully computed, the algorithm backtracked from the last cell (n, m)—the bottom-right corner of the matrix, where n is the length of the first time series and m is the length of the second. By following the path of minimum cumulative cost, the algorithm identified the optimal alignment between the two time series. This path traces the series of matches, insertions, and deletions that minimized the overall alignment cost.

The DTW analysis was conducted on normalized data to ensure consistency across scales, and the non-normalized alignment cost was used as the final metric for comparison between the datasets. The final cost of alignment was defined as the total cumulative cost along this optimal path, starting from $(0, 0)$ and ending at (n, m).

Further details regarding the scripts are available upon reasonable request.

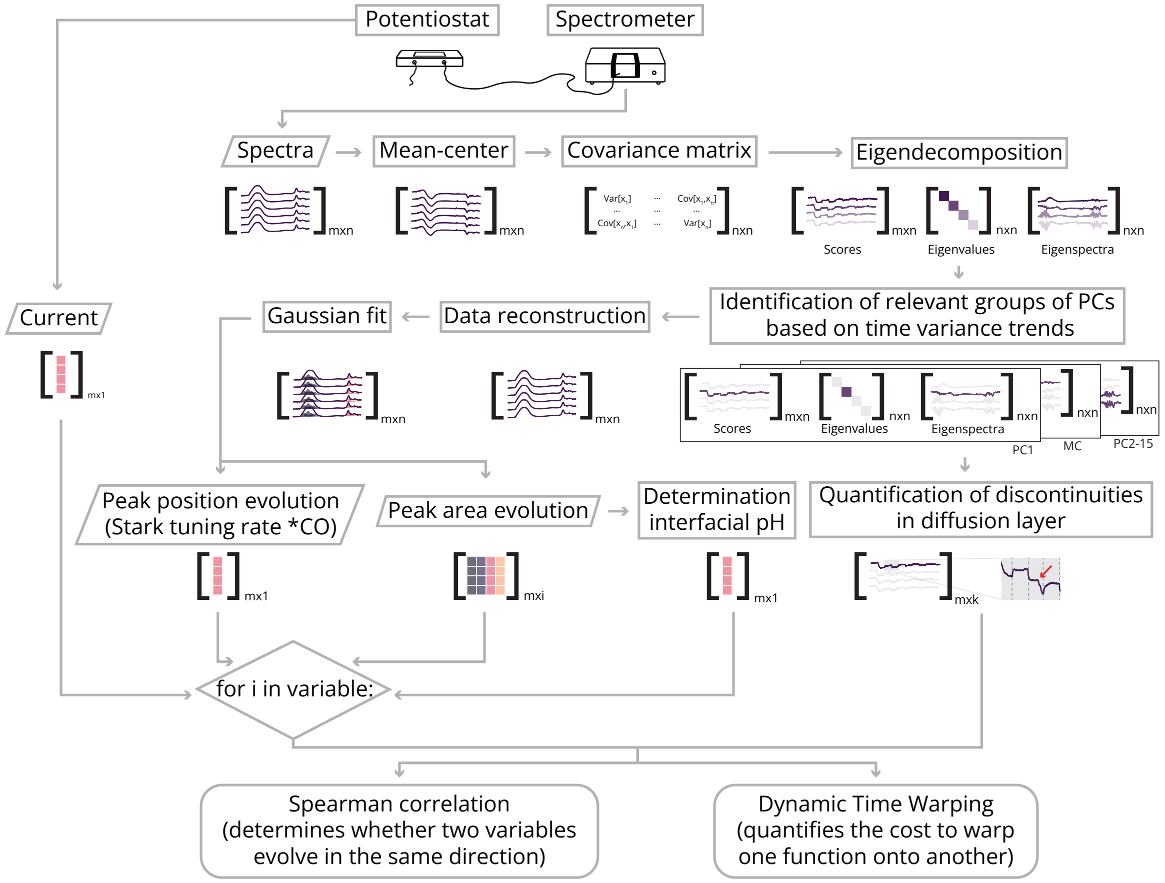

Figure S8: Flowchart of the data analysis techniques employed.

S1.2 Operando electrochemical ATR-SEIRAS

Attenuated Total Reflectance Surface Enhanced Infrared Absorption Spectroscopy (ATR-SEIRAS) is commonly employed for spectro-electrochemical experiments due to its suitability to study the interfacial catalyst/electrolyte

region.[4–7] In brief, this technique exploits the refracting properties of light, governed by Snell´s law,[8] by placing the sample of interest in direct contact with an internal reflection element (IRE), transparent in the region of interest. When the angle of the incident light is greater than that at a critical angle, total reflection of the light occurs at the interface. However, the electric field of the light can still penetrate up to a depth of 1 µm as a transverse non-propagating wave,[6,8] allowing for its characteristic absorption by the sample. This effect can be amplified by incorporating metals or structures onto the surface of the IRE which have a surface plasmon resonance in the IR region. As a result, the oscillating dipole of molecules of interest can interact with surface oscillating dipoles, effectively modifying its dielectric function and, thus, its optical properties.[9,10] Considering that this effect occurs at the vibration frequencies of the oscillating chemical bonds, an amplifier effect can then be obtained, facilitating signal enhancements up to 10^3 within the first 10 nm.[6]

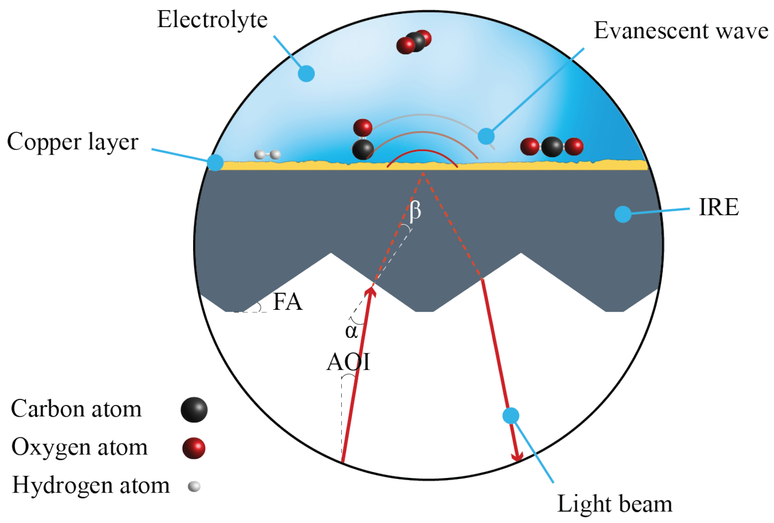

Figure S9: Cross-sectional view of an internal reflective element (IRE) with a (rough) copper layer over which an aqueous electrolyte in which CO_2RR is performed. At the interface between the IRE and the copper layer, or the copper layer and the electrolyte, depending on the incident angle, total internal reflection occurs, and an evanescent wave protrudes into the electrolyte layer able to probe several µm into the electrolyte.

S2 Additional Discussion on Frequency Analysis and Dimensionality Reduction

The high degree of dynamic complexity stemming from processes happening at different (characteristic) time scales, such as slow capacitive charging, or mass transport, and their influence on relatively faster reaction kinetics [11,12] inhibits our understanding, and thus our ability to steer electrocatalytic systems. [13–16] To try to overcome these obstacles, different spectroscopic techniques are employed. One example is electrochemical impedance spectroscopy (EIS), which discriminates the distinct time characteristics of the various electrical, electrochemical and physical processes that take place in an electrochemical system. This is achieved through transformation of the obtained data into the frequency domain, by modeling output current or voltage to its input, which is varied across the addressable AC current or voltage frequency range. [17] EIS, however, cannot directly speciate, i.e., chemically identify, relevant species like reaction intermediates. Operando spectroscopy can aid in this understanding [18–21] but faces several limitations relating to low signal quality, [22] which is especially true in aqueous electrolytes [23] due to strong attenuation of the electromagnetic signal. [24]

Homodyne detection [25–27] i.e., signal demodulation through mixing with a reference signal of a known frequency, is used throughout scientific subdomains. It involves the combination of a known stimulus to the system, followed by deconvolution of the obtained signal via one of the various mathematical descriptions of mixture analysis problems found throughout the breadth of scientific domains. [28,29] In what is generally termed "modulated excitation spectroscopy" (MES), [30–32] an external stimulus of interest is applied, after which demodulation takes place to amplify signals of interest through post-treatment by phase sensitive detection, such as through quadrature demodulation. [31,33] This form of homodyne spectroscopy, MES, however, assumes linear (quasi-)time-invariance. In other words, it is assumed that (quasi-)reversibility exists, that the process is steady state, and that there is no significant deactivation or other non-reversible system perturbation over time. The other of the two broadly classifiable regimes of homodyne spectroscopy is to decompose a system into its fundamental components without assuming any external frequency, or time-variance, which is typically done via matrix factorization (eigendecomposition), sometimes followed by additional steps. [34] Examples include principal component analysis (PCA), which transforms the data into a new coordinate system aimed at describing the most important contributions (via the cumulative variance explained, CVE) to reduce its dimensionality while preserving the maximum amount of information; as well as multivariate curve resolution (MCR); or peak group analysis (PGA), which apply additional constraints such as non-negativity or closure on a given number of components (the number of which are often initially identified through PCA). [28,35–37] Below, the suitability of these examples is tested on a computationally simulated dataset.

The simulated spectral dataset is shown in Figure S10, with a majority species which gradually increases with time-on-stream, a minority species X which has a spiked response to the potential modulation, and a minority species Y which has a differing time-dependent response to the applied square wave potential modulation. The below discusses different commonly applied approaches to resolve trends in such an experiment in detail.

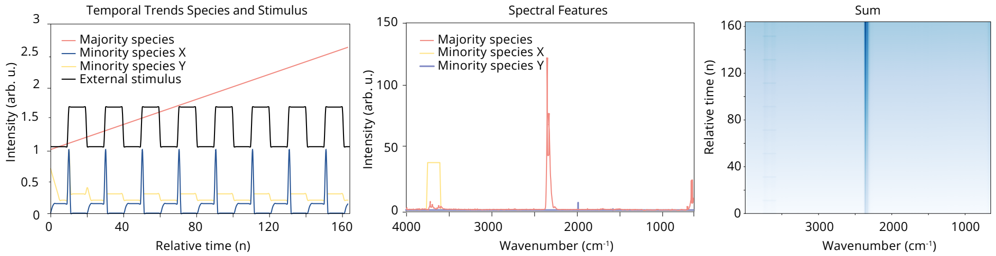

Figure S10: The temporal and spectral features of the simulated data set, including the resulting sum.

S2.1 Phase Sensitive Detection

Such an experiment, where temporal pulses are applied to stimulate (relevant) signal, and the subsequent demodulation of the temporal responses of species with a given mathematical approach is typically referred to as Modulation Excitation (ME).[38–40] As mentioned in the introduction, phase sensitive detection (PSD) is typically applied in combination with modulated excitation, where the demodulation of the spectral data matrix is performed (also called quadrature demodulation) via Equation S1.[31]

$$A_k^{\phi_k^{PSD}}(E) = \frac{2}{T}\int_0^T A(E,t)sin(k\omega t + \phi_k^{PSD})dt \tag{S1}$$

Equation S1 transforms the original signal A, a function of energy E and time t, to a phase domain rather than time. In doing so, the frequency of an external stimulation ω and the demodulation phase angle ϕ_k^{PSD} are used to cancel out all parts of the original signal that do not follow ω. In the equation, k represents the harmonic (k=1, fundamental harmonic), and T = $1/\omega$ as the demodulation period.

Steps have been made to combine the methodology with relevant systems in simple heterogeneous catalytic systems.[30,41] The "phase-sensitive" phrasing stems from the principle that (aside from any component which has a different frequency than the reference signal) any out-of-phase component which has the same frequency as the reference signal is attenuated, which can mathematically be explained by the functional orthogonality properties of sine and cosine functions. That is, if a sine function is multiplied with a cosine one, it is attenuated. As Fourier's theorem states that any function can be described as the sum of sine and cosine functions, one might expect the cosine to also appear in Equation S1.

$$A_k^{\phi_k^{PSD}}(E) = \frac{2}{T}\int_0^T A(E,t)[sin(k\omega t)cos(\phi_k^{PSD}) + cos(k\omega t)sin(\phi_k^{PSD}))]dt \tag{S2}$$

However, due to the contribution of the phase shift and phase angle components in Equation S1, this Fourier property can be simulated a posteriori and therefore manipulated to analyze the response of a dataset to for example (virtual) modulation frequencies. The frequency of ω and ϕ_k^{PSD} (which in practice is an arbitrary input) are applied, leading to the attenuation of all components of the original signal that do not follow ω,[32] such as the contribution of "spectator" signal (the spectral species which are present but do not partake in the reaction). Fourier's theorem states that any arbitrary function can be described by an arbitrary combination of sine and cosine functions, thus allowing for stimulation of an experiment with non-sine wave function stimuli (for example, gases). In essence phase-sensitive-detection is a Fourier transformation, with the addition of phase sensitivity to reduce noise. Some benefits of such analysis have already been proven by the study of basic systems such as the reversible oxidation of noble metals,[32] where the sensitivity of such detection was greatly enhanced. When employed, we are left with phase angle spectra, which contain only spectral information of (surface) changes that modulate at the same angle as the original stimulus (pulses of reactant alternated with H_2). Phase angle spectra (such as those displayed in Figure S11) only contain features of reversible changes. One is thus in principle able to separate bulk from surface (in for example X-ray absorption spectroscopy, were the system is expected to react only at the surface), and spectator species from active ones.

Nevertheless, several issues exist with the use of PSD for real heterogeneous catalytic systems which often undergo irreversible, or non-periodic changes which are of interest. This is arguably the major downside of using PSD, which imposes linear time-invariance constraints and suffers the loss of time resolution beyond a single modulation period due to the required phase-averaging, and the following inability to distinguish time-resolved events which are relevant but non-periodic, for example, a gradual phase change such as represented by the Majority Species in Figure S10. Errors can also be introduced into phase angle spectra as backgrounds can also change with in-phase, which can lead to ambiguities in assignment and its application. While conceptually powerful, one should proceed with appropriate rigor. Figure S11 displays the phase-domain spectra after PSD of the spectra in Figure S10 where the slow phase transition leads to erroneous interpretations of the data.

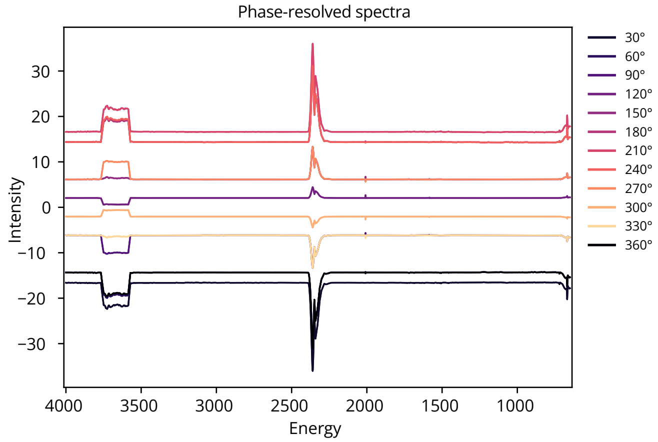

Figure S11: PSD analysis of the simulated data set from Figure S10.

S2.2 MCR-ALS

Multivariate Curve Resolution Alternating Least Squares (MCR-ALS)[28] is an alternative approach to the mixture analysis problem. It is a multivariate analysis approach stemming from seminal work by Lawton and Sylvestre in 1971[29] that covers several algorithms aimed to disentangle non-negative contributions of a given number of components in a mixture. Considering that the responses collected in a time-resolved set of spectra can be represented by data matrix $\mathbf{D}_{p\times n}$ with p(t) rows representing, as per convention, "observation", or in our case, relative time (as a function of spectrum number), and n columns representing "variables", in our case energy points.[42]

$$\mathbf{D} = \sum_{i=1}^{n} \mathbf{c}_i \mathbf{s}_i^T + \mathbf{E} \tag{S3}$$

With variation within $\mathbf{D}$ possibly originating from n sources, $\mathbf{c}_i$ the characteristic that describes the nature of the source of variation, $\mathbf{s}_i^T$ the weight of this characteristic, and $\mathbf{E}$ the unexplained variation by the model. More generally, this can be written as the data matrix $\mathbf{D}$ consisting of individual matrices of the characteristics and their weights, or:

$$\mathbf{D} = \mathbf{C}\mathbf{S}^T + \mathbf{E} \tag{S4}$$

Iterative resolution methods, like MCR-ALS use initial estimates of the $\mathbf{C}$ or $\mathbf{S}^T$ and iterate until the reproduction of the original data set $\mathbf{D}$ by the model $\mathbf{C}\mathbf{S}^T$ is "good enough", determined by a cutoff convergence. The pure components of a matrix are then given, and their concentration profile as a function of relative time.[43] The iteration constraints that are applied in this model in practice lead to the loss of the ability to speciate minority species, particularly ones close to the noise level. Furthermore, non-negativity and closure constraints applied are incompatible with ATR-SEIRAS experiments where the background is typically taken as the system at open circuit potential, and observed signals can thus logically be negative relative to that background. The obtained components are furthermore dominated by the majority signal contributions which is very useful for, e.g., XAS, which adheres quite well to a pure-component description. Figure S12 shows the analysis of our simulated dataset with MCR-ALS, showing indeed that the analyzed mixture shows the progression of the majority species over time.

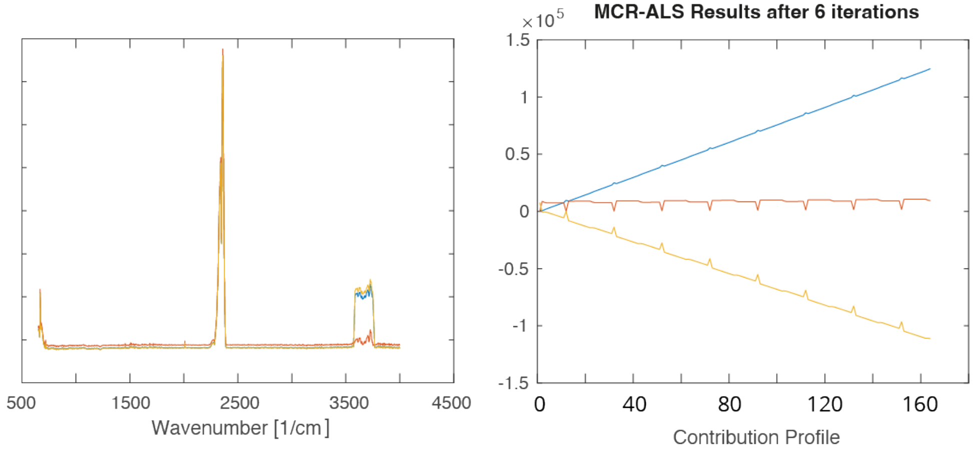

Figure S12: MRC-ALS analysis of the simulated data set from Figure S10.

S2.3 Matrix Factorization

Principal Component Analysis (PCA) is a matrix factorization technique in which our data matrix $\mathbf{D}$ is classified based on variance through decomposition into two new matrices referred to as the loadings ($\mathbf{V}$) and scores ($\mathbf{S}$) matrices, following Equation S5.

$$\mathbf{D} \simeq \mathbf{S}\mathbf{V}^T \tag{S5}$$

This transformation can be achieved by performing an eigendecomposition on the n×n covariance matrix $\mathbf{C}$, described in Equation S6, where $\mathbf{D}_\mu$ corresponds to the mean-centered dataset, i.e., after the subtraction of the mean matrix $\mathbf{M}$, following $\mathbf{D}_\mu$ = $\mathbf{D}$ - $\mathbf{M}$.

$$\mathbf{C} = \frac{1}{n-1}\mathbf{D}_\mu^T\mathbf{D}_\mu \tag{S6}$$

Eigendecomposition of $\mathbf{C}$ is given as

$$\mathbf{C} = \mathbf{V}\mathbf{\Lambda}\mathbf{V}^T \tag{S7}$$

where the λ_i eigenvalues are represented in the diagonal matrix $\mathbf{\Lambda}$, and the patterns in the features (i.e., in the energies employed) are represented by the eigenvector matrix $\mathbf{V}$, which contains the individual eigenvectors $\mathbf{v}_i$. These eigenvectors, whose directions serve as the foundation for the construction of an alternative set of axes called "principal components" (PCs), are linear combinations of the n independent variables, and are aligned in the directions of maximum variance, quantified by their corresponding eigenvalues λ_i. Thus, they provide a new orthogonal basis set that optimally describes the data in terms of its variance. In practice, it is common to refer to $\mathbf{V}$ as the loadings matrix,[44–46] where the individual loading elements ($\mathbf{l}_i$) represent the unstandardized eigenvectors, following the equation $\mathbf{l}_i = \sqrt{\lambda} \cdot \mathbf{v}_i$. These loadings can be graphed against the set of energies registered, creating what is referred to as eigenspectra.[47–49] By performing such transformation on the covariance matrix, we ensure that reduction of the dimensionality of the datamatrix minimizes the loss of physical information, as the dataset is transformed into a new space based on covariance, in which the new eigenvectors are sorted according to the quantity of information that they contain. That is, the degree of the variance captured in each PC is described by the eigenvalue corresponding to that PC, which can thus be used to sort the eigenvectors according to the degree of variance that it explains within the dataset. It is important to note that, while the eigenvectors in such a transformation of a spectral data matrix resemble spectra, they do not directly contain physically meaningful chemical information. In other words, the observed "peaks" do not correspond to spectral features, but rather to directions of maximum variance in the original data.

The evolution of the spectra along the time axis is captured by the scores, s_i. These scores are encompassed in the score-matrix $\mathbf{S}$, and can be calculated by projecting the original data matrix $\mathbf{D}_\mu$ onto the newly constructed

low-dimensional space, via $\mathbf{S} = \mathbf{D}_\mu \mathbf{V} \mathbf{\Lambda}^{(-1/2)}$.[44,50] Each individual score element $\mathbf{s}_i$ thus contains information about existing correlations or patterns of the species during the experiment.[50] Through analysis of the time-resolved trends in $\mathbf{S}$, which can be done either quantitatively, e.g., through further decomposition, or in a qualitative manner, we may select and even characterize relevant contributions to the system variance. This can be used to group chemical species with the same time-variance through linear reconstruction of (groups of) relevant loadings and scores (Eq S8), excluding contributions both from the noise and differently time variant responses.

$$\mathbf{D}_i = \mu + \sum_i \mathbf{s}_i \mathbf{v}_i \tag{S8}$$

Care must always be taken not to over-analyze,[50] yet analysis of the scores matrix - in principle - allows for separation of differently time-variant events granted they sufficiently differ in their variance contribution. Hence, this allows us to separate slow diffusion processes towards the electrode of species with large variance contributions from fast, pulse-responsive small variance correlations between, for example, different species more closely related to the reaction.[51,52]

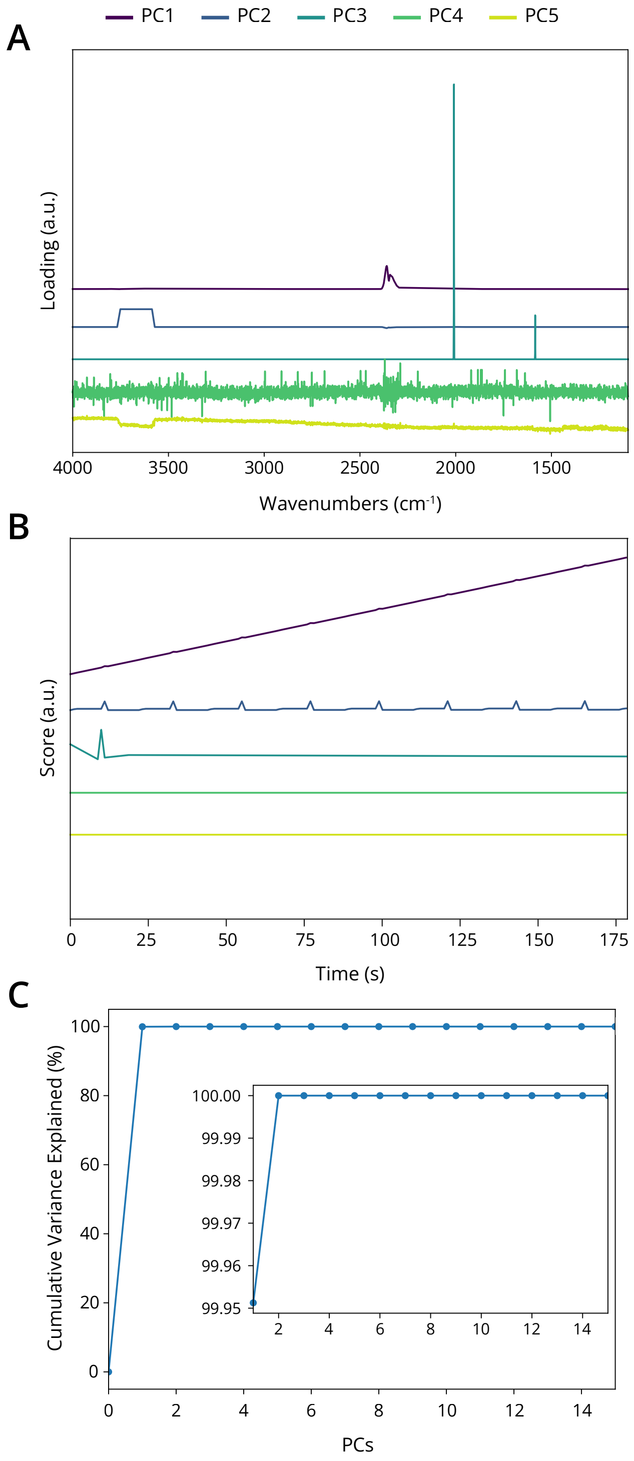

Figure S13: A) The eigenspectra (right singular matrices) of the eigendecomposition of the covariance matrix (principal component analysis) obtained from the simulated spectral data matrix shown in Figure S10. B) The scores plotted against relative time resulting from the eigendecomposition of the covariance matrix (principal component analysis) calculated from the simulated spectral data matrix shown in Figure S10. C) The cumulative variance explained by the first 15 eigenvectors derived from the same eigendecomposition

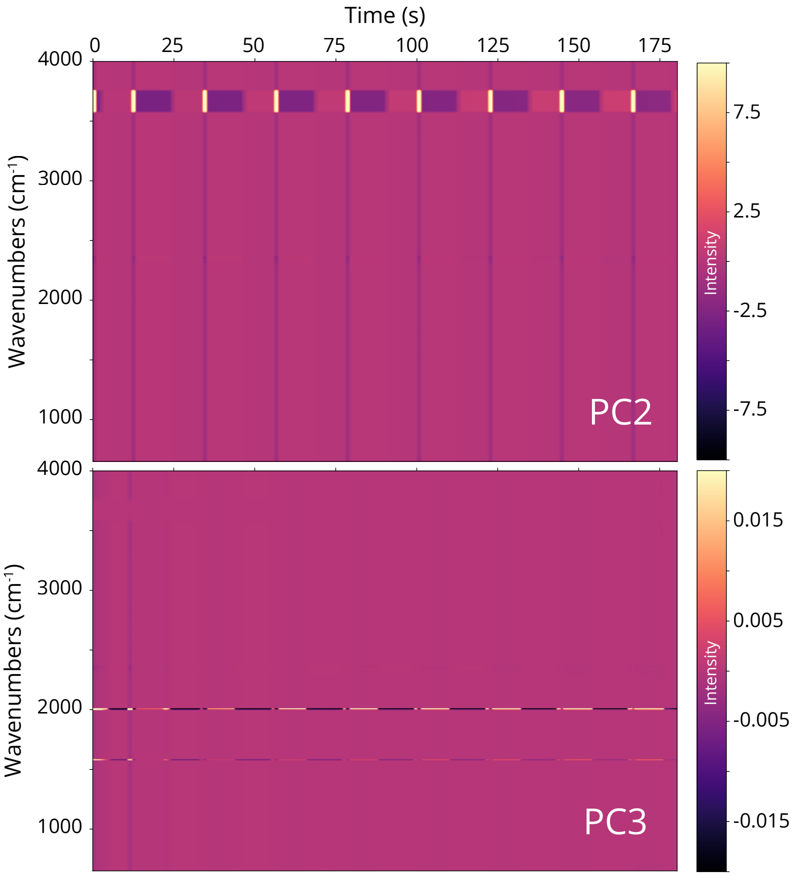

Figure S14: Reconstructed datamatrices using the eigenspectra and scores (Figure S13) of the simulated spectral data shown in Figure S10.

Figure S13A shows the eigenspectra obtained from the application of this process onto the simulated spectral dataset. As it can be observed, the resulting eigenspectra clearly hint at the chemical region contributing the most to each PC, since the major sources of variance captured by the eigenvectors correspond to the energies where chemically meaningful peaks are found. While examination of the eigenspectra can be helpful to understand which species from the original spectra are correlated, evaluation of the time-dependent behavior of these signals allows one to distinguish how meaningful a PC truly is in relationship to the applied experiment. That is, trends in the scores matrix correlating to, for example, the modulation pulse frequency are easily identifiable as likely to contain information relevant to your applied stimulus. The values of the elements in $\mathbf{S}$ can be plotted as a function of relative time (n) or spectrum number, which allows for the assessment of time-resolved patterns, as can be observed on Figure S13B. Figure S13B shows the scores plotted as a function of relative time related to the first five PCs of the simulated dataset. As can be observed, PC1 captures the time-resolved trend of the majority species of the simulated dataset, while the subsequent PCs 2 and 3 reveal the time-resolved trends of the minority species. Indeed, we were able to separate the three previously described behaviors, i.e., the slow evolution of an intense species throughout the course of the experiment (PC1), the spiked dynamic response of minority species X to the applied potential, as well as the differing temporal response of the signal that only contributed $\pm$ 1% to the overall signal. As our original dataset can at any time be reconstructed through the summation of the product of the loadings and scores of each PC such as described in Eq S8, we may compose a new datamatrix of a chemically and temporally meaningful subset of those products.

While PCA has traditionally mainly been applied as a tool for noise reduction across scientific disciplines, we thus can exploit its properties instead as a valuable tool to evaluate the kinetic contribution of a (group of) PCs with similar relevant time-variant properties. This works particularly well for the described mixture analysis problem

for two main reasons. Firstly, if the variables in the original data have different scales, such as for our simulated spectral data set, the covariance matrix will still capture the linear relationships between them appropriately. Secondly, the normalized eigenvectors of the covariance matrix, are orthonormal to each other in the PC space. However, in the original feature space, where the original feature vectors are based on physical variables, they may not be orthogonal due to potential correlations between physical properties. This non-orthogonality in the original feature space reflects the potential redundancy or correlation between the physical parameters. Figure S13A includes the eigenspectra of PCs 1, 2 and 3, which resemble the individual components of the original set of simulated spectra (Figure S10). For example, if we were interested in reconstructing the spectra to display those chemical species which react to the potential pulses applied (i.e., minority species X and Y from Figure S10), based on the scoreplots displayed in Figure S13B we would focus on PCs 2 and 3, but not on PC1. PC1, however, contributes about 99.95% of the total variance explained (Figure S13C), and thus by removing its contribution, and examining it separately from the temporally responsive minority species allows us to significantly enhance their signal despite their minute contributions to the overall intensity. The reconstructed spectral data matrix, following EqS8, with the minority species is shown in Figure S14. Considering that the low variance contributions related to certain components are still discernible by the technique, we decided to further test its limits by introducing a fourth contribution to the simulated dataset, constituted by random noise at three different intensities, i.e., 0.5, 1 and 2 times the intensity of Y(Figure S15). After the performance of PCA on these new datasets, the detection of Y was confirmed in its corresponding eigenspectra for signal-to-noise ratios equating 1, but its contribution was indiscernible when the noise is twice as intense as the signal of interest. Therefore, we conclude that this method is restrained to the detection of signals with intensities equal to or higher than the noise level.

Nevertheless, PCA seems especially suited amongst the discussed analysis methods to elucidate the time-dependent behavior that we input, and account for both the entire time-resolved species evolution throughout the experiment (Majority X), as well as the minority species contribution which were clouded in both MCR-ALS, and PSD. Despite the transformation, the information contained in the original dataset is still retained in the new described space, since the variance is indirectly a descriptor of information content. As such, in summary, PCA is used to reduce the dimensionality of the n-dimensional datamatrix while retaining most of the information.[44–46,53]

To conclude, and while these methodologies are easily computed with pre-existing programming libraries, we include here a formal description of how the eigenvalues and eigenvectors can be calculated. Mathematically, $\mathbf{v}_i$ and λ_i are defined by Equation S9:

$$\mathbf{C}\mathbf{v}_i = \lambda_i \mathbf{v}_i \tag{S9}$$

To calculate the eigenvalues and eigenvectors, Equation S9 can be rearranged, as shown below, where I is the identity matrix:

$$(\mathbf{C} - \lambda_i \mathbf{I})\mathbf{v}_i = 0 \tag{S10}$$

For Equation S10 to have non-trivial solutions, that is, $\mathbf{v}_i \neq 0$, it is required for $\mathbf{C} - \lambda_i \mathbf{I}$ to be treated as a singular matrix, *i.e.*, as non-invertible. To achieve such goal, and based on the properties of singular matrices, its determinant must equal zero, as shown in Equation S11.[54,55]

$$\mid \mathbf{C} - \lambda_i \mathbf{I} \mid = 0 \tag{S11}$$

The resolution of Equation S11 retrieves the different values of λ_i, which can then be incorporated into Equation S10, to retrieve their corresponding eigenvectors. After sorting these calculated n eigenvectors and eigenvalues, $\mathbf{C}$ can be transformed into the new coordinate space defined by them through Equation S7.

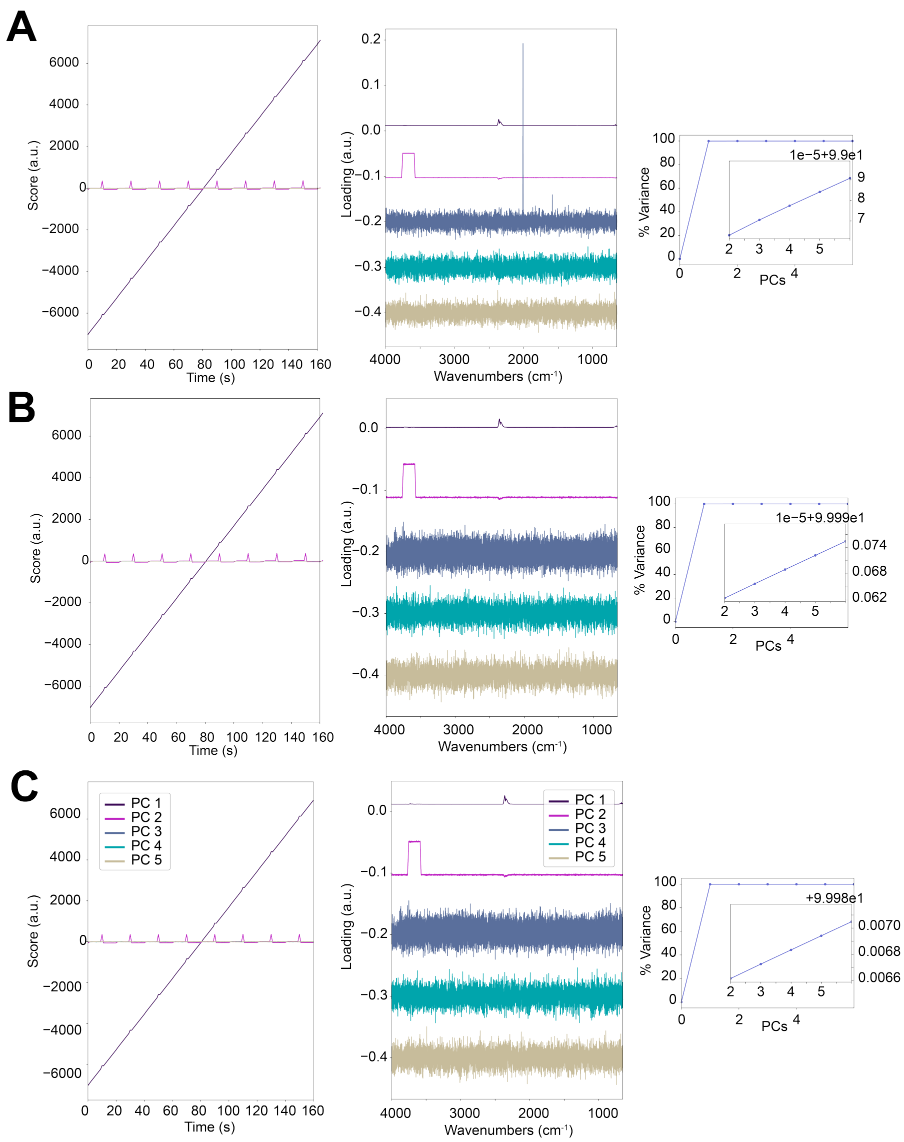

Figure S15: Noise sensitivity analysis of the applied methodology. Left, the scores, middle, loadings and right, scree plots of PCA performed on the dataset from Figure S10 with the addition of a random noise matrix of A) 0.5x, B) 1x, and C) 2x the maximum intensity of the minority species Y.

S3 Peak Assignments

Observed peak center in the potential modulated CO_2RR reactions with CO_2-, and Ar-bubbled 0.2 M $NaHCO_3$ electrolyte in H_2O in this work.

**Obs. Peak Center (cm^{-1})**	**Obs. in PC (CO_2 experiments)**	**Reaction Environment**	**Assignment**	**Ref.**
3520	Mainly PC2 to 15	CO_2 and Ar	ν(OH), DDA-OH	[56–59]
3360	Mainly PC1, and PC 2 to 15	CO_2 and Ar	ν(OH), DA-OH	[56–59]
3210	Mainly PC1, and PC 2 to 15	CO_2 and Ar	ν(OH), DDAA-OH	[56–59]
2367	PC2 to 15	CO_2 and Ar	$\nu_a(CO_2)$, R branch	[60–62]
2320	PC2 to 15	CO_2 and Ar	$\nu_a(CO_2)$, P branch	[60]
2127	PC1	CO_2 and Ar	Libration+bend from H_2O	[60,63]
2078	PC2 to 15	CO_2 and Ar	ν(CO) from *CO	[61,62,64–66]
1639	PC1, and PC 2 to 15	CO_2 and Ar	$\delta(H_2O)$	[67–70]
1541	PC 1, and PC2 to 15	CO_2 and Ar	ν_a(OCO) from $HCOO^-$	[71,72]
1430	PC2 to 15	CO_2 and Ar	ν(C=O) from CO_3^{2-}	[71,73]
1400	PC2 to 15	CO_2	CH bending from $*OCH_3$	[64]
1368	PC1, and PC2 to 15	CO_2 and Ar	ν(C=O) from HCO_3^-	[74,75]
1270	PC1, and PC2 to 15	CO_2	ν(C-OH) hydrogenated CO (dimer)	[76,77]
1170	PC1, and PC2 to 15	CO_2	ν(C-OH) hydrogenated CO (dimer)	[76,77]
1227	PC1, and PC2 to 15	CO_2 and Ar	Longitudinal optical mode of asymmetric stretching vibration of ultra-thin SiO_X	[78]

Fitted peak centers based on the peak positions from eigenspectra in the potential modulated CO_2RR reactions with CO_2- and Ar-bubbled 0.2 M $NaHCO_3$ electrolyte in H_2O in this work.

**Obs. Peak Center (cm^{-1})**	**Assignment**	**Ref.**	**Standard deviation (cm^{-1})**
3680	Free-OH / $\nu_a(CO_2)$ + $\nu_s(CO_2)$ combination band	[69,79–87]	70
3520	ν(OH), DDA-OH	[56–59]	100
3360	ν(OH), DA-OH	[56–59]	100
3210	ν(OH), DDAA-OH	[56–59]	80
3100	ν(OH), DAA-OH	[56–59]	100
2870	ν(OH), A^+-OH	[56–59]	80
2800	ν(CH) from hydrocarbonated species	[71]	80
2367	$\nu_a(CO_2)$, R branch	[60–62]	10
2350	$\nu_a(CO_2)$, $CO_{2(aq)}$	[60]	10
2320	$\nu_a(CO_2)$, P branch	[60–62]	10
2127	Libration+bend from H_2O	[60,63]	100
2085	HFB ν(CO) from *CO	[65]	30
2078	ν(CO) from *CO	[61,62,64–66]	10
2050	LFB ν(CO) from *CO	[65]	30
1800	$\nu(CO)_{bridge}$ from *CO	[76,88]	100
1700	ν(C=O) from *HCO	[66,76,89]	30
1639	$\delta(H_2O)$	[67–70]	40
1610	ν(C=O) from HCOO	[64]	40
1541	ν_a(OCO) from $HCOO^-$	[71,72]	40
1508	ν(C-O) from $*CO_3^-$	[64]	40
1430	ν(C=O) from CO_3^{2-}	[71,73]	30
1368	ν(C=O) from HCO_3^-	[74,75]	40
1270	ν(C-OH) hydrogenated CO (dimer)	[76,77]	40
1227	Longitudinal optical mode of asymmetric ν(Si-O) of ultra-thin SiO_X	[78]	40
1170	ν(C-OH) hydrogenated CO (dimer)	[76,77]	40

Observed peak center in the potential modulated CO_2RR reactions with CO_2-, and Ar-bubbled 0.2 M $NaHCO_3$ electrolyte in D_2O in this work.

**Obs. Peak Center (cm^{-1})**	**Obs. in PC (of CO_2 experiments)**	**Reaction Environment**	**Assignment**	**Ref.**
3400	Mainly PC1	CO_2 and Ar	ν(OH)	[80,83,86,87]
3360	Mainly PC1, and PC 2 to 15	CO_2 and Ar	ν(OH), DA-OH	[56–59]
2600	PC1, and PC 2 to 15	CO_2 and Ar	ν(OD), DA-OD	[56–59]
2470	PC1, and PC 2 to 15	CO_2 and Ar	ν(OD), DDAA-OD	[56–59]
2390	PC2 to 15	CO_2 and Ar	ν(OD), A^+	[56–59]
2330	PC1, and PC2 to 15	CO_2 and Ar	$\nu_a(CO_2)$, P branch	[60–62]
2290	PC2 to 15	CO_2 and Ar	ν(CD)	[90,91]
2071	PC2 to 15	CO_2 and Ar	ν(CO) from *CO	[61,62,64–66]
1625	PC1, and PC 2 to 15	CO_2 and Ar	$\delta(H_2O)$	[67–70]
1560	PC2 to 15	CO_2 and Ar	ν_a(OCO) from $HCOO^-$	[71,72]
1460	PC1	CO_2 and Ar	δ(HOD)	[67,92]
1430	PC1	CO_2 and Ar	ν(C=O) from CO_3^{2-}	[71,73]
1365	PC1, and PC2 to 15	CO_2	ν(C=O) from HCO_3^-	[74,75]
1204	PC1, and PC2 to 15	CO_2 and Ar	$\delta(D_2O)$	[67,92]

Table S4: An overview of relevant infrared peaks assigned in literature, along with characteristics of the different studies such as the electrolyte the reaction was performed in, the potential the peak was observed (if relevant), as well as whether the study used isotopic labeling to confirm the assignment.

**Sample**	**Electrolyte**	**Peak center (cm^{-1})**	V_{RHE}	**Assignment**	**Ref.**	**Isotopic labeling**
Cu thin film ("wet chemical strategy")	$KHCO_3$	2343	-0.5	CO_{2aq}	66	
		2083-2052	-0.5	ν(Cu-CO)		
		1725-1717	-0.6	ν(C=O)		
		1618-1600	-0.2	δ(H-O-H) of H_2O		
		1544-1517	0.2 to -1.3	$*CO_3^{2-}$		
		1402	-0.1	ν_s(O-C-O) of COO-ad		
		1390	-0.6	Solution CO_3^{2-}		
		1379	-0.3	ν(C-O) of COOH		
Cu film ("chemically deposited")	0.1 M $NaHCO_3$	2075	-0.66	ν(Cu-CO) HFB	93	
		2032	-0.66	ν(Cu-CO) LFB	93	
Pt (111), Pt(110)	HF	1530	+0.6 to +0.9	$*CO_3^{2-}$	94	
		1410	+0.55 to +0.9	Solution CO_3^{2-}		
Au(111)	0.1 M Na_2CO_3	1629	N.A.	HCO_3^-	95	D_2O
		1450-1511	N.A.	$*CO_3^{2-}$		
		1397	N.A.	Solution CO_3^{2-}		
		1362	N.A.	HCO_3^-		
Cu(100) and poly-crystalline Cu	0.2 M K_2CO_3	1545	-0.4	$*CO_3^{2-}$	96	D_2O
Cu thin film	0.1 M $KHCO_3$	1544-1517	+0.2 to -1.3	$*CO_3^{2-}$	66	
		1394	-0.9	Solution CO_3^{2-}		
Polycrystalline Cu	0.1 M NaOH	1710	N.A.	ν(CH) - formic acid		
		1710	N.A.	ν(C=O) - formic acid	71	
		1627	-0.8 to -1.3	HCO_3^-		
	$NaHCO_3$	1610-1584	-0.06 to -0.63	ν_a(OCO) - $HCOO^-$ (coordinated) in solution		
		1410	-0.9	Solution CO_3^{2-}		
		1365	-0.63 to -1.36	HCO_3^-		
Cu(100)	IRAS – Surface science study	2891-2900	N.A.	ν_s(CH) from $HCOO^-$	97	

Sample	Electrolyte	Peak center (cm^{-1})	V_{RHE}	Assignment	Ref.	Isotopic labeling
		1348-1358	N.A.	ν_s(COO) from $HCOO^-$		
Electrochem. deposited Cu film	0.5 M $NaHCO_3$	2090, 1396	+0.1 to -0.7	H_{ad}, $\nu_s(COO^-)$	[98]	D_2O, $^{13}CO_2$
		1396	N.A.	$\nu_s(COO^-)$		
Cu electrode (bulk, polished)	0.1 M $TEABF_4$ in ACN	1681	N.A.	CO_3^{2-}	[75]	D_2O
		1646	N.A.	CO_3^{2-}		
		1607	N.A.	HCO_3^-		
		1487	N.A.	HCO_2^-		
		1452	N.A.	HCO_2^-		
		1388	N.A.	HCO_2^-		
		1364	N.A.	CO_3^{2-}		
		1328	N.A.	CO_3^{2-}		
		1305	N.A.	CO_3^{2-}		
		1213	N.A.	HCO_2^-		
Cu(100)	0.1 M LiOH	1677	+0.05 to -0.2	ν(C=O) – CO hollow Cu(100)	[77]	D_2O, $^{13}CO_2$
		1584	+0.05 to -0.2	ν(C=O) – hydrogenated dimer OCCOH		
		1191	+0.05 to -0.2	ν(C-OH) – hydrogenated dimer OCCOH		
		1195	N.A.	ν(C-OH) – Methanol		
		1195, 1712, 1666	N.A.	Acetaldehyde		
		1212	N.A.	ν(C-OH) - HOC≡COH		

S4 Supplemental Results

S4.1 Operando Electrochemical ATR-SEIRAS

S4.1.1 Copper Catalyst

It is important to highlight that many of the signals described in the main text can also be found in the control experiments conducted, where the $NaHCO_3$ solution was saturated with Ar instead of CO_2 (Figure S17), albeit with (sometimes) lower intensities, due to the use of a bicarbonate salt as electrolyte, as is often customary on CO_2RR studies.[60,73,76,99–107] The only other dominant signal observed is the sharp peak at 2334 cm^{-1}, associated to dissolved $\nu(CO_2)$[61,71,108] which is virtually absent in the results from the control experiment. It is thus also not surprising that (bi)carbonate electrolytes are most often employed in spectroscopic CO_2RR studies, to increase the concentration of detectable carbon-derived species in the solution.

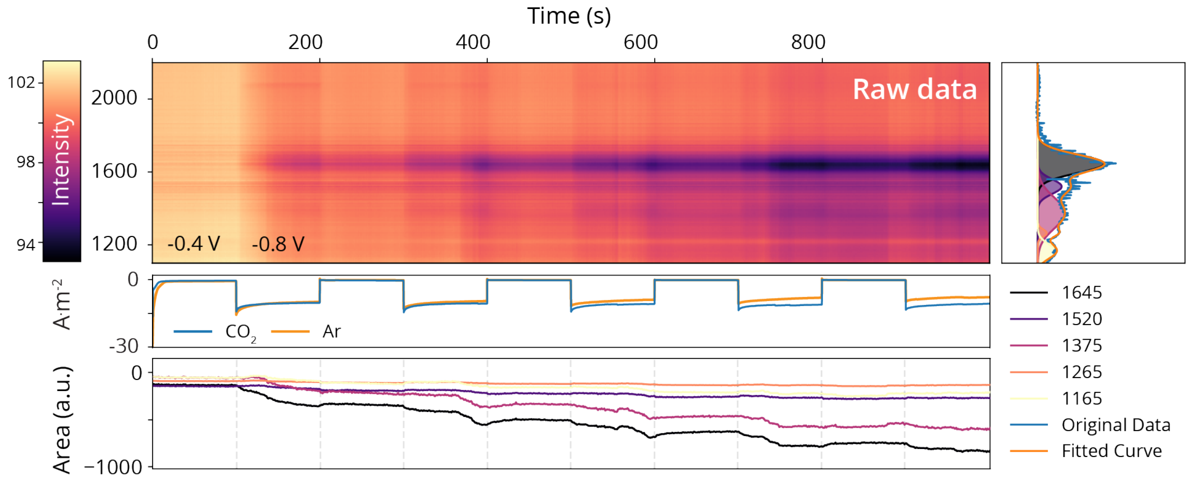

Figure S16: Time-resolved ATR-SEIRAS spectra in the carbonate region (2200-1100 cm^{-1}) plotted as 2D maps during potential-pulsed CO_2RR experiments in 0.2 M $NaHCO_3$ electrolyte saturated with CO_2. Potential pulsing was applied between -0.4 and -0.8 V_{RHE}. Curve fitting was applied based on the visible peaks, as shown in the representative spectrum on the right, and the area evolution of the fitted Gaussians over time is displayed at the bottom, as well as the recorded current densities during the performance of both the experiment with a CO_2-saturated solution and the control experiment with the Ar-saturated solution.

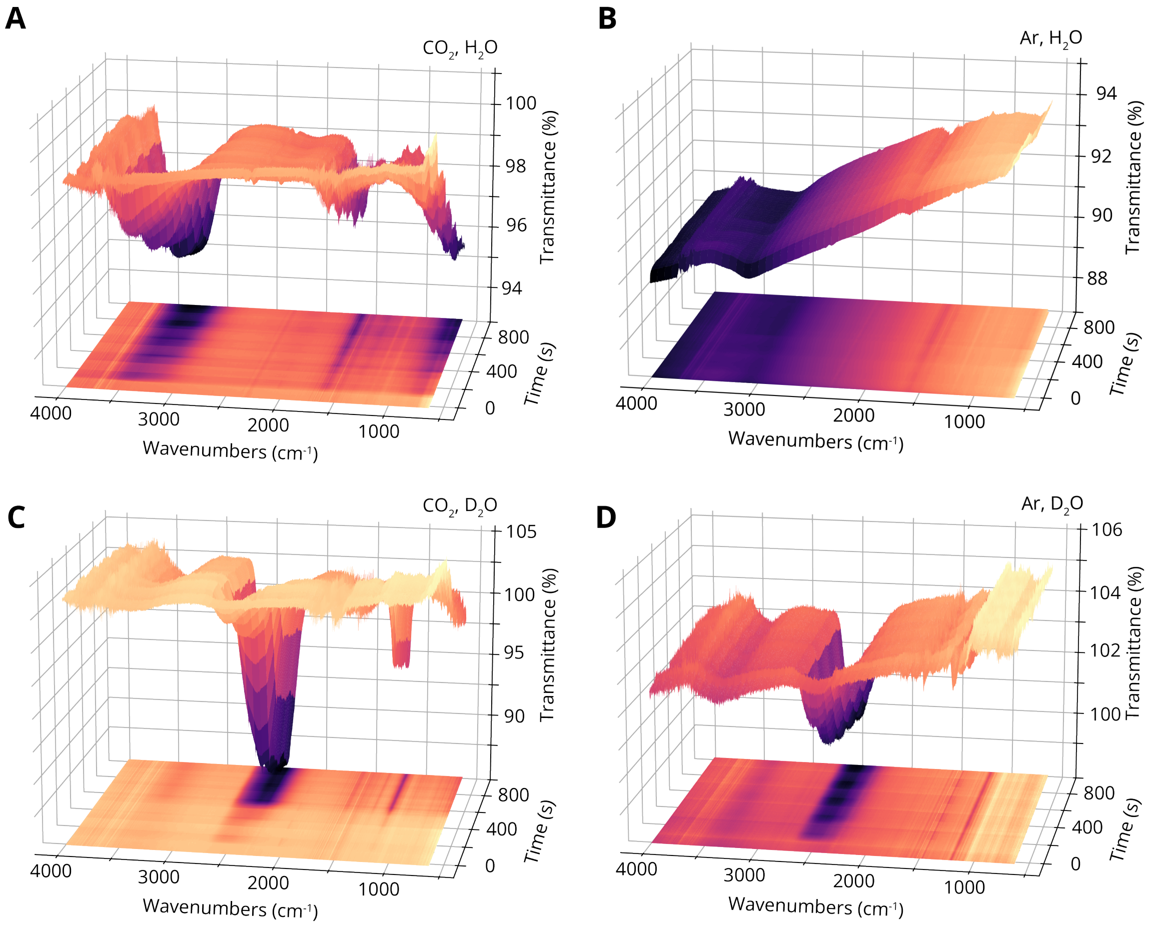

Figure S17: Raw datasets of the electrochemical ATR-SEIRAS operando experiments where CO_2RR over polycrystalline copper in 0.2 M $NaHCO_3$ electrolyte was performed using A) CO_2-saturated H_2O, B) Ar-saturated H_2O, C) CO_2-saturated D_2O, and D) Ar-saturated D_2O, by alternating the potentials between -0.4 and -0.8 V_{RHE} during 100 s along 5 cycles.

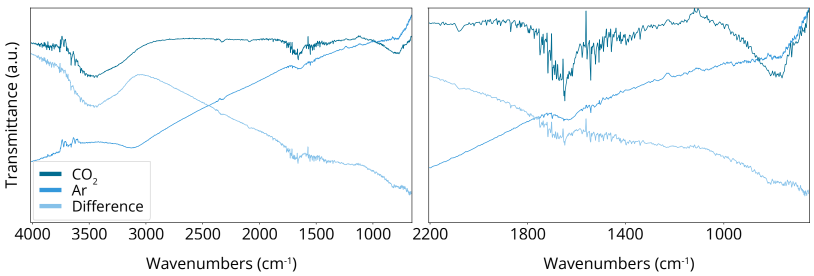

Figure S18: FTIR spectra of the experimental results employing CO_2- and Ar-saturated $NaHCO_3$ in H_2O solutions, as well as their respective difference, during the performance of CO_2RR the experiments, where the potentials was alternated between -0.4 and -0.8 V_{RHE} during 100 s along 5 cycles.

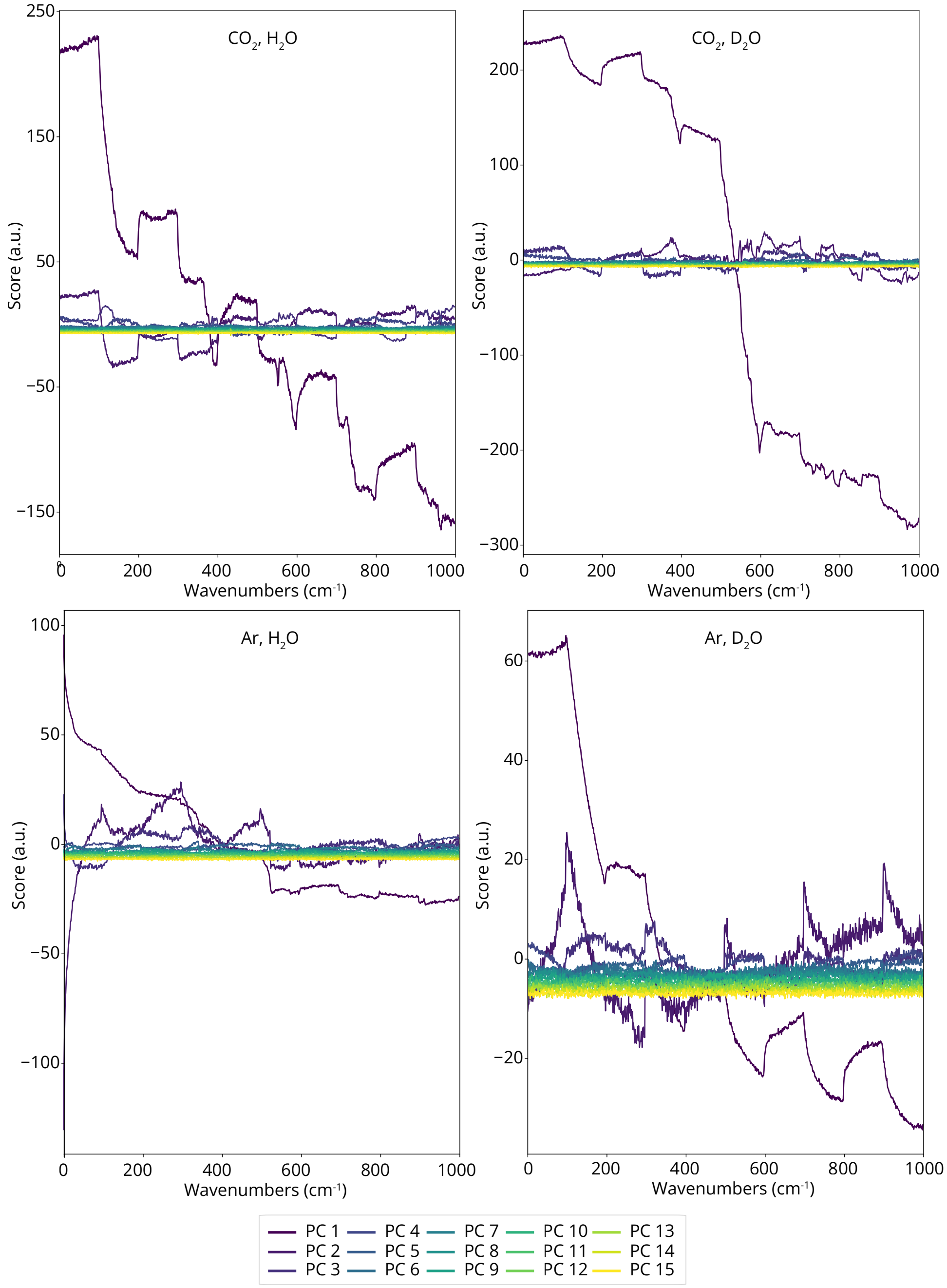

Figure S19: Scoreplots resulting from the eigendecomposition of the covariance matrix calculated from the mean-centered data shown in Figure S17.

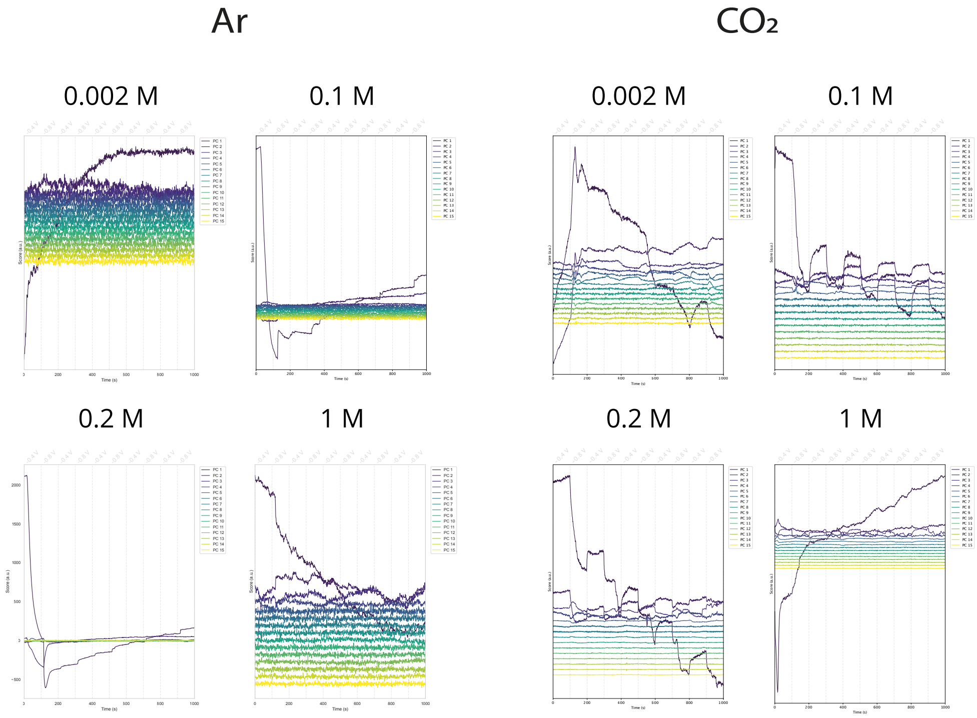

Figure S20: Scoreplots resulting from the eigendecomposition of the spectroscopic data derived from the use of $NaHCO_3$ at different concentrations (0.002 M, 0.1 M, 0.2 M and 1 M) while alternating the potential between -0.4 and -0.8 V_{RHE} using CO_2 and Ar-saturated solutions.

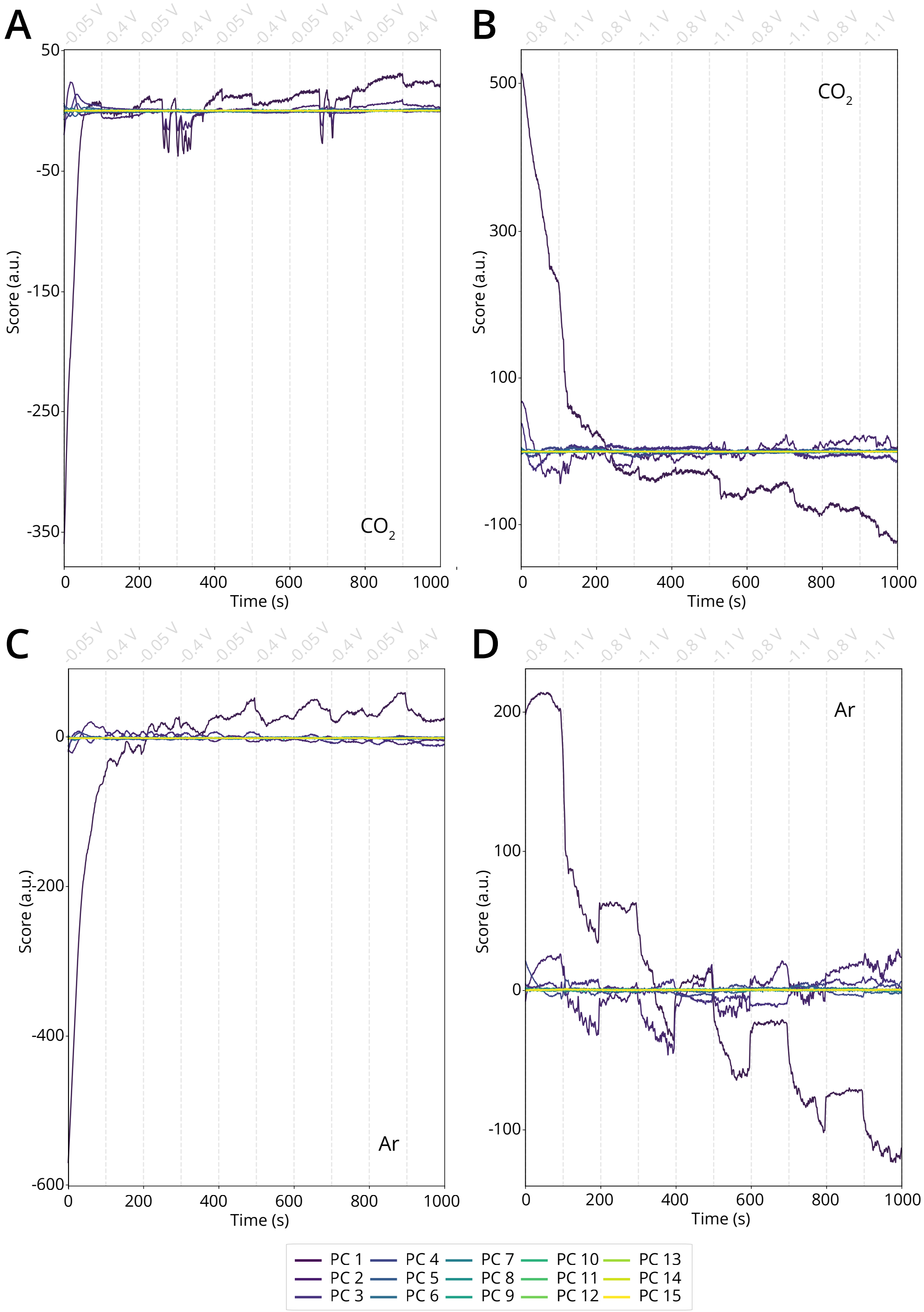

Figure S21: Scoreplots resulting from the eigendecomposition of the spectroscopic data derived from the use of $NaHCO_3$ 0.2 M while alternating the potential in CO_2-saturated solutions between A) -0.05 to -0.4 V_{RHE} and B) -0.8 to -1.1 V_{RHE}, as well as in Ar-saturated solutions between C) -0.05 to -0.4 V_{RHE} and D) -0.8 to -1.1 V_{RHE}.

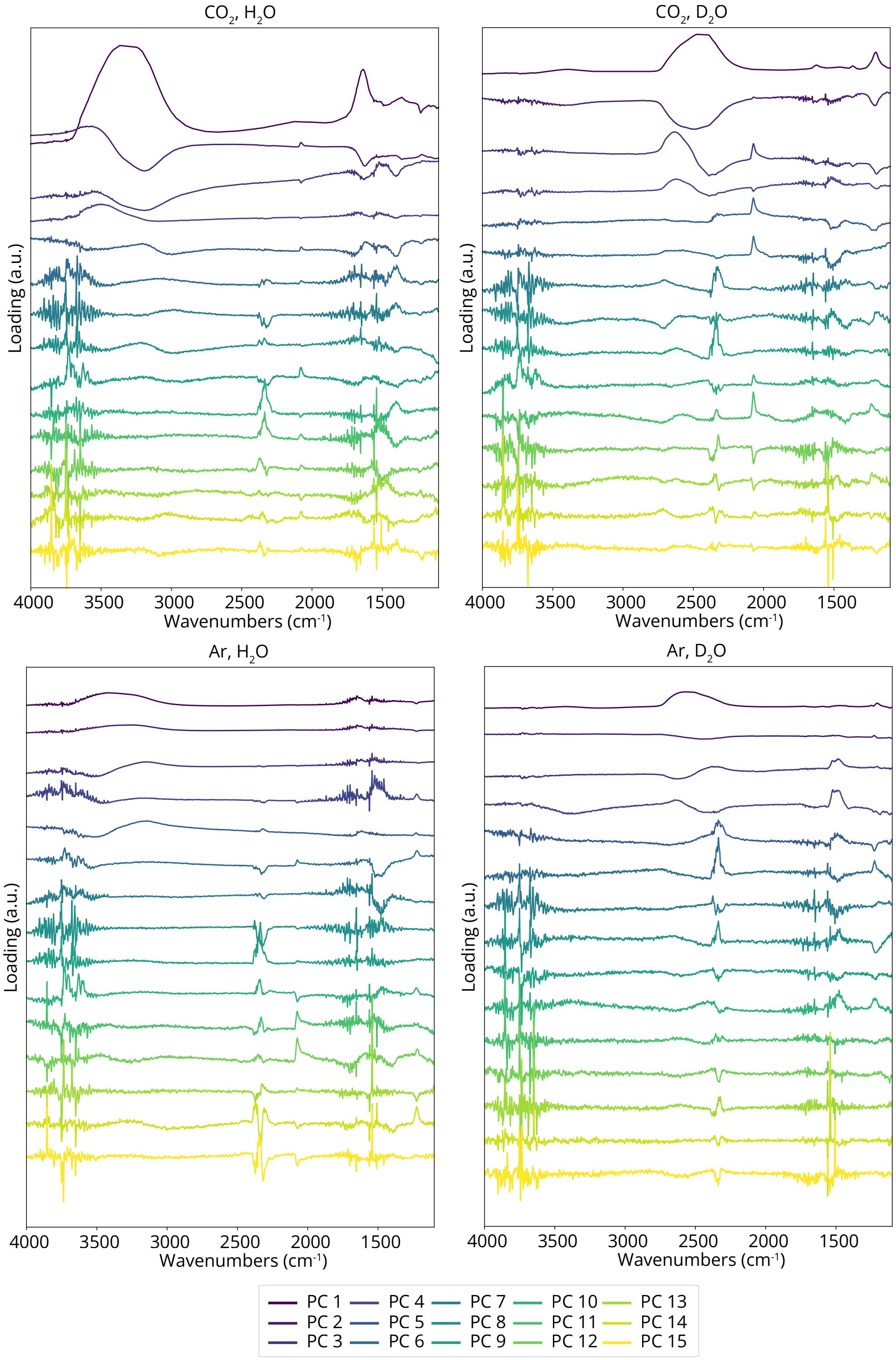

Figure S22: Eigenspectra resulting from the eigendecomposition of the covariance matrix calculated from the mean-centered data shown in Figure S17.

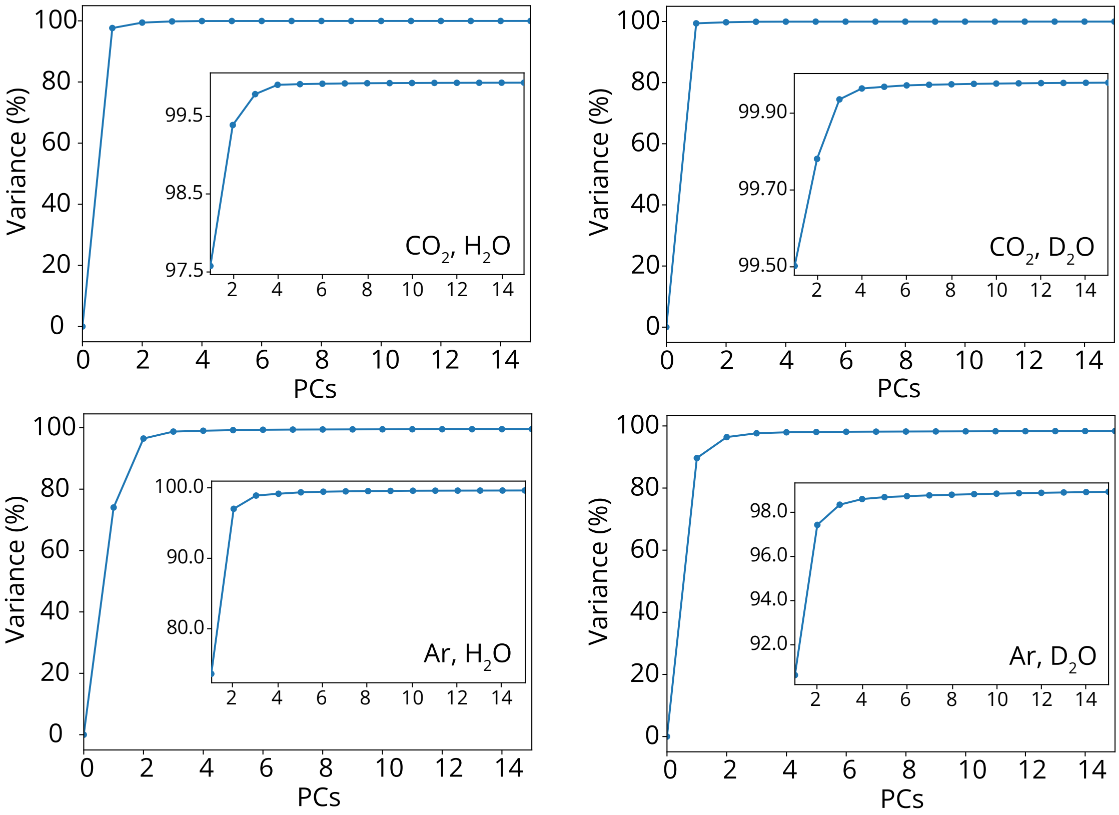

Figure S23: Cumulative variance explained (CVE) by the first 15 principal components resulting from the eigendecomposition of the covariance matrix calculated from the mean-centered data shown in Figure S17.

The *CO peak appears throughout PCs 2-15, which could indicate that the procedure incorrectly separates a shifting peak into several different PCs. To further understand these trends, PCA of only the 2100-2200 cm^{-1} region was performed for both the CO_2RR performed in H_2O, and D_2O as shown in Figures S24 and S25, respectively. The trends for both figures are similar, where the majority of the signal (above 99.9) of that region is caused by the combination overtone of water in PC1, followed by the *CO peak in PC2 responsible for roughly 0.04% of variance explained. There is a redshift of roughly 6 wavenumbers comparing the position of the *CO peak between H_2O, and D_2O. Furthermore time responses noted indicate that the initially adsorbed species of CO for both electrolytes is at higher wavenumbers, and that there is likely a contribution of $CO_{(aq)}$ in PC3. Overall, this analysis shows that the reason that *CO shows up in so many PCs when the entire spectral range is analyzed, is due to its correlation with other species, rather than a gradual shift being misclassified.

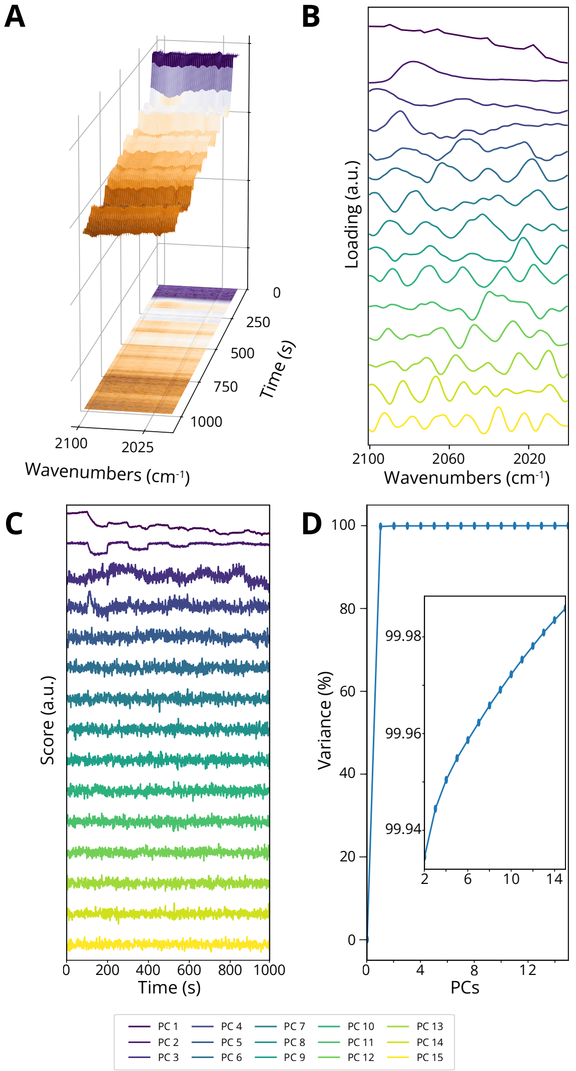

Figure S24: A) Raw time-series evolution of FTIR spectra, B) eigenspectra, C) scoreplots, and D) cumulative variance explained derived from the application of PCA onto the 2100-to-2000 cm^{-1} window obtained from the CO_2RR results during the application of alternating pulses at -0.4 and -0.8 V_{RHE} in a CO_2-saturated solution in H_2O.

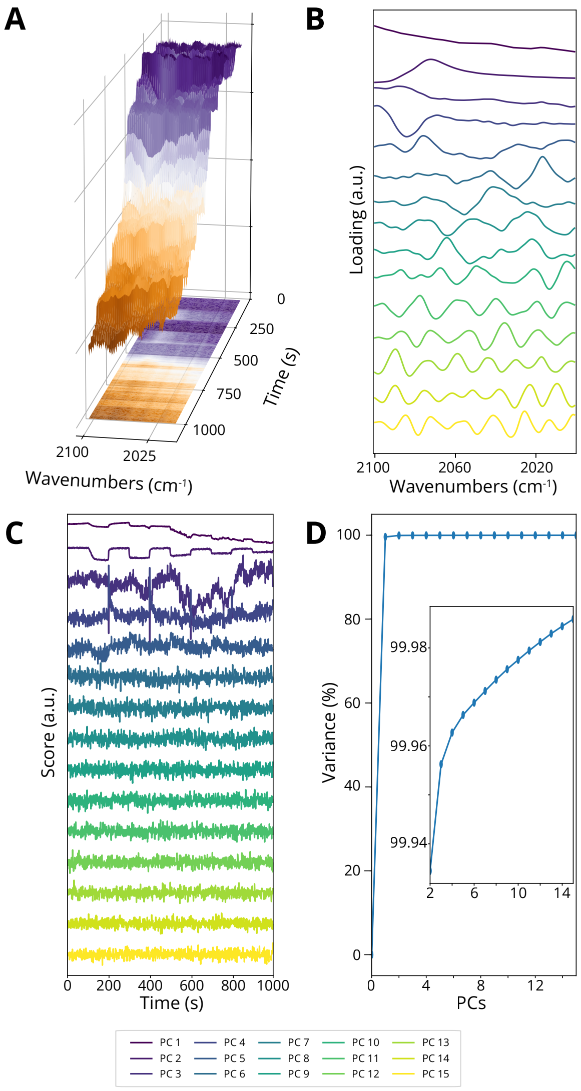

Figure S25: A) Raw time-series evolution of FTIR spectra, B) eigenspectra, C) scoreplots, and D) cumulative variance explained derived from the application of PCA onto the 2100-to-2000 cm^{-1} window obtained from the CO_2RR results during the application of alternating pulses at -0.4 and -0.8 V_{RHE} in a CO_2-saturated solution in D_2O.

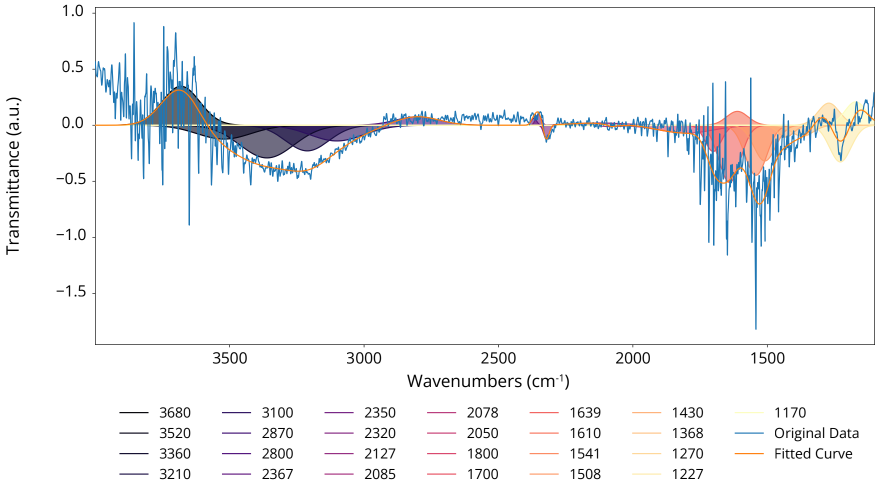

Figure S26: ATR-SEIRAS spectra at OCP before the performance of CO_2RR over Cu in 0.2 M $NaHCO_3$ electrolyte in H_2O saturated with CO_2 in the range of 4000-1100 cm^{-1}. Gaussian fits were performed, where the center of the Gaussian curves is based on the presence of patterns displayed in the eigenspectra shown in Figure S22, and their corresponding assignation is specified in Table S2.

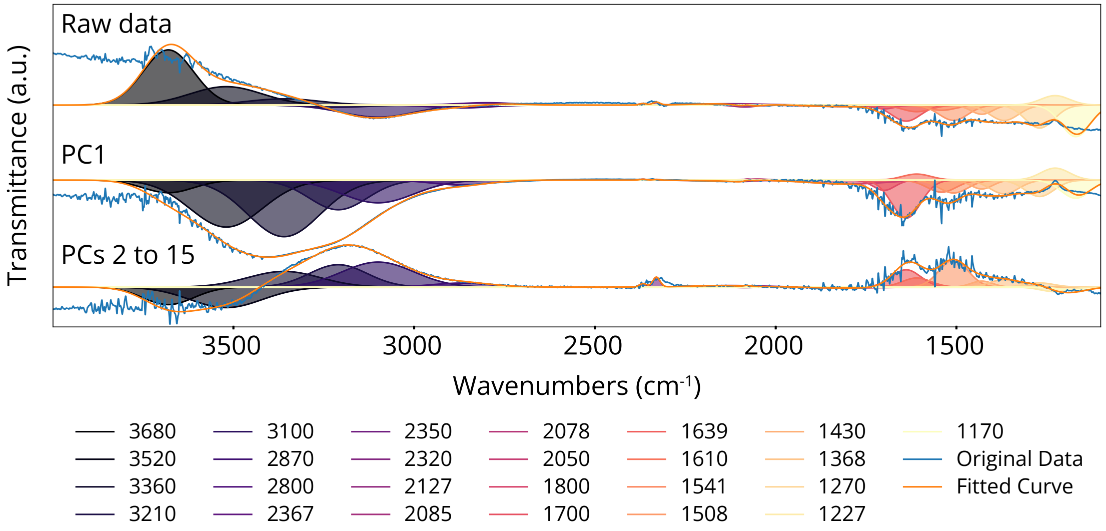

Figure S27: Gaussian-fits applied onto a representative spectrum obtained from the raw dataset of a standard CO_2RR experiment using a 0.2 M $NaHCO_3$ Ar-saturated solution in H_2O, as well as the reconstructed datasets using PCs 1 and 2-15 derived from the application of PCA. The center of the Gaussian curves is based on the presence of patterns displayed in the eigenspectra shown in Figure S22, and their corresponding assignation is specified in Table S2.

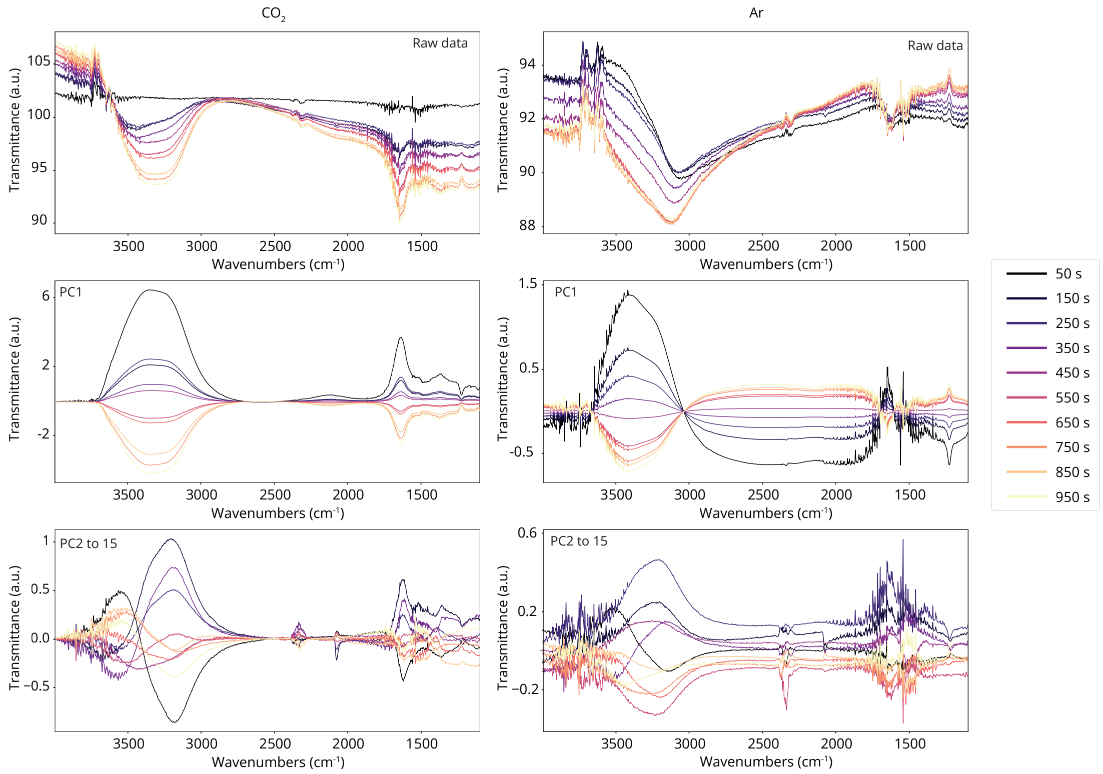

Figure S28: ATR-SEIRAS spectra during potential-pulsed CO_2RR experiments in 0.2 M $NaHCO_3$ electrolyte in H_2O, saturated with CO_2 (left), and Ar (right). Potential pulsing was applied between -0.4 and -0.8 V_{RHE}. Single spectra from the middle of each pulse of the raw data, as well as the reconstructed data matrices from PC1, and PCs 2-15 are shown.

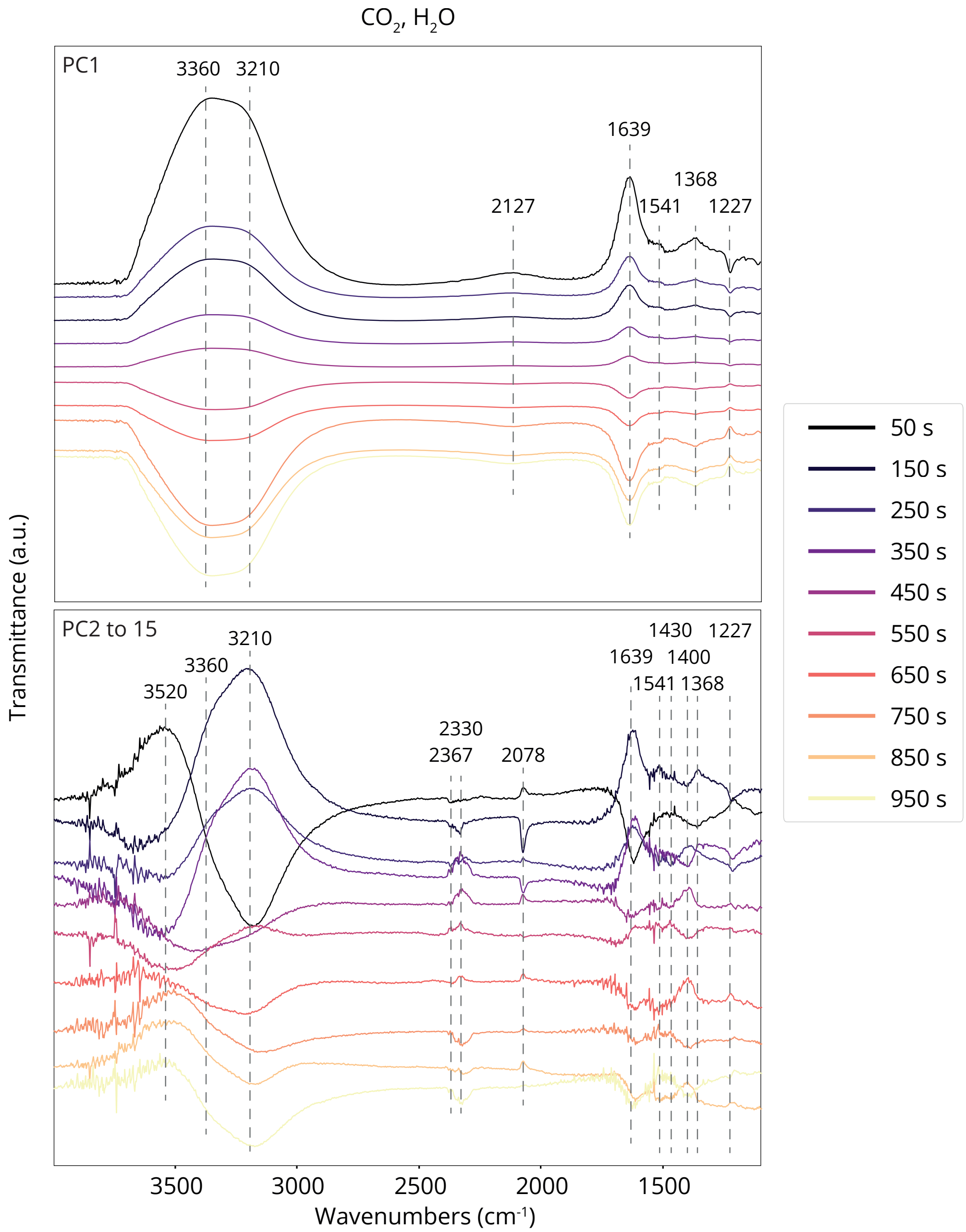

Figure S29: ATR-SEIRAS reconstructed spectra from Figure S28 during potential-pulsed CO_2RR experiments in 0.2 M $NaHCO_3$ in electrolyte H_2O, saturated with CO_2, with indications of peak positions.

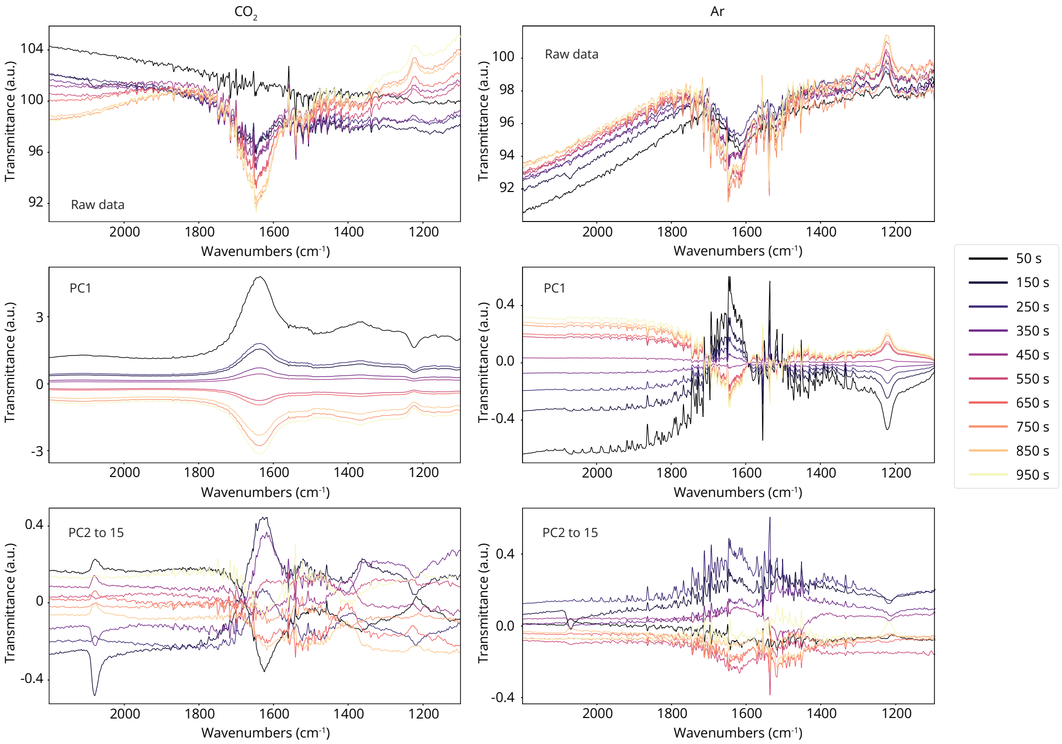

Figure S30: ATR-SEIRAS spectra in the carbonate region (2200-1100 cm^{-1}) during potential-pulsed experiments in 0.2 M $NaHCO_3$ electrolyte in H_2O, saturated with CO_2 (left), and Ar (right). Potential pulsing was applied between -0.4 and -0.8 V_{RHE}. Single spectra from the middle of each pulse of the raw data, as well as the reconstructed data matrices from PC1,and PCs 2-15 are shown.

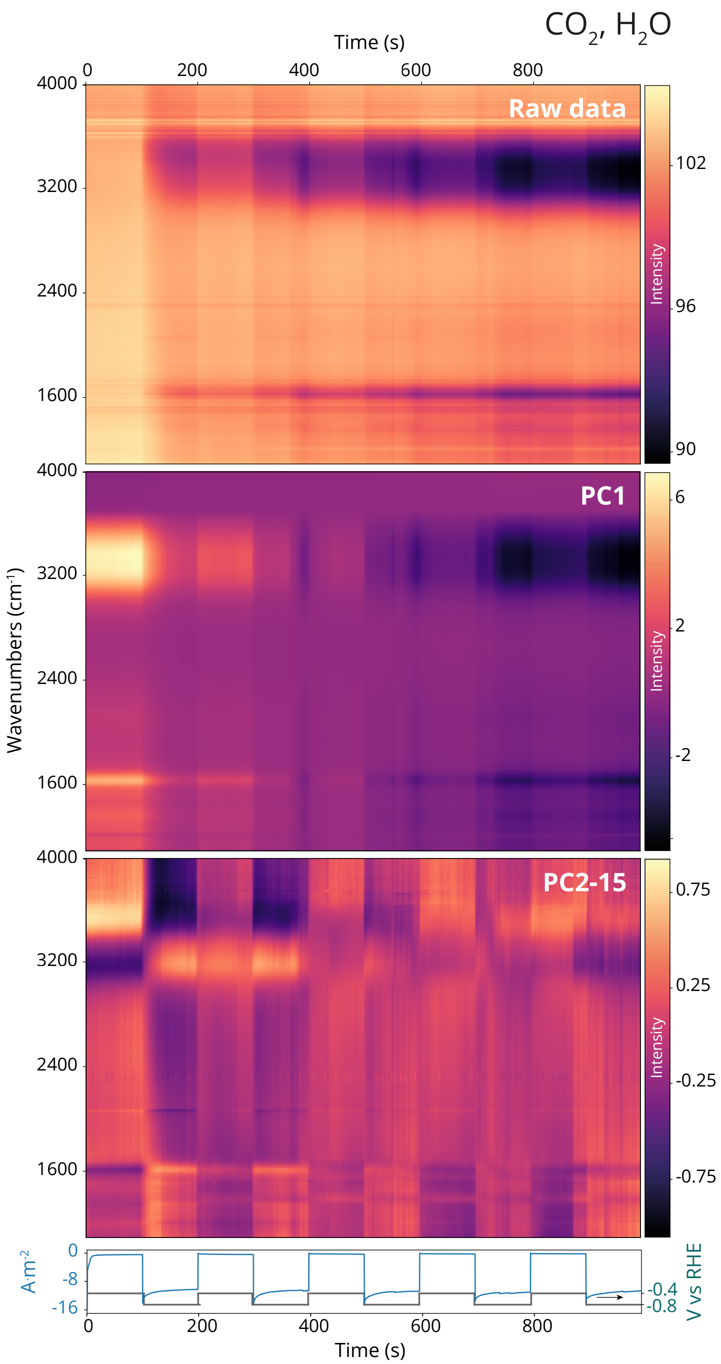

Figure S31: Time-resolved ATR-SEIRAS spectra plotted as heatmaps during potential-pulsed experiments in 0.2 M $NaHCO_3$ electrolyte in H_2O, saturated with Ar. Potential pulsing was applied between -0.4 and -0.8 V_{RHE}. The raw data, as well as the reconstructed data matrices from PC1 and PCs 2-15 are shown, and the recorded current density aligned with the spectral data.

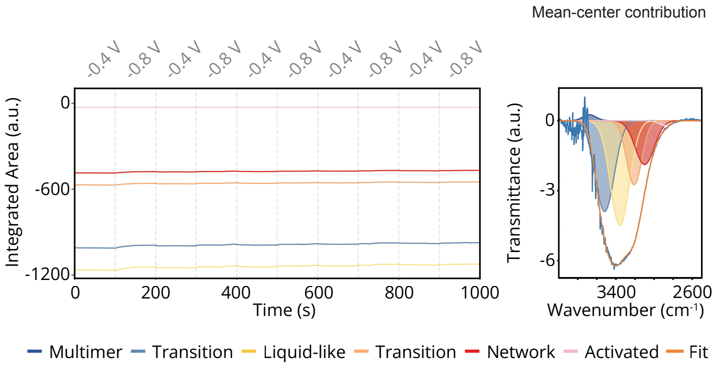

Figure S32: Profiles of the areas in the $\nu(H_2O)$ region of the 6 Gaussian peaks based on the peaks found in the eigenspectra (3680, 3520, 3360, 3210, 3100, and 2890 cm^{-1}, with standard deviations 70, 100, 100, 80, 100, 60 cm^{-1}) used to fit the mean-center contribution, obtained from the reconstruction of PC1 from the non mean-centered data results using a CO_2-saturated 0.2 M $NaHCO_3$ in H_2O solution.

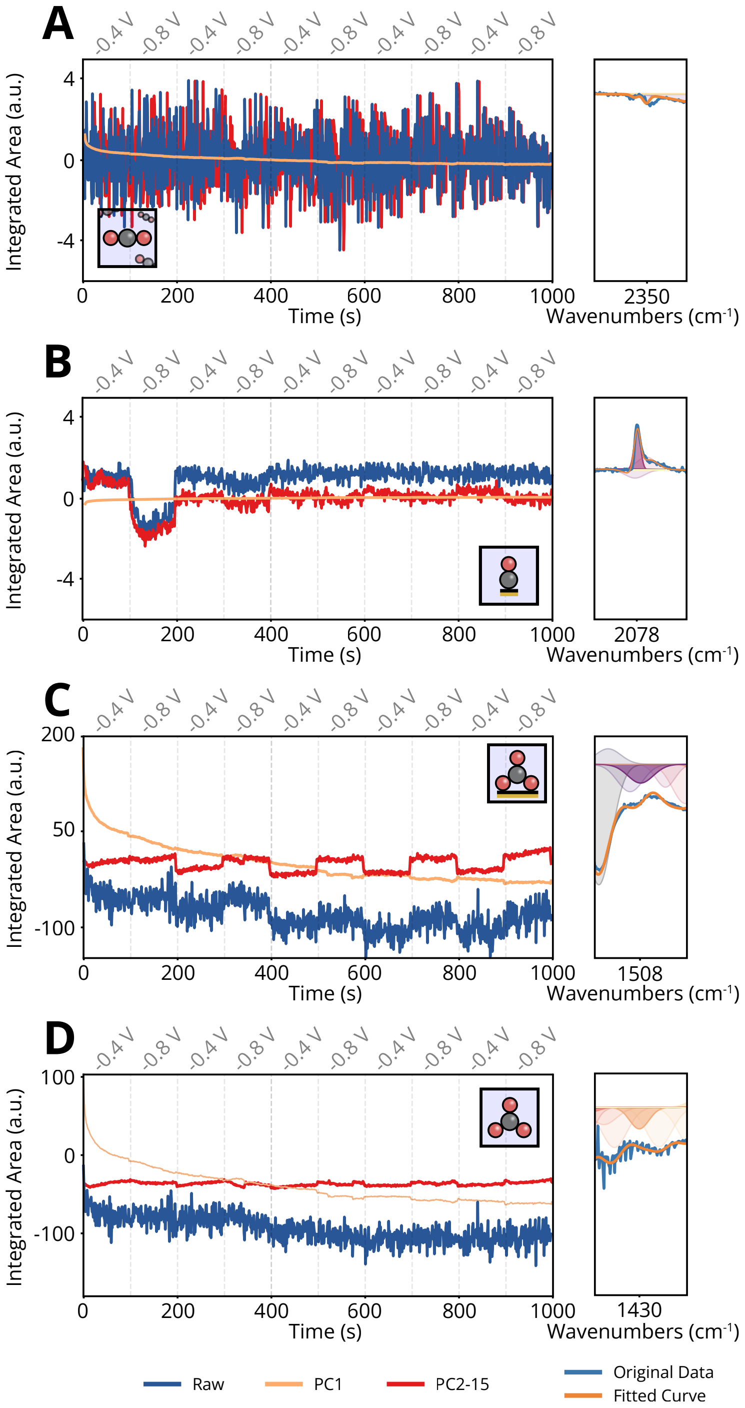

Figure S33: Evaluation of the integrated area over time of the Gaussian peaks fitted in the raw data matrix during the application of alternating pulses at -0.4 and -0.8 V_{RHE} using an Ar-saturated 0.2 M $NaHCO_3$ solution, as well as the reconstructed data matrices using PC1 and PCs2-15, at A) 2350 cm^{-1} ($CO_{2(aq)}$), B) 2078 cm^{-1} (*CO), C) 1508 cm^{-1} (*CO_3^{2-}), and D) 1430 cm^{-1} (CO_3^{2-}).

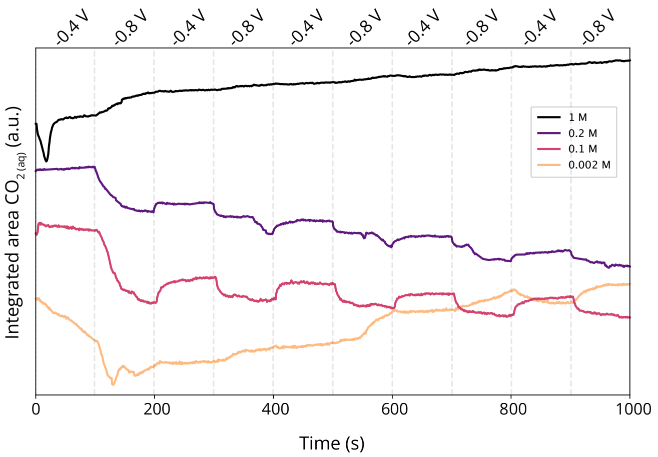

Figure S34: Evaluation of the integrated area over time of the Gaussian peaks fitted in the reconstructed data matrices using PC1 during the application of alternating pulses at -0.4 and -0.8 V_{RHE} using an CO_2-saturated $NaHCO_3$ solutions at 0.002 M, 0.1 M, 0.2 M and 1 M concentrations 2350 cm^{-1} ($CO_{2(aq)}$)

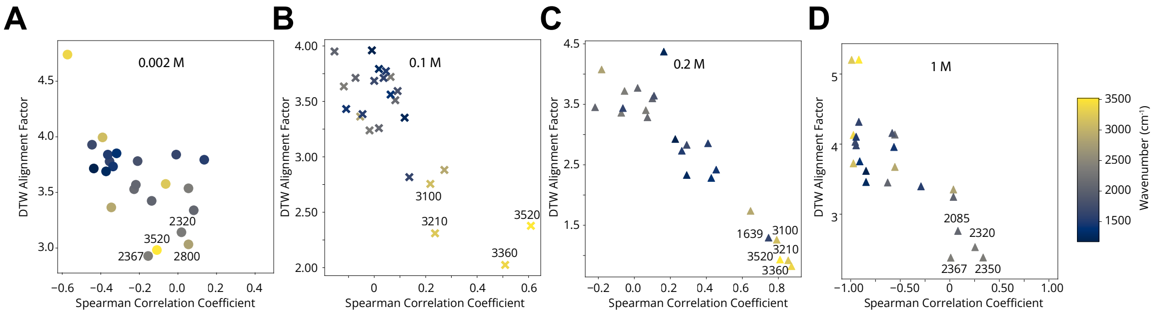

Figure S35: Scatterplots showing the correlation between the obtained DTW alignment costs and the Spearman coefficients relative to the integrated areas in the raw dataset at different wavenumbers after the performance of a standard experiment employing CO_2-saturated solutions of $NaHCO_3$ at 0.002 M (A), 0.1 M (B), 0.2 M (C), and 1 M (D) while applying alternating electrochemical pulses at -0.4 and -0.8 V_{RHE}.

S4.1.2 Isotopically Labelled Experiments, Copper Catalyst

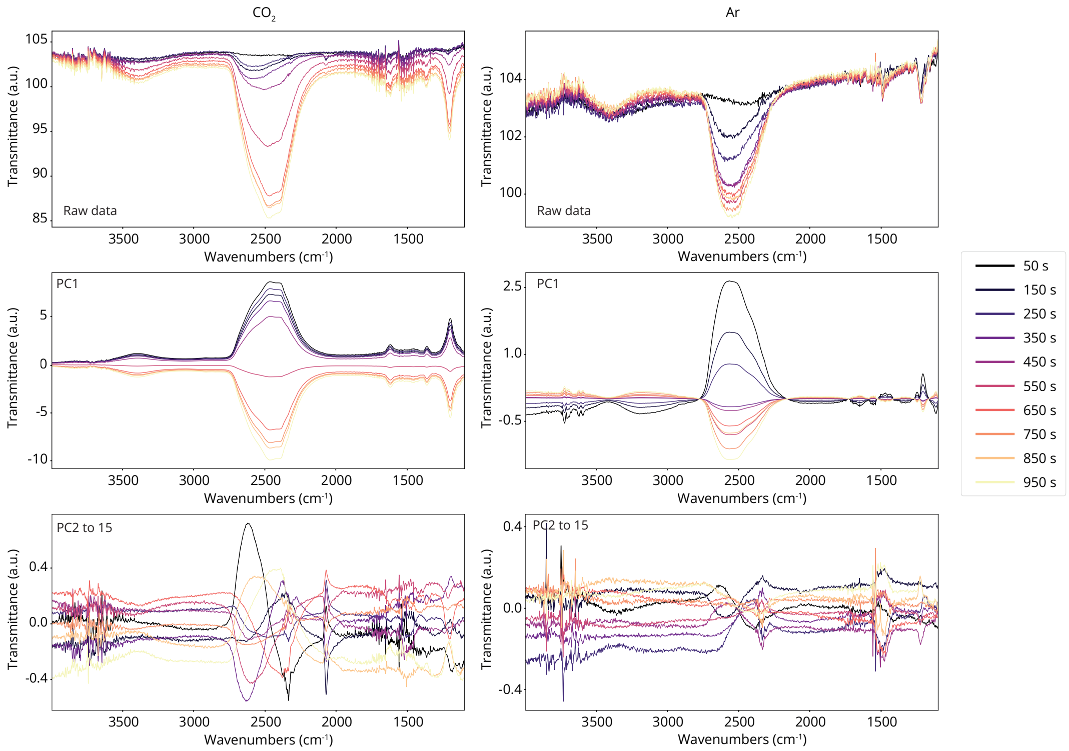

Figure S36: ATR-SEIRAS spectra in the carbonate region (2200-1100 cm^{-1}) during potential-pulsed CO_2RR experiments in 0.2 M $NaHCO_3$ electrolyte in D_2OO, saturated with CO_2 (left), and Ar (right). Potential pulsing was applied between -0.4 and -0.8 V_{RHE}. Single spectra from the middle of each pulse of the raw data, as well as the reconstructed data matrices from PC1, and PCs 2-15 are shown.

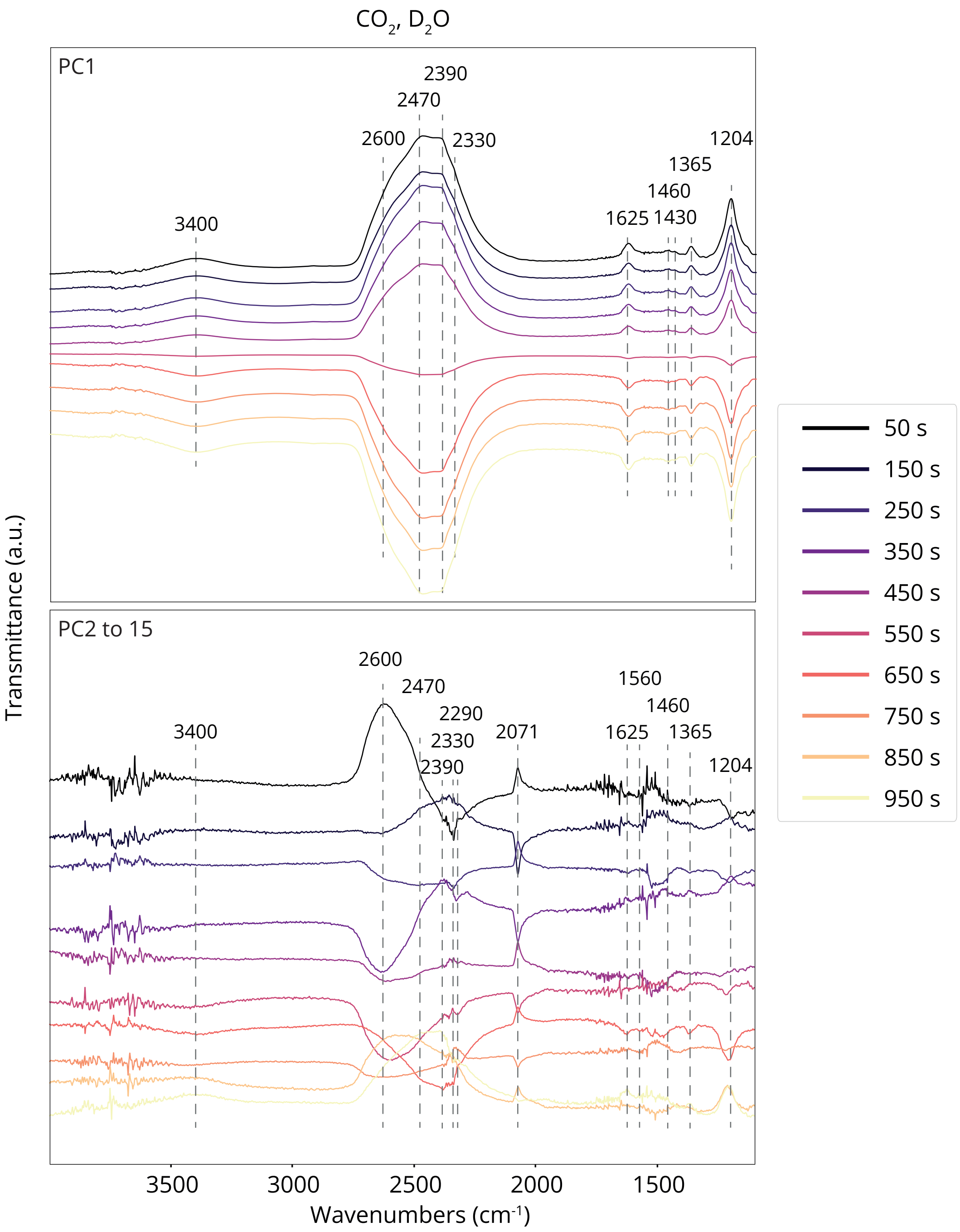

Figure S37: ATR-SEIRAS spectra from Figure S36 during potential-pulsed CO_2RR experiments in 0.2 M $NaHCO_3$ in electrolyte D_2OO, saturated with CO_2, with indications of peak positions.

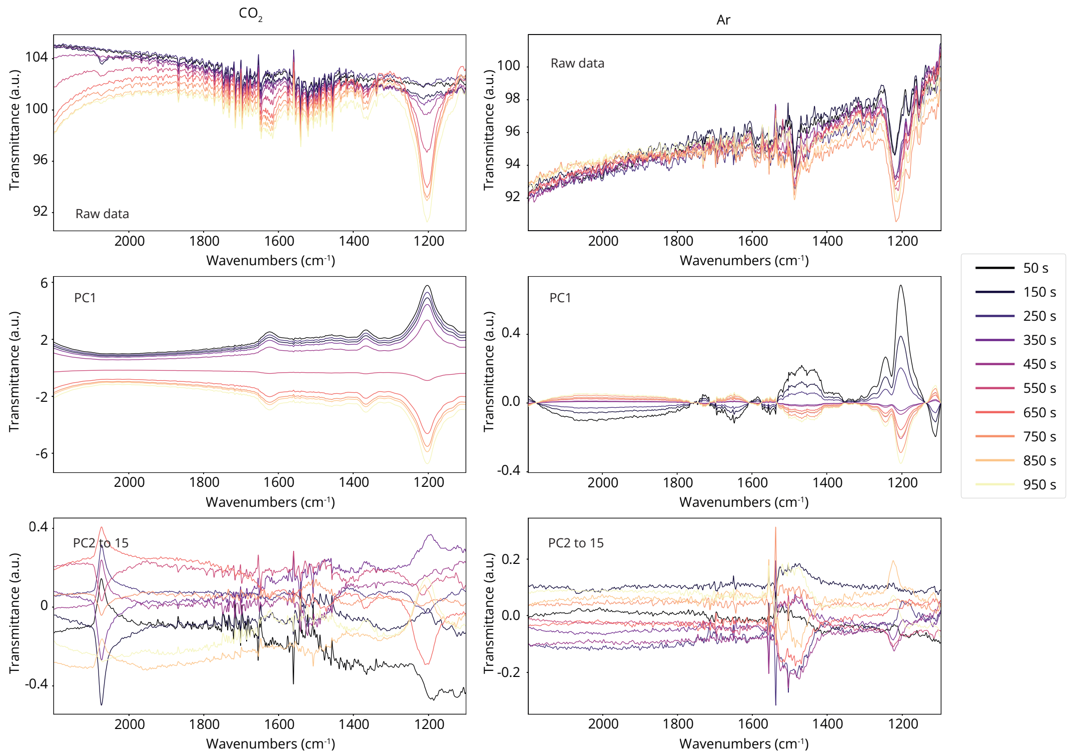

Figure S38: ATR-SEIRAS spectra in the carbonate region (2200-1100 cm^{-1}) during potential-pulsed CO_2RR experiments in 0.2 M $NaHCO_3$ electrolyte in D_2O, saturated with CO_2 (left), and Ar (right). Potential pulsing was applied between -0.4 and -0.8 V_{RHE}. Single spectra from the middle of each pulse of the raw data, as well as the reconstructed data matrices from PC1, and PCs 2-15 are shown.

S4.1.3 Gold Catalyst

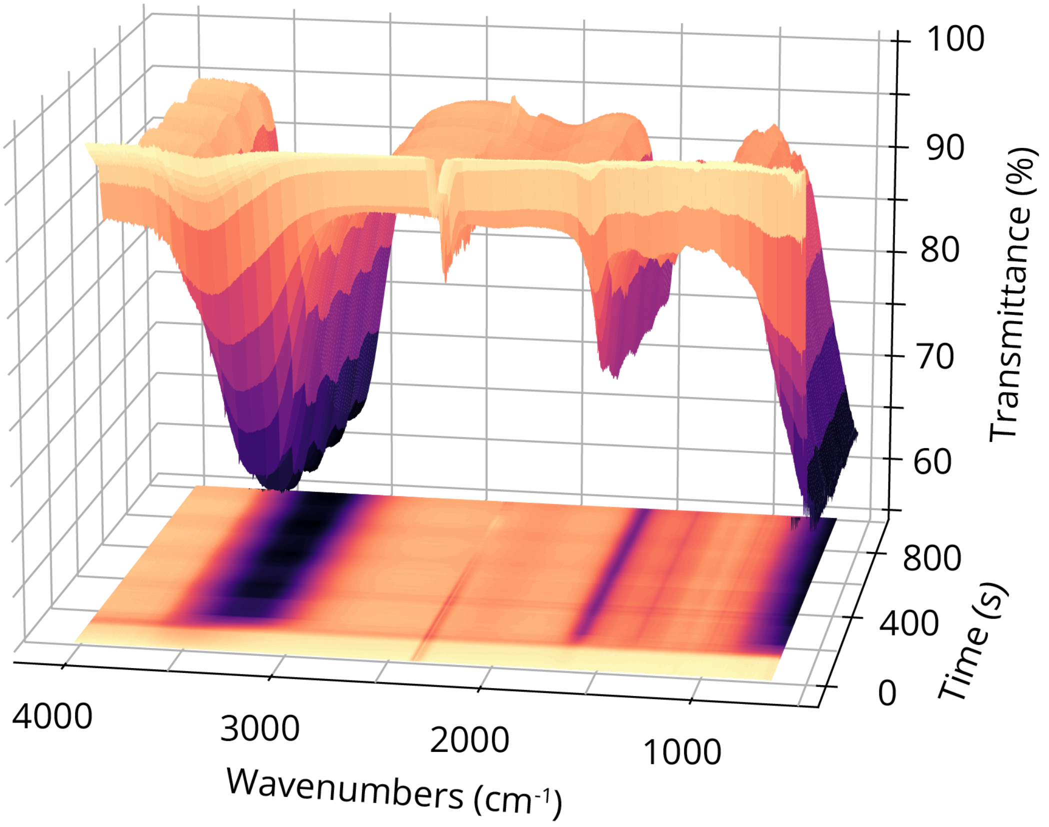

Figure S39: Raw datasets of the electrochemical ATR-SEIRAS operando experiments where CO_2RR over polycrystalline gold in 0.2 M $NaHCO_3$ electrolyte was performed using CO_2-saturated H_2O, by alternating the potentials between -0.4 and -0.8 V_{RHE} during 100 s along 5 cycles.

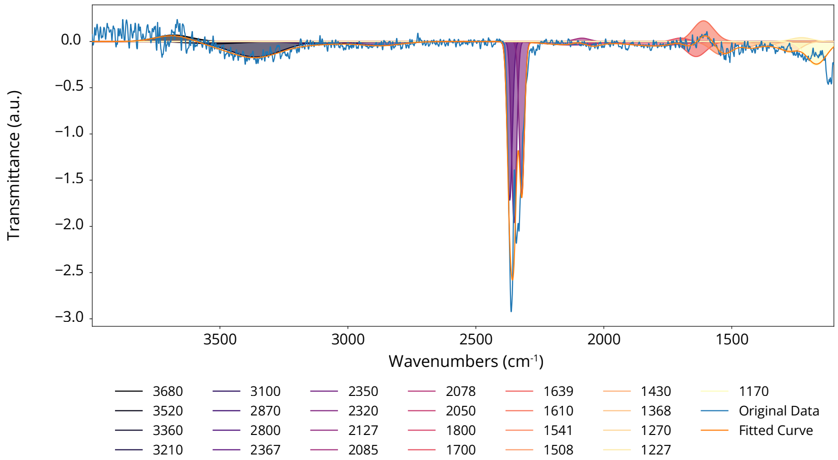

Figure S40: ATR-SEIRAS spectra at OCP before the performance of CO_2RR over Au in 0.2 M $NaHCO_3$ electrolyte in H_2O saturated with CO_2 in the range of 4000-1100 cm^{-1}. Gaussian fits were performed, where the center of the Gaussian curves is based on the presence of patterns displayed in the eigenspectra shown in Figure S22, and their corresponding assignation is specified in Table S2.

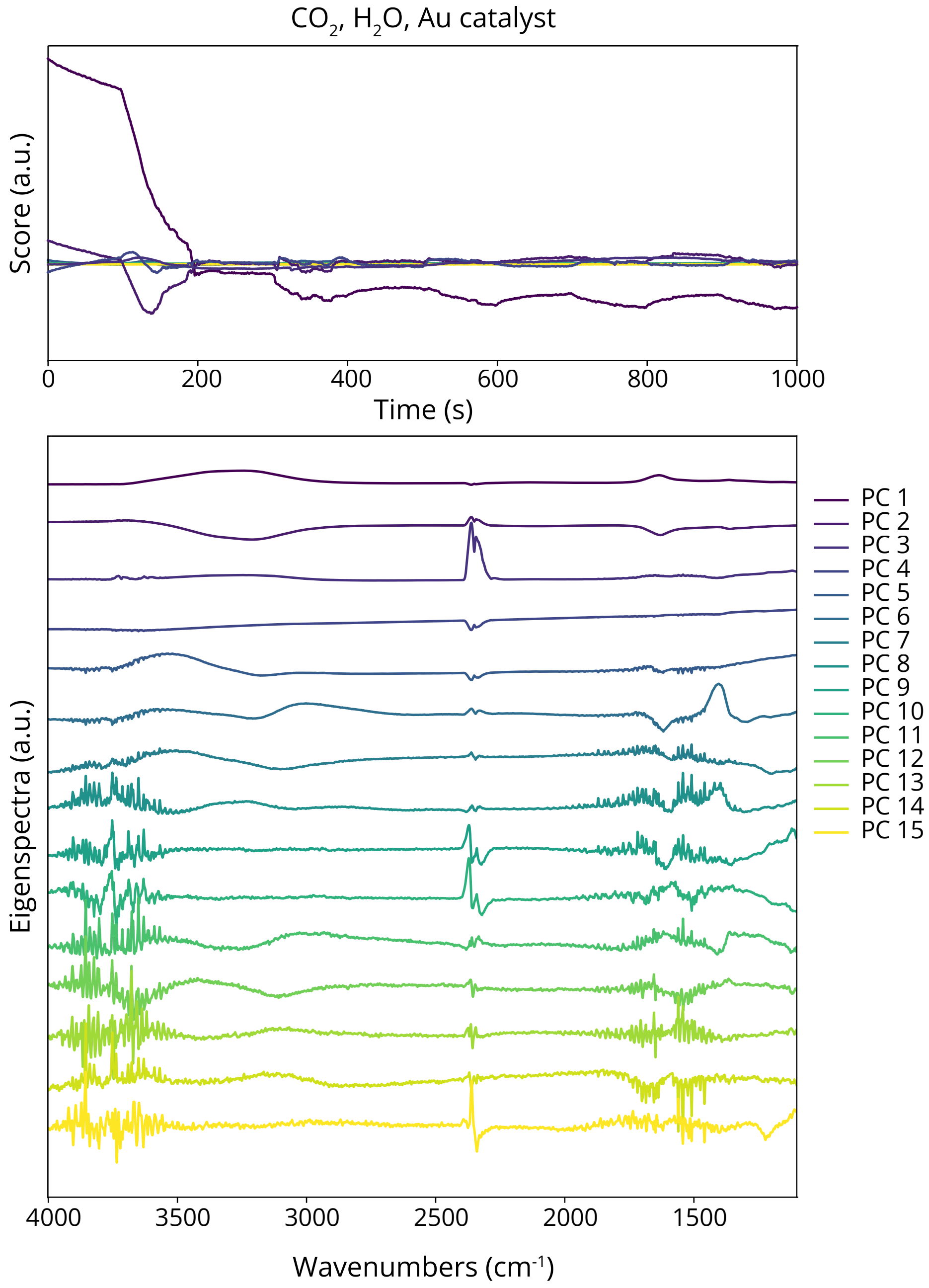

Figure S41: Scoreplots (upper) and eigenspectra (lower) resulting from the performance of PCA onto the results derived from the CO_2RR experiments using a CO_2-saturated solution of $NaHCO_3$ 0.2 M and a polycrystalline gold layer as catalyst during the application of alternating pulses at -0.4 and -0.8 V_{RHE} for 100 s along 5 cycles.

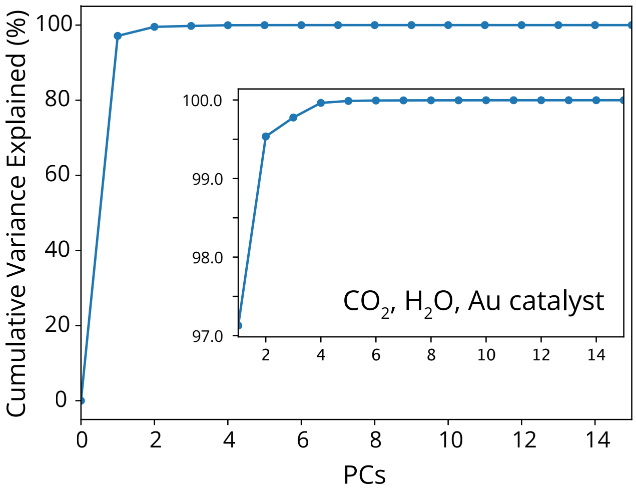

Figure S42: Cumulative variance explained per PC resulting from the performance of PCA onto the results derived from the CO_2RR experiments using a CO_2-saturated solution of $NaHCO_3$ 0.2 M and a polycrystalline gold layer as catalyst during the application of alternating pulses at -0.4 and -0.8 V_{RHE} for 100 s along 5 cycles.

S4.2 Electrode Characterization

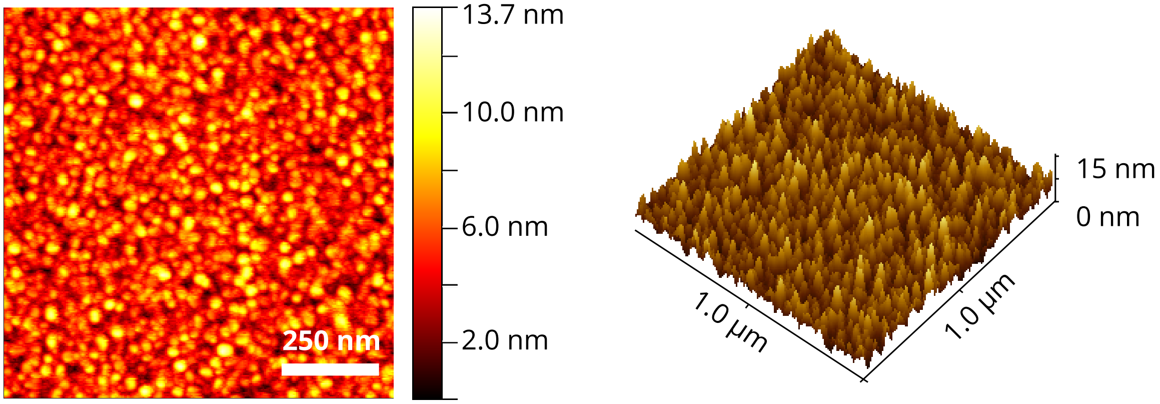

Figure S43: Atomic force micrograph showing the topology of the polycrystalline Cu electrode prepared by e-beam evaporation.

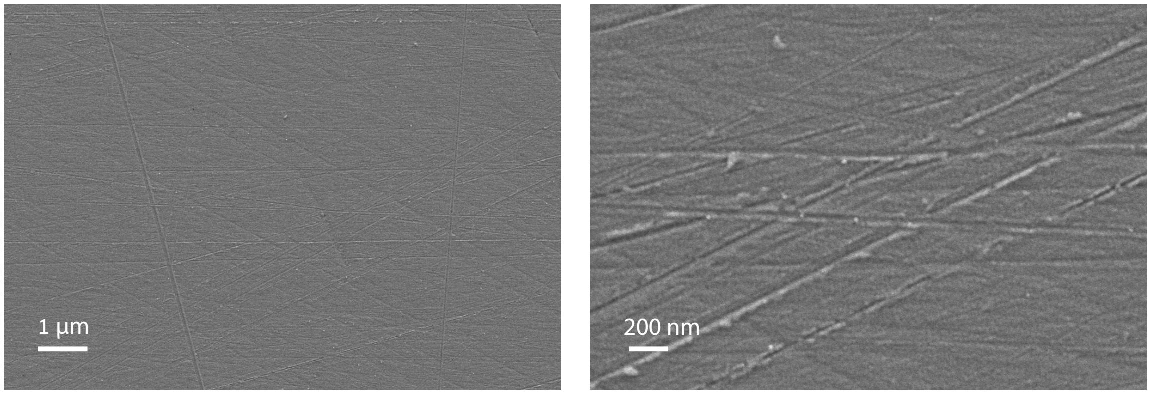

Figure S44: SEM image (top-view) of the IREs after Cu deposition via e-beam evaporation.

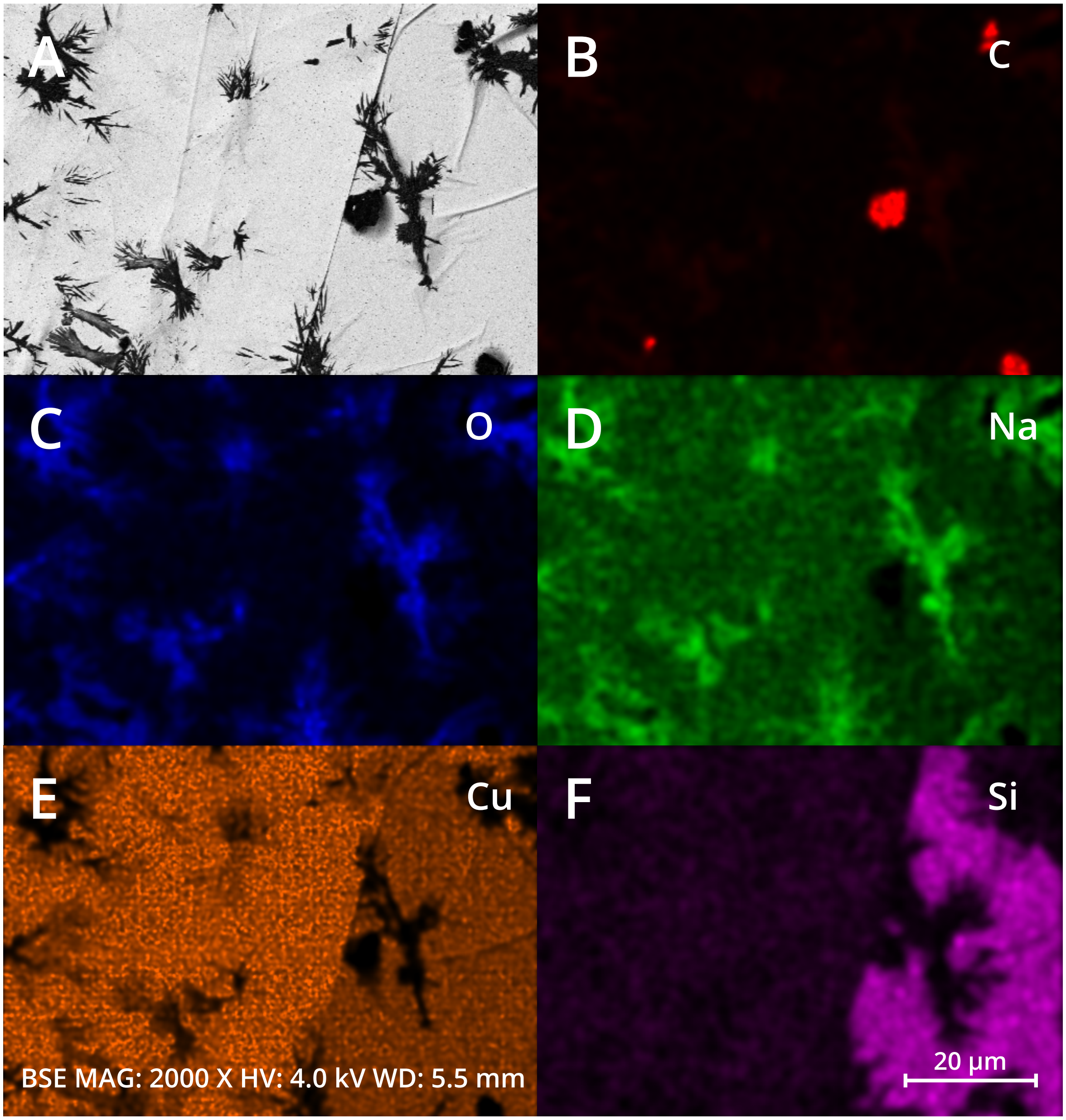

Figure S45: SEM image of Cu-deposted wafers after the performace of the electrochemical experiments (A), along with the corresponding C (B), O (C), Na (D), Cu (E), and Si (F) distribution maps, obtained via EDS at 4.0 kV.

S4.3 Determination potential of zero free charge

The potential of zero free charged is expected to influence the CO_2RR and the EDL, as the application of electrochemical potentials far from it entails higher kinetic energy barriers for the reaction to occur,[14] as a result of the higher activation energy barriers associated with phase transitions. To investigate the reorganization of the EDL around the potential of zero free charge (pzfc), i.e., the potential at which the metal surface has zero excess electronic charge in our system, capacitance curves were obtained through cyclic voltammetry by dividing the current density by the scan rate (Figure S46. For these experiments, the potential was swept between 0.3 and -0.4 V_{RHE} at a scan rate of 0.01 V/s using the same system as the one described in section S1.1.4. These experiments

were aimed at determining the minimum differential capacitance (C_d) expected to be encountered at the pzfc, following the Gouy-Chapman theory.[109] To prevent the interference of Helmholtz capacitance, the experiments were conducted at low $NaHCO_3$ concentrations, 0.002 M.

As observed in Figure S46, such minima were found at ~ -0.25 V_{RHE}, in the order of previous experimental results reported in literature.[14,110,111]

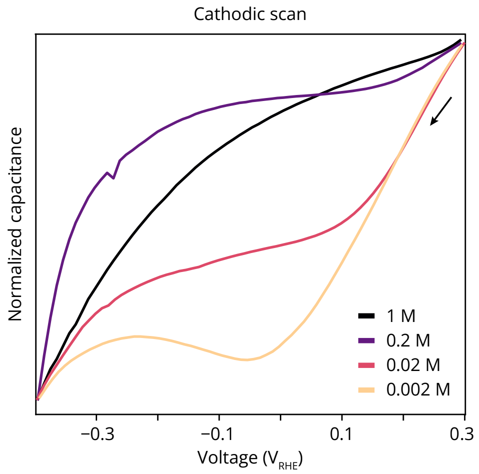

Figure S46: Capacitance curves (cathodic scan) obtained from CO_2-saturated $NaHCO_3$ solutions between 0.3 and -0.4 V_{RHE} at 1 M, 0.2 M, 0.02 M and 0.002 M.

References

[1] Ibn Minhaj, T.; Annayev, M.; Adelegan, O. J.; Biliroğlu, A. Ö.; Yamaner, F. Y.; Oralkan, Ö. *Micromachines* **2024**, *15*, 1106.

[2] Hughes, M. A.; Brennan, P. M.; Bunting, A. S.; Shipston, M. J.; Murray, A. F. *Journal of Visualized Experiments* **2014**, 50929.

[3] Kamper, H. Dynamic Time Warping. https://github.com/kamperh/lecture_dtw_notebook, 2021.

[4] Mojet, B. L.; Ebbesen, S. D.; Lefferts, L. *Chemical Society Reviews* **2010**, *39*, 4643.

[5] Morhart, T. A.; Read, S. T.; Wells, G.; Jacobs, M.; Rosendahl, S. M.; Achenbach, S.; Burgess, I. J. *Analytical Methods* **2019**, *11*, 5776–5783.

[6] Kozuch, J.; Ataka, K.; Heberle, J. *Nature Reviews Methods Primers* **2023**, *3*, 70.

[7] Neubrech, F.; Huck, C.; Weber, K.; Pucci, A.; Giessen, H. *Chemical Reviews* **2017**, *117*, 5110–5145.

[8] Milosevic, M. *Applied Spectroscopy* **2013**, *67*, 126–131.

[9] Hollas, J. M. *Modern Spectroscopy*, 4th ed.; Wiley: Chichester, 2004; pp 74–75.

[10] Ramer, G.; Lendl, B. *Encyclopedia of Analytical Chemistry*; John Wiley & Sons, Ltd, 2013.

[11] Krischer, K.; Savinova, E. R. In *Handbook of Heterogeneous Catalysis*, 1st ed.; Ertl, G., Knözinger, H., Schüth, F., Weitkamp, J., Eds.; Wiley, 2008; pp 1873–1905.

[12] Ertl, G. G.; Knözinger, H.; Schüth, F.; Weitkamp, J. *Handbook of Heterogeneous Catalysis*; Wiley-VCH: Weinheim, 2008.

[13] She, Z. W.; Kibsgaard, J.; Dickens, C. F.; Chorkendorff, I.; Nørskov, J. K.; Jaramillo, T. F. *Science* **2017**, *355*, 1–12.

[14] Hou, J.; Xu, B.; Lu, Q. *Nature Communications* **2024**, *15*, 1926.

[15] Bhattacharyya, D.; Videla, P. E.; Cattaneo, M.; Batista, V. S.; Lian, T.; Kubiak, C. P. *Chemical Science* **2021**, *12*, 10131–10149.

[16] Waegele, M. M.; Gunathunge, C. M.; Li, J.; Li, X. *The Journal of Chemical Physics* **2019**, *151*, 160902.

[17] Wang, S.; Zhang, J.; Gharbi, O.; Vivier, V.; Gao, M.; Orazem, M. E. *Nature Reviews Methods Primers* **2021**, *1*, 41.

[18] Brückner, A. *Chem. Commun.* **2005**, 1761–1763.

[19] Bañares, M. A. *Catalysis Today* **2005**, *100*, 71–77.

[20] Weckhuysen, B. M. *Physical Chemistry Chemical Physics* **2003**, *5*, 1.

[21] Chee, S. W.; Lunkenbein, T.; Schlögl, R.; Roldán Cuenya, B. *Chemical Reviews* **2023**, acs.chemrev.3c00352.

[22] Vogt, C.; Weckhuysen, B. M. *Nature Reviews Chemistry* **2022**, *6*, 89–111.

[23] Lyu, D.; Xu, J.; Wang, Z. *Frontiers in Chemistry* **2023**, *11*, 1231886.

[24] Grdadolnik, J. *Acta Chim. Slov.* **2002**,

[25] Cosens, C. R. *Proceedings of the Physical Society* **1934**, *46*, 818–823.

[26] Michels, W. C. *Review of Scientific Instruments* **1938**, *9*, 10–12.

[27] Michels, W. C.; Curtis, N. L. *Review of Scientific Instruments* **1941**, *12*, 444–447.

[28] De Juan, A.; Tauler, R. *Analytica Chimica Acta* **2021**, *1145*, 59–78.

[29] Lawton, W. H.; Sylvestre, E. A. *Technometrics* **1971**, *13*, 617–633.

[30] König, C. F. J. **2012**, *116*, 19857–19866.

[31] Baurecht, D.; Fringeli, U. P. *Review of Scientific Instruments* **2001**, *72*, 3782–3792.

[32] Chiarello, G. L.; Ferri, D. *Physical Chemistry Chemical Physics* **2015**, *17*, 10579–10591.

[33] Franks, L. *Signal Theory, Revisited Edition*, 1st ed.; Dowden & Culver, Inc., 1981.

[34] Vogt, C.; Groeneveld, E.; Kamsma, G.; Nachtegaal, M.; Lu, L.; Kiely, C. J.; Berben, P. H.; Meirer, F.; Weckhuysen, B. M. *Nature Catalysis* **2018**, *1*, 127–134.

[35] Goodall, C. *Technometrics* **1988**, *30*, 351–352.

[36] Jackson, J. E. *A User's Guide To Principal Components*, 2nd ed.; Wiley-Interscience: New Jersey, 2003.

[37] Sawall, M.; Kubis, C.; Barsch, E.; Selent, D.; Börner, A.; Neymeyr, K. *Journal of the Iranian Chemical Society* **2016**, *13*, 191–205.

[38] Bürgi, T.; Baiker, A. *The Journal of Physical Chemistry B* **2002**, *106*, 10649–10658.

[39] Urakawa, A.; Wirz, R.; Bürgi, T.; Baiker, A. *The Journal of Physical Chemistry B* **2003**, *107*, 13061–13068.

[40] Wirz, R.; Bürgi, T.; Lindner, W.; Baiker, A. *Analytical Chemistry* **2004**, *76*, 5319–5330.

[41] Ferri, D.; Kumar, M. S.; Wirz, R.; Eyssler, A.; Korsak, O.; Hug, P.; Weidenkaff, A.; Newton, M. A. *Physical Chemistry Chemical Physics* **2010**, *12*, 5634.

[42] Johnson, R. A.; Wichern, D. W. *Applied Multivariate Statistical Analysis*, 5th ed.; Prentice Hall: Upper Saddle River, N.J, 2002.

[43] De Juan, A.; Jaumot, J.; Tauler, R. *Anal. Methods* **2014**, *6*, 4964–4976.

[44] Pořízka, P.; Klus, J.; Képeš, E.; Prochazka, D.; Hahn, D. W.; Kaiser, J. *Spectrochimica Acta Part B: Atomic Spectroscopy* **2018**, *148*, 65–82.

[45] Lever, J.; Krzywinski, M.; Altman, N. *Nature Methods* **2017**, *14*, 641–642.

[46] Bro, R.; Smilde, A. K. *Anal. Methods* **2014**, *6*, 2812–2831.

[47] Navarete, F.; Damineli, A.; Steiner, J. E.; Blum, R. D. *Monthly Notices of the Royal Astronomical Society* **2021**, *503*, 270–291.

[48] Bian, W.-H.; He, Z.-C.; Green, R.; Shi, Y.; Ge, X.; Liu, W.-S. *Monthly Notices of the Royal Astronomical Society* **2016**, *456*, 4081–4088.

[49] Elliott, M. A.; Walter, G. A.; Swift, A.; Vandenborne, K.; Schotland, J. C.; Leigh, J. S. *Magnetic Resonance in Medicine* **1999**, *41*, 450–455.

[50] Shinn, M. *Proceedings of the National Academy of Sciences* **2023**, *120*, e2311420120.

[51] Lazanas, A. C.; Prodromidis, M. I. *ACS Measurement Science Au* **2023**, *3*, 162–193.

[52] Vivier, V.; Orazem, M. E. *Chemical reviews* **2022**, *122*, 11131–11168.

[53] Beattie, J. R.; Esmonde-White, F. W. L. *Applied Spectroscopy* **2021**, *75*, 361–375.

[54] Golub, G. H. **2000**, *123*, 35–65.

[55] White, P. A. *Journal of the Society for Industrial and Applied Mathematics* **1958**, *6*, 393–437.

[56] Sun, Q. *Vibrational Spectroscopy* **2009**, *51*, 213–217.

[57] Liu, Y.; Zheng, B.; Zhang, T.; Chen, Y.; Liu, J.; Wang, Z.; Gong, X. *Electrochimica Acta* **2022**, *432*, 141201.

[58] Pan, Y.; Liu, Z.; Liu, S.; Qin, L.; Yang, Y.; Zhou, M.; Sun, Y.; Cao, X.; Liang, S.; Fang, G. *Advanced Energy Materials* **2023**, *13*, 2203766.

[59] Buck, U.; Dauster, I.; Gao, B.; Liu, Z.-f. *The Journal of Physical Chemistry A* **2007**, *111*, 12355–12362.

[60] Li, J.; Guo, J.; Dai, H. *Science Advances* **2022**, *8*, 1–12.

[61] Winkler, M. E. G.; Gonçalves, R. H.; Rubira, A. F. *ACS Omega* **2022**, *7*, 45067–45076.

[62] Guo, C.; Guo, Y.; Shi, Y.; Lan, X.; Wang, Y.; Yu, Y.; Zhang, B. *Angewandte Chemie International Edition* **2022**, *61*, e202205909.

[63] Verma, P. K.; Kundu, A.; Puretz, M. S.; Dhoonmoon, C.; Chegwidden, O. S.; Londergan, C. H.; Cho, M. *Journal of Physical Chemistry B* **2018**, *122*, 2587–2599.

[64] Katayama, Y.; Nattino, F.; Giordano, L.; Hwang, J.; Rao, R. R.; Andreussi, O.; Marzari, N.; Shao-Horn, Y. *The Journal of Physical Chemistry C* **2019**, *123*, 5951–5963.

[65] An, H.; De Ruiter, J.; Wu, L.; Yang, S.; Meirer, F.; Van Der Stam, W.; Weckhuysen, B. M. *JACS Au* **2023**, *3*, 1890–1901.

[66] Zhu, S.; Jiang, B.; Cai, W. B.; Shao, M. *Journal of the American Chemical Society* **2017**, *139*, 15664–15667.

[67] Park, K.; Kim, Y.; Lee, K. J. *Journal of Radioanalytical and Nuclear Chemistry* **2019**, *322*, 487–493.

[68] Winkler, D.; Leitner, M.; Auer, A.; Kunze-Liebhäuser, J. *ACS Catalysis* **2024**, 1098–1106.

[69] Yu, C.-C.; Chiang, K.-Y.; Okuno, M.; Seki, T.; Ohto, T.; Yu, X.; Korepanov, V.; Hamaguchi, H.-o.; Bonn, M.; Hunger, J.; Nagata, Y. *Nature Communications* **2020**, *11*, 5977.

[70] Seki, T.; Chiang, K.-Y.; Yu, C.-C.; Yu, X.; Okuno, M.; Hunger, J.; Nagata, Y.; Bonn, M. *The Journal of Physical Chemistry Letters* **2020**, *11*, 8459–8469.

[71] Moradzaman, M.; Mul, G. *ACS Catalysis* **2020**, *10*, 8049–8057.

[72] Hsu, J.; Eid, A. M.; Randall, C.; Houache, M. S.; Abu-Lebdeh, Y.; Al-Abadleh, H. A. *Langmuir* **2022**, *38*, 14789–14798.

[73] Deng, W.; Yuan, T.; Chen, S.; Li, H.; Hu, C.; Dong, H.; Wu, B.; Wang, T.; Li, J.; Ozin, G. A.; Gong, J. *Fundamental Research* **2021**, *1*, 432–438.

[74] Dunwell, M.; Yang, X.; Setzler, B. P.; Anibal, J.; Yan, Y.; Xu, B. *ACS Catalysis* **2018**, *8*, 3999–4008.

[75] Figueiredo, M. C.; Ledezma-Yanez, I.; Koper, M. T. M. *ACS Catalysis* **2016**, *6*, 2382–2392.

[76] Zhu, S.; Li, T.; Cai, W. B.; Shao, M. *ACS Energy Letters* **2019**, *4*, 682–689.

[77] Pérez-Gallent, E.; Figueiredo, M. C.; Calle-Vallejo, F.; Koper, M. T. M. *Angewandte Chemie* **2017**, *129*, 3675–3678.

[78] Schumacher, H.; Ku¨nzelmann, U.; Ku¨nzelmann, K.; Vasilev, B.; Eichhorn, K.-J.; Bartha, J. W. *Applied Spectroscopy* **2010**, *64*, 1022–1027.

[79] Huang, B. et al. *JACS Au* **2021**, *1*, 1674–1687.

[80] Pansini, F. N.; Varandas, A. J. *Chemical Physics Letters* **2022**, *801*, 139739.

[81] Ataka, K.-i.; Osawa, M. *Langmuir* **1998**, *14*, 951–959.

[82] Ataka, K.-i.; Yotsuyanagi, T.; Osawa, M. *The Journal of Physical Chemistry* **1996**, *100*, 10664–10672.

[83] Odendahl, N. L.; Geissler, P. L. *Journal of the American Chemical Society* **2022**, *144*, 11178–11188.

[84] Brubach, J. B.; Mermet, A.; Filabozzi, A.; Gerschel, A.; Roy, P. *Journal of Chemical Physics* **2005**, *122*, 1845091–1845097.

[85] Bakker, H. J. *Chemical Reviews* **2008**, *108*, 1456–1473.

[86] White, D. W.; Mastrapa, R. M.; Sandford, S. A. *Icarus* **2012**, *221*, 1032–1042.

[87] Isokoski, K.; Poteet, C. A.; Linnartz, H. *Astronomy & Astrophysics* **2013**, *555*, A85.

[88] Chou, T. C.; Chang, C. C.; Yu, H. L.; Yu, W. Y.; Dong, C. L.; Velasco-Vélez, J. J.; Chuang, C. H.; Chen, L. C.; Lee, J. F.; Chen, J. M.; Wu, H. L. *Journal of the American Chemical Society* **2020**, *142*, 2857–2867.

[89] Kim, Y.; Park, S.; Shin, S.-J.; Choi, W.; Min, B. K.; Kim, H.; Kim, W.; Hwang, Y. J. *Energy & Environmental Science* **2020**, *13*, 4301–4311.

[90] Zimmermann, J.; Thielges, M. C.; Yu, W.; Dawson, P. E.; Romesberg, F. E. *The Journal of Physical Chemistry Letters* **2011**, *2*, 412–416.

[91] Chin, J. K.; Jimenez, R.; Romesberg, F. E. *Journal of the American Chemical Society* **2001**, *123*, 2426–2427.

[92] Litvak, I.; Anker, Y.; Cohen, H. *RSC Advances* **2018**, *8*, 28472–28479.

[93] Wuttig, A.; Liu, C.; Peng, Q.; Yaguchi, M.; Hendon, C. H.; Motobayashi, K.; Ye, S.; Osawa, M.; Surendranath, Y. *ACS Central Science* **2016**, *2*, 522–528.

[94] Iwasita, T.; Rodes, A.; Pastor, E. *Journal of Electroanalytical Chemistry* **1995**, *383*, 181–189.

[95] Arihara, K.; Kitamura, F.; Ohsaka, T.; Tokuda, K. *Journal of Electroanalytical Chemistry* **2001**, *510*, 128–135.

[96] Hori, Y. *J. Chem. Soc.* **1988**, 17–19.

[97] Hayden, B.; Prince, K.; Woodruff, D.; Bradshaw, A. *Surface Science* **1983**, *133*, 589–604.

[98] Heyes, J.; Dunwell, M.; Xu, B. *The Journal of Physical Chemistry C* **2016**, *120*, 17334–17341.

[99] Timoshenko, J.; Bergmann, A.; Rettenmaier, C.; Herzog, A.; Arán-Ais, R. M.; Jeon, H. S.; Haase, F. T.; Hejral, U.; Grosse, P.; Kühl, S.; Davis, E. M.; Tian, J.; Magnussen, O.; Roldan Cuenya, B. *Nature Catalysis* **2022**, *5*, 259–267.

[100] Yang, Y. et al. *Nature* **2023**, *614*, 262–269.

[101] An, H.; Wu, L.; Mandemaker, L. D.; Yang, S.; de Ruiter, J.; Wijten, J. H.; Janssens, J. C.; Hartman, T.; van der Stam, W.; Weckhuysen, B. M. *Angewandte Chemie - International Edition* **2021**, *60*, 16576–16584.

[102] Simon, G. H.; Kley, C. S.; Roldan Cuenya, B. *Angewandte Chemie - International Edition* **2021**, *60*, 2561–2568.

[103] Clark, E. L.; Resasco, J.; Landers, A.; Lin, J.; Chung, L.-T.; Walton, A.; Hahn, C.; Jaramillo, T. F.; Bell, A. T. *ACS Catalysis* **2018**, *8*, 6560–6570.

[104] Dunwell, M.; Lu, Q.; Heyes, J. M.; Rosen, J.; Chen, J. G.; Yan, Y.; Jiao, F.; Xu, B. *Journal of the American Chemical Society* **2017**, *139*, 3774–3783.

[105] Deng, B.; Huang, M.; Zhao, X.; Mou, S.; Dong, F. *ACS Catalysis* **2022**, *12*, 331–362.

[106] Velasco-Vélez, J. J.; Chuang, C. H.; Gao, D.; Zhu, Q.; Ivanov, D.; Jeon, H. S.; Arrigo, R.; Mom, R. V.; Stotz, E.; Wu, H. L.; Jones, T. E.; Roldan Cuenya, B.; Knop-Gericke, A.; Schlögl, R. *ACS Catalysis* **2020**, *10*, 11510–11518.

[107] Grosse, P.; Yoon, A.; Rettenmaier, C.; Herzog, A.; Chee, S. W.; Roldan Cuenya, B. *Nature Communications* **2021**, *12*, 1–11.

[108] Wang, J. et al. *ACS Materials Letters* **2023**, *5*, 3212–3221.

[109] Ojha, K.; Arulmozhi, N.; Aranzales, D.; Koper, M. T. M. *Angewandte Chemie International Edition* **2020**, *59*, 711–715.

[110] Lust, E. *Encyclopedia of Interfacial Chemistry*; Elsevier, 2018; pp 316–344.

[111] Hinsch, J. J.; White, J. J.; Wang, Y. *Computational and Theoretical Chemistry* **2024**, *1232*, 114462.
